# Accounting for location uncertainty in azimuthal telemetry data improves ecological inference

**DOI:** 10.1186/s40462-018-0129-1

**Published:** 2018-07-25

**Authors:** Brian D. Gerber, Mevin B. Hooten, Christopher P. Peck, Mindy B. Rice, James H. Gammonley, Anthony D. Apa, Amy J. Davis

**Affiliations:** 1 Colorado Cooperative Fish and Wildlife Research Unit, Department of Fish, Wildlife, and Conservation Biology, Colorado State University, Fort Collins, 80523 CO USA; 2 Department of Natural Resources Science, University of Rhode Island, 1 Greenhouse Road, Kingston, 02881-2018 RI USA; 3 U.S. Geological Survey, Colorado Cooperative Fish and Wildlife Research Unit, Departments of Fish, Wildlife, and Conservation Biology and Statistics, Colorado State University, Fort Collins, 80523 CO USA; 4 Colorado Division of Parks and Wildlife, 317 West Prospect, Fort Collins, 80526 CO USA; 5 Colorado Division of Parks and Wildlife, 711 Independent Avenue, Grand Junction, 81505 CO USA; 6 National Wildlife Research Center, United States Department of Agriculture, 4101 Laporte Avenue, Fort Collins, 80521 CO USA

**Keywords:** Home range, Location uncertainty, Radio-telemetry, Radio, Resource selection function, Telemetry, VHF

## Abstract

**Background:**

Characterizing animal space use is critical for understanding ecological relationships. Animal telemetry technology has revolutionized the fields of ecology and conservation biology by providing high quality spatial data on animal movement. Radio-telemetry with very high frequency (VHF) radio signals continues to be a useful technology because of its low cost, miniaturization, and low battery requirements. Despite a number of statistical developments synthetically integrating animal location estimation and uncertainty with spatial process models using satellite telemetry data, we are unaware of similar developments for azimuthal telemetry data. As such, there are few statistical options to handle these unique data and no synthetic framework for modeling animal location uncertainty and accounting for it in ecological models.

We developed a hierarchical modeling framework to provide robust animal location estimates from one or more intersecting or non-intersecting azimuths. We used our azimuthal telemetry model (ATM) to account for azimuthal uncertainty with covariates and propagate location uncertainty into spatial ecological models. We evaluate the ATM with commonly used estimators (Lenth (1981) maximum likelihood and M-Estimators) using simulation. We also provide illustrative empirical examples, demonstrating the impact of ignoring location uncertainty within home range and resource selection analyses. We further use simulation to better understand the relationship among location uncertainty, spatial covariate autocorrelation, and resource selection inference.

**Results:**

We found the ATM to have good performance in estimating locations and the only model that has appropriate measures of coverage. Ignoring animal location uncertainty when estimating resource selection or home ranges can have pernicious effects on ecological inference. Home range estimates can be overly confident and conservative when ignoring location uncertainty and resource selection coefficients can lead to incorrect inference and over confidence in the magnitude of selection. Furthermore, our simulation study clarified that incorporating location uncertainty helps reduce bias in resource selection coefficients across all levels of covariate spatial autocorrelation.

**Conclusion:**

The ATM can accommodate one or more azimuths when estimating animal locations, regardless of how they intersect; this ensures that all data collected are used for ecological inference. Our findings and model development have important implications for interpreting historical analyses using this type of data and the future design of radio-telemetry studies.

**Electronic supplementary material:**

The online version of this article (10.1186/s40462-018-0129-1) contains supplementary material, which is available to authorized users.

## Background

Understanding animal space-use and its implications for population and community dynamics is a central component of ecology and conservation biology [1, 2]. Coupling environmental characteristics of where animals are found (and not found) provides important insights into species-habitat relationships, which is fundamental to understanding a species’s ecological niche [3]. For vagile animals, characterizing space-use during different life-history stages can help elucidate stage-specific habitat area requirements, dispersal patterns, or site-fidelity associations. Perhaps two of the most common objectives related to animal space-use are describing the home range [4] area and configuration and how animals selectively use spatially-explicit resources relative to their availability (i.e., resource selection functions (RSF); [2, 5]). RSFs provide insights into how landscape features affect animal behavior and habitat associations, and thus potentially limiting factors on population dynamics. Generally, animal spatial relationships provide critical information to land-use planners and conservation decision makers, making it vital that inferences are made correctly.

The need to understand animal spatial relationships has led to the increasing refinement and utility of telemetry devices [6]. Traditional telemetry data were solely collected using VHF (“very high frequency”) radio signals to track individual animals with radio tags; VHF radio-telemetry started around the mid-1960s and is still often employed. Location data are often collected by observers recording azimuths in the direction of the radio signal from known locations, but also by direct observation after walking up to a radio-tagged animal. The use of azimuths is attractive because it can reduce disturbance to the animal and also can reduce observer effort and thus increase the number of locations possible. Azimuths are often made using hand-held receivers, but also vehicle mounted receivers, or fixed towers [7]. A limitation of VHF telemetry is that there is a maximum distance to which animals can be detected, such that animals that leave the area may never be found, and large movements may be missed, thus possibly mischaracterizing the home range. This limitation can be alleviated by attaching VHF technology to a fixed wing aircraft and homing in on animal locations. However, the major limitation of VHF telemetry is that obtaining azimuths or locations requires considerable effort by the researcher, thus generally leading to limited spatial datasets. Modern telemetry technology used to track animals over large spatial areas and obtain extensive datasets is done by using Argos satellites, the global positioning system (GPS), or cell phone tower technology. While these newer forms of telemetry data can be beneficial, radio-telemetry devices are still relatively inexpensive. Radio technology also typically have low energy requirements, which allows for miniaturized and long-lasting devices to be fixed to small and volant animals for obtaining high spatial resolution data with minimal risk to incurring costs on survival and movement [8]. More so, digital VHF is quickly becoming an important way to monitor the movements of small-bodied species at regional scales [9]. This technology does not rely on directional signaling, but instead signals detected at radio towers.

It is well recognized that spatial locations from telemetry devices are not without error and estimation uncertainty [10, 11]. Observed locations contain measurement errors, or deviations between the recorded telemetry location and the true location of the animal. The magnitude of these deviations and the shape or structure of spatial location uncertainty is often specific to the type of telemetry technology [12] and the environmental conditions [7, 10, 13, 14]. Failing to account for location uncertainty can have pernicious impacts on spatial analyses of animal resource selection [15, 16], distribution [17], and movement modeling [2, 18, 19]. We are careful to distinguish between measurement error and location uncertainty. Location uncertainty is commonly referred to as error, which often implies additive and Gaussian uncertainty, neither of which are usually true for animal spatial analyses. While telemetry location uncertainty may sometimes be modeled as a multivariate Gaussian process [20], it is often much more complex [12]. In contrast, measurement error is explicitly the deviation between truth and the observed data, such as an azimuth or location.

Recent model developments focusing on satellite-based telemetry data (e.g., GPS, Argos) have highlighted the importance of appropriately characterizing location uncertainty using hierarchical modeling techniques to synthetically incorporate uncertainty into ecological process models (e.g., RSF: [21]; Movement analyses: [22, 23]). These developments have been in part to accommodate complex data structures due to the high density of location data that are obtained with these technologies, necessitating models that account for autocorrelation through movement processes. Similar developments that address the unique issues of azimuthal telemetry data do not exist. Since VHF telemetry data are mostly collected at coarse temporal scales with only a few locations per animal per day, the data structure is simpler and more likely meets the independence assumptions of commonly used ecological models. However, regardless of the quantity of data or type of telemetry technology, characterizing uncertainty is paramount for proper ecological inference and is flexibly handled by hierarchical modeling [2].

In fact, there have been few model developments to improve animal location estimation or uncertainty in the recent decades [24, 25]. Standard practice is to analyze azimuthal data using Lenth’s (1981) maximum likelihood estimator (MLE) or weighted MLE (M-estimators) to reduce the influence of outliers. The estimators are implemented in the software LOCATE [26] and LOAS (Ecological Software Solutions LLC, Sacramento, California). Spatial location estimates are then commonly used in a secondary ecological model, in which the location uncertainty is ignored and possibly unreported [15, 27, 28]. More so, location estimates may also be dropped due to estimation issues, thus a loss of information, or the magnitude of the uncertainty is used to define the scale of inference, rather than the ecological question [15, 27, 28]. These approaches raise several concerns.

Foremost is that these practices degrade ecological inference by disregarding uncertainty, censoring data, or altering the scale of inference. Second, uncertainty from Lenth’s MLE or M-estimators are commonly defined using confidence ellipses based on the assumption of asymptotic normality [7]. Assuming the uncertainty is strictly elliptical (e.g., multivariate Gaussian) may be overly restrictive and thus misrepresenting the true uncertainty. Empirical evidence indicates that 95% confidence ellipses of Lenth’s MLE or M-estimators cover the true location much less than 95% of the time (between 39% and 70%; [7]). There are also concerns raised by Lenth (1981) over the validity of the variance-covariance matrix of the M-estimators. Last, additional improvements could add flexibility in how researchers approach the design of radio-telemetry studies. For example, Lenth’s estimators cannot estimate locations when azimuths do not intersect, or estimate uncertainty when only two azimuths are collected. It is also possible for the estimator to fail with three or more azimuths, resulting in the use of a secondary estimator (i.e., a component-wise average of all azimuthal intersections) that has no measure of uncertainty or robust statistical properties.

Furthermore, it is well known that radio-signal direction can be influenced by many factors, including vegetation, terrain, animal movement, observer experience, and the distance between the observer and the animal [6, 7]. To accommodate these factors, standard practice has been to test observers taking azimuths on known locations of a radio-signal to experimentally quantify telemetry error. Observer error can then be applied to estimate location uncertainty via error polygons and confidence ellipses [28]. If field trials obtain data across known influencing factors, a model can be developed to incorporate variation in telemetry error for these conditions [29]. However, field trials will always be limited in their ability to anticipate all combinations of influential factors when collecting radio-telemetry data. Also, there are inconsistent recommendations in the literature regarding how best to estimate location uncertainty ([7]; i.e., Error polygons vs. Lenth’s confidence ellipses). We developed an approach that accommodates pre-existing data sources, where field trials may not be available; if these data are available, they can be incorporated.

We developed hierarchical azimuthal telemetry models (ATM) that estimate animal locations with uncertainty, which can be synthetically propagated into spatial ecological models. We first describe a novel Bayesian ATM, which models azimuthal uncertainty using covariates. Second, we evaluate the ATM and Lenth’s estimators under a variety of study designs; model development is motivated by a VHF telemetry study on the threatened Gunnison sage-grouse (*Centrocercus minimus*; [30]), which we use to setup the simulation and explore observer effects using the ATM. Third, we develop hierarchical spatial models for azimuthal data, including a home range and RSF analysis, which we fit to azimuthal data collected on the Gunnison sage-grouse; see Additional file 1 for species background information and study details. Last, we examine how ignoring location uncertainty can affect ecological inference through these empirical examples, but also more generally by conducting an RSF simulation.

## Methods

## Azimuthal telemetry model (ATM)

Suppose that multiple individuals (*l*=1,…,*L*) are each fitted with a radio-transmitter and are subsequently relocated on certain days (*i*=1,…,*N*_*l*_). For each relocation, an observer records a set of azimuths (*θ*_*lij*_;*j*=1,…,*J*_*li*_) at known locations **z**_*lij*_≡(*z*_1*lij*_,*z*_2*lij*_)^′^ to estimate the individual’s spatial location, ***μ***_*li*_≡(*μ*_1*li*_,*μ*_2*li*_)^′^. Following [24, 25], we consider the observer locations as a fixed part of the study design and the azimuthal data observed with some uncertainty, which can be described by a circular probability distribution [31]. We use the von Mises distribution and a trigonometric link function to relate the true animal location with the data,

1 $$   \begin{aligned} \text{Observation Process:} \hspace{0.25 in} &\theta_{lij} \sim \text{von Mises}\left(\tilde{\theta}_{lij}, \kappa_{lij}\right)\\ \text{Link Function:} \hspace{0.25 in} &\tilde{\theta}_{lij} = \text{tan}^{-1}\left(\frac{\mu_{2li}-z_{2lij}}{\mu_{1li}-z_{1lij}}\right). \\ \end{aligned}  $$

Uncertainty in the azimuthal data model is controlled by the concentration parameter *κ* (typically in radians), in which larger values indicate less uncertainty (see Additional file 2: Figure S1). The parameter *κ* can be functionally modeled via covariates (e.g., observer effects or study year; defined by the matrix **w**_*lij*_) in a hierarchical structure that accommodates both hypothesized effects and unmodeled heterogeneity based on the variance parameter $\sigma ^{2}_{\kappa }$,

2 $$ \begin{aligned} \text{log}(\kappa_{lij}) &\sim \mathrm{N}\left(\mathbf{w}_{lij}'\boldsymbol{\beta}, \sigma^{2}_{\kappa}\right).\\ \end{aligned}  $$

Using this framework, we can also model azimuthal uncertainty as a function of distance between the animal and observer, which was previously not possible outside of field trials. For example, we could include the distance of the observer from the radio-signal as (*d*_*lij*_; distance effect of *α*_1_),

3 $$ \begin{aligned} \text{log}(\kappa_{lij}) &\sim \mathrm{N}\left(\mathbf{w}_{lij}'\boldsymbol{\beta} + \alpha_{1} \text{log}\left(d_{lij} \right), \sigma^{2}_{\kappa}\right)\\ \text{log}\left(d_{lij} \right) &= \sqrt{(\boldsymbol{z}_{lij}-\boldsymbol{\mu}_{li})'(\boldsymbol{z}_{lij}-\boldsymbol{\mu}_{li})}, \\ \end{aligned}  $$

or perhaps include terrain complexity or habitat structure at observer locations to model radio-signal bounce and general site-level variability.

To complete the Bayesian model formulation, we specify priors for our unknown parameters. Commonly used priors are ***β***∼N(***μ***_*β*_,***Σ***_*β*_) and $\sigma ^{2}_{\kappa } \sim \text {IG}(\alpha _{\sigma }, \beta _{\sigma })$. The prior for ***μ***_*li*_ may be specified a number of ways, including multivariate Gaussian. However, to increase computational efficiency when fitting the model, it is advantageous to define an upper bound to the distance for which a telemetered individual can be detected. Otherwise, in cases where a limited number of azimuths are available or azimuths do not intersect (e.g., parallel azimuths), a multivariate Gaussian distribution will allow the uncertainty to theoretically propagate over an infinite spatial domain. In what follows, we specify a fixed maximum distance from each observer location to the animal location, using radius *r*. We also define a diffuse prior density for each spatial location as the union of all circles of the *j*^*th*^ observer location with radius *r* where **v** are coordinates (x, y) in the spatial domain,

4 $$ \begin{aligned} \boldsymbol{\mu}_{li} \sim \text{Unif}\left(\bigcup\limits_{j=1}^{J_{li}}\left\{\mathbf{v}\,|\, \|\mathbf{v}-\mathbf{z}_{lij}\|^{2}_{2} \leq r^{2}\right\}\right). \end{aligned}  $$

We fit the ATM using a Markov chain Monte Carlo (MCMC) algorithm written in R and C++ ([32]; see Additional file 3; an R package, ‘razimtuh’ can be downloaded at https://github.com/cppeck/razimuth). A specialized MCMC algorithm was preferred over using Bayesian model fitting software, such as JAGS (Just Another Gibbs Sample), to ensure computational efficiency and reliability for all sizes of data. First, we fit simulated data to examine a wide range of conditions, including one or more intersecting or non-interesting azimuths. Second, using the Gunnison sage-grouse telemetry data from two observers, we fit the ATM (Eqs. 1 and 2) to investigate possible group-level differences in *κ* between observers and variation within observers.

### Radio-telemetry simulation

We evaluated the performance of the ATM and Lenth’s MLE and M-estimators (Andrews and Huber) along with a simple component-wise average of intersections. We did so by simulating known location data under two common radio-telemetry study designs (road and encircle) and a more variable approach (random). Under the random design, each observer location was defined by a random azimuth from the true animal location sampled uniformly from −*π* to *π* and the distance was sampled randomly from the empirical distances estimated from the Gunnison sage-grouse data (Additional file 2: Figure S2). Under the encircle design, the initial observer location is sampled the same as the random design, but subsequent observer locations are sampled uniformly from 30−60° from the previous observer location and true animal location; each observer distance is also randomly sampled from the sage-grouse distance distribution. In effect, this places observers such that they encircle the animal location. Last, the road design constrains the observer locations to a linear feature, thus limiting the angular differences among azimuths. For each design, we considered scenarios of 3 or 4 azimuths per location and moderate and high azimuth uncertainty (*κ* = 100 or 25, respectively). Simulation algorithms are provided in Additional file 3 and R code in Additional file 4. The ATM, assuming a homogeneous *κ*, was fit using MCMC and posterior properties were based on 50,000 iterations. Lenth’s MLE and M-estimators were fit using Lenth’s original algorithms [24]. Lenth’s MLE was also fit using the R package ‘sigloc’ [33]. sigloc is the only R package we are aware of that estimates Lenth’s MLE, but it does not use the algorithm suggested by Lenth (1981), but rather a quasi-Newton optimization algorithm which Lenth (1981) suggested avoiding.

### Spatial models for azimuthal data

#### Home range

Given our new telemetry data model, we can now analyze our estimated animal spatial locations using any ecological process model. A simple application is home range estimation, which has often been done using a convex hull or non-parametric kernel density estimation [2, 34]. We can propagate location uncertainty using the ATM by treating the home range estimate as a derived quantity. For a given individual that was relocated *n* times within a season, we can estimate their seasonal home range for the *k*^th^ iteration of MCMC using the 95% isopleth of the kernel function,

5 $$ \begin{aligned} \hat{f}(\mathbf{c}) =& \frac{\sum_{i=1}^{n} \mathrm{g}\left(\left(c_{1}-\mu_{1i}^{(g)}\right)/b_{1}\right)\mathrm{g}\left(\left(c_{2}-\mu_{2i}^{(k)}\right)b_{2}\right)}{{nb}_{1}b_{2}}, \end{aligned}\vspace*{-8pt}  $$

evaluated at locations of interest ***c***≡(*c*_1_,*c*_2_)^′^, choice of kernel function g (·), and bandwidth parameters *b*_1_ and *b*_2_ (which we constrain as *b*_1_=*b*_2_). The result is a posterior distribution of the 95% home range isopleth, which could be used to further derive a posterior distribution of the home range area, thus fully incorporating all uncertainties in our estimate. We fit the ATM and derived a convex hull and kernel density home range for six individual Gunnison sage-grouse for different seasons (breeding and summer) across all years of available data (2005-2010). We compared these results with home range estimates using estimated locations from Lenth’s MLE, thus ignoring location uncertainty.

#### Resource selection analysis

Another common use of telemetry data is to estimate an RSF. To make inference on the relative selection of spatial resources for the population of radio-tagged individuals, we use a spatial point process, assuming independence among spatial locations [2]. Let **x** be a vector of covariates associated with location ***μ***_*li*_ and individual availability defined by the function *f*_*A*_ and availability coefficients ***θ***. Individual-level selection coefficients (***γ***) are realizations from a population-level selection process with mean and covariance (***μ***_*γ*_,***Σ***_*γ*_, respectively; [35]). For multiple individuals, the hierarchical RSF model is specified as,

6 $$ \begin{aligned} &{}\text{Inhomogeneous point-process:} \\ &\qquad\quad [\boldsymbol{\mu}_{li}| \boldsymbol{\gamma}, \boldsymbol{\theta}] \equiv \frac{\text{exp}(\mathbf{x}'(\boldsymbol{\mu}_{li})\boldsymbol{\gamma}) f_{A}(\boldsymbol{\mu}_{li}, \boldsymbol{\theta})}{\int \text{exp}(\mathbf{x}'(\boldsymbol{\mu})\boldsymbol{\gamma})f_{A}(\boldsymbol{\mu}, \boldsymbol{\theta})d\boldsymbol{\mu}},\\ &{}\text{Individual-level coefficients:}\\ &\qquad\qquad\qquad \boldsymbol{\gamma}\!\sim\! \mathrm{N}(\boldsymbol{\mu}_{\gamma},\boldsymbol{\Sigma}_{\gamma}),\\ &{}\text{Priors:} \qquad\qquad\boldsymbol{\mu}_{\gamma}\! \sim \!\mathrm{N}(\boldsymbol{\mu}_{0}, \boldsymbol{\Sigma}_{0}), \hspace{0.1 in} \!\!\boldsymbol{\Sigma}^{-1}_{\gamma} \!\sim\! \text{Wish}\left((\!\boldsymbol{\mathcal{S}}\!\nu\!)^{-1},\! \nu\right)\!.  \end{aligned}  $$

We fit the ATM-RSF model to the same individual Gunnison sage-grouse from the home-range analysis using data from the summer months (16 July to 30 September, from 2005 to 2009). We use these individuals as exemplars to compare estimated regression coefficients from the ATM-RSF with estimates from the same RSF, but we assumed location estimates from Lenth’s MLE are known without uncertainty. We include six common spatial variables to model resource selection of the Gunnison sage-grouse (Additional file 1; [30]): road density, distance to highway, distance to wetlands, distance to conservation easements, elevation, and vegetation classification (i.e., grassland, agriculture). In addition to including both categorical and continuous spatial covariates, the variables include a highly variable topographic variable (i.e., elevation) and more smoothly continuous measures of distance to features. The structure of each type and how variable values are from neighboring locations could differently impact RSF inference by the scale and shape of animal location uncertainties [36].

We assumed uniform spatial availability (*f*_*A*_(·)) for an individual animal in two ways: 1) by defining a large study area region and 2) by using the convex hull of all locations (***μ***_*li*_). The first focuses on a first-order selection process within the broader landscape and the second focuses on the second-order selection process within an individual’s area of use [37]. In addition to producing fundamentally different inference for resource selection, the location uncertainty affects each process differently. For the study area region, resource selection is subject to only location uncertainty, whereas for convex hull availability for an individual, resource selection is subject to both location and availability uncertainty.

For a more general understanding, we conducted a simulation to explore the connection among location uncertainty, covariate spatial heterogeneity, and ecological inference in RSF analyses. Previous work has demonstrated that the size of telemetry error and the resolution and heterogeneity of spatial covariates can affect the quality of ecological inference from RSFs [36]; we further this understanding by examining how varying levels of spatial autocorrelation of a continuous and categorical covariate at different sample sizes and spatial resolution affects RSF coefficients when incorporating and ignoring location uncertainty, compared to knowing the true locations. Specifically, we simulated animal location data (*N*_locations_= 50, 200) that coincide with covariate values of low, moderate, and high spatial autocorrelation, defined using a Gaussian random field (covariates at 25 m or 100 m resolution; Additional file 5). Observations were three azimuths per location, simulated under a random design (Additional file 3), with moderate azimuthal uncertainty (*κ*= 50). We fit these data with 1) the ATM-RSF, and 2) a typical RSF model that used location estimates from Lenth’s (1981) MLE, ignoring location uncertainty. We compared coefficient estimates from these approaches across simulations with that of fitting an RSF where the true locations are known, providing a reference to the best case scenario for these data.

## Results

### ATM

**Fig. 1:**
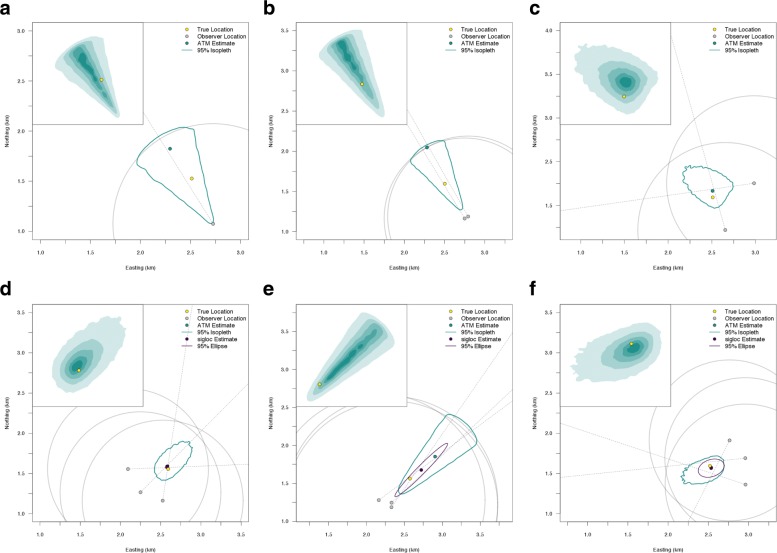
Illustrative examples of spatial location estimates from the azimuthal telemetry model (ATM) and Lenth (1981) maximum likelihood estimator fit using the ‘sigloc’ package. The union of the gray circles are the region of uniform prior probability density for the spatial location (***μ***_*i*_). Azimuthal uncertainty is defined as *κ* = 25. The inset shows the posterior distribution from the ATM at isopleths of 10, 25, 50, 75, and 95%. Plots that do not contain a sigloc estimate or uncertainty ellipse are because of estimation failure. **a** One Azimuth. **b** Two Non-Intersecting Azimuths. **c** Two Obtuse Azimuths. **d** Three Azimuths with Tight Intersections. **e** Three Acute Azimuths. **f** Three Azimuths with Poor Intersections

**Fig. 2:**
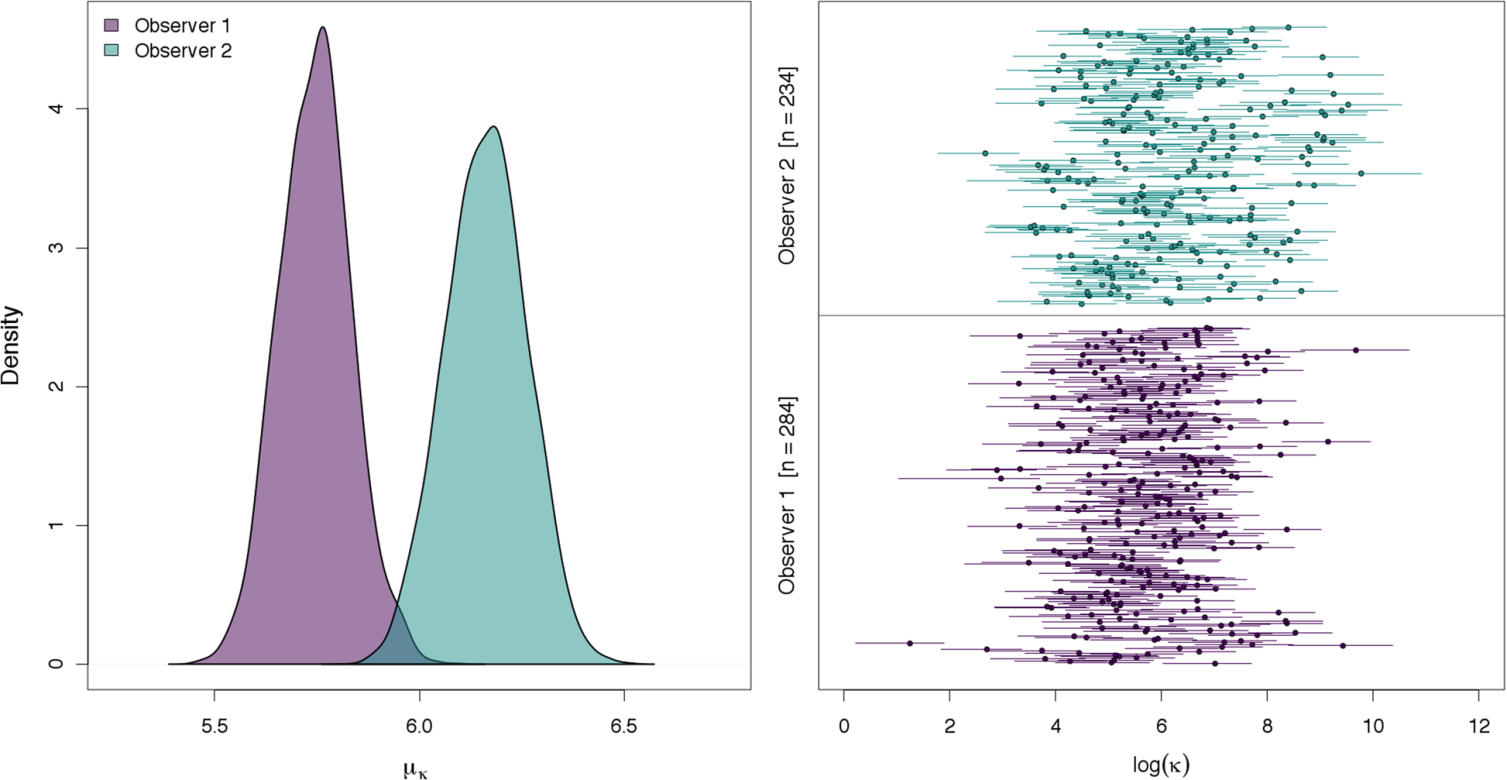
Posterior distributions of estimated observer effects on azimuthal telemetry uncertainty (left, *κ*) and individual location *κ* (right; circles are medians of the posterior distribution and bars are 95% credible intervals) for Gunnison sage-grouse data in 2009

The ATM can be used to fit all combinations and geometries of azimuthal data (Fig. 1 and Additional file 2: Figure S3). It can provide spatial location estimates with measures of uncertainty for single azimuths, multiple non-intersecting azimuths, and acute (< 90°) or obtuse (> 90°) intersecting azimuths. We found the shape of the location uncertainty to be irregular and depend strongly on the number of azimuths and their associated level of uncertainty (Additional file 2: Figure S3). Generally, the precision of animal location estimates depends on the number of azimuths and whether these azimuths intersect each other or not. Using the Gunnison sage-grouse data, we found observer two was generally more precise than observer 2 (Fig. 2). Both observers had a high degree of variability in their azimuthal precision. For example, the range of estimated *κ* was from 4.8 to 13,000 for observer one (See Additional file 2: Figure S1 to visualize *κ* variation). These results demonstrate how we can accommodate heterogeneity in *κ* to improve our understanding of the factors that influence location uncertainty and provide more reliable inference in radio-telemetry studies.

### Radio-telemetry simulation

Across scenarios, we found that locations were typically estimated from all models and estimators, except for sigloc, which had a success rate from 52 to 99%, depending on the scenario (Table 1). The ATM and simple average of intersections always produced a location estimate. Point estimates were more accurate under the encircle study design and under moderate azimuthal uncertainty; accuracy improved 1.5 to 2.5 times with four azimuths compared to three. For all scenarios, point estimates were mostly similar among the different models and estimators. However, sigloc was less accurate than the others under the random and road designs when azimuthal uncertainty was high. The most important difference we found was that of coverage of the true value. All approaches produced relatively poor coverage (0.3 to 0.6, range) except for the ATM, which proved to be slightly below nominal coverage (≈ 90% coverage of true value); coverage improved with an increasing number of azimuths and values of *κ*.

**Table 1:** Simulation findings comparing the azimuthal telemetry model (ATM) with the average of the component-wise azimuth intersections (simple) and Lenth (1981) maximum likelihood estimator (Lenth, sigloc) and M-estimators (Andrews, Huber)

		*κ*=100						*κ*=25					
		Simple	sigloc	Lenth	Huber	Andrews	ATM	Simple	sigloc	Lenth	Huber	Andrews	ATM
Encircle design comparison:													
*n*_*θ*_=3	$n_{\hat {\boldsymbol {\mu }}}$	600	597	600	600	600	600	600	581	598	600	600	600
	*d*_0.5_ (m)	22.4	20.8	20.5	20.4	20.4	21.0	45.3	43.6	42.7	42.8	43.5	42.8
	Coverage	–	0.430	0.432	0.432	0.433	0.888	–	0.422	0.425	0.423	0.435	0.858
*n*_*θ*_=4	$n_{\hat {\boldsymbol {\mu }}}$	600	539	600	600	600	600	600	470	592	595	599	600
	*d*_0.5_ (m)	9.9	9.3	8.7	8.7	8.8	8.7	19.2	19.6	17.5	17.6	17.4	17.5
	Coverage	–	0.575	0.592	0.585	0.592	0.923	–	0.553	0.542	0.538	0.541	0.917
Random design comparison:													
*n*_*θ*_=3	$n_{\hat {\boldsymbol {\mu }}}$	600	533	595	593	593	600	600	439	564	561	566	600
	*d*_0.5_ (m)	32.6	32.2	25.1	25.1	25.3	25.0	62.9	75.1	54.2	53.4	53.7	55.6
	Coverage	–	0.403	0.418	0.417	0.417	0.883	–	0.328	0.348	0.348	0.352	0.850
*n*_*θ*_=4	$n_{\hat {\boldsymbol {\mu }}}$	600	454	594	594	598	600	600	367	573	573	579	600
	*d*_0.5_ (m)	14.2	13.0	9.9	10.0	9.9	10.0	25.3	34.0	19.5	19.6	20.0	20.3
	Coverage	–	0.559	0.581	0.567	0.572	0.920	–	0.526	0.560	0.550	0.556	0.912
Road design comparison:													
*n*_*θ*_=3	$n_{\hat {\boldsymbol {\mu }}}$	600	499	593	593	597	600	600	409	573	571	576	600
	*d*_0.5_ (m)	56.7	44.4	39.0	39.0	38.5	40.5	95.5	110.4	85.1	84.6	83.9	86.4
	Coverage	–	0.397	0.418	0.418	0.412	0.877	–	0.296	0.316	0.310	0.312	0.822
*n*_*θ*_=4	$n_{\hat {\boldsymbol {\mu }}}$	600	443	600	600	600	600	600	316	592	593	595	600
	*d*_0.5_ (m)	53.8	33.4	26.9	27.7	28.1	26.5	90.3	83.5	54.6	54.8	55.2	55.8
	Coverage	–	0.580	0.618	0.595	0.588	0.923	–	0.487	0.561	0.543	0.545	0.883

‘sigloc’ implements Lenth’s maximum likelihood estimator via an alternative algorithm than suggested by Lenth (1981)

*Notes*: Random, encircle, and road indicate different telemetry study designs. *κ* is the concentration parameter of the von Mises distribution and controls the amount of azimuthal uncertainty; larger values indicate higher precision. We use *κ*=100 as moderate uncertainty and a *κ*=25 for high uncertainty. *n*_*θ*_ indicates the number of observer locations used for each spatial animal location. $n_{\boldsymbol {\hat {\mu }}}$ is the number of animal spatial location estimates that were appropriately estimated; we simulated a total of 600 locations per scenario. *d*_0.5_ is the median of the Euclidean distance between the estimated animal location and true location ($d(\hat {\boldsymbol {\mu }},\boldsymbol {\mu })$). Coverage is defined as the number of 95% isopleths that contained the true ***μ*** out of the total $n_{\hat {\boldsymbol {\mu }}}$

### Spatial models for azimuthal data

**Fig. 3:**
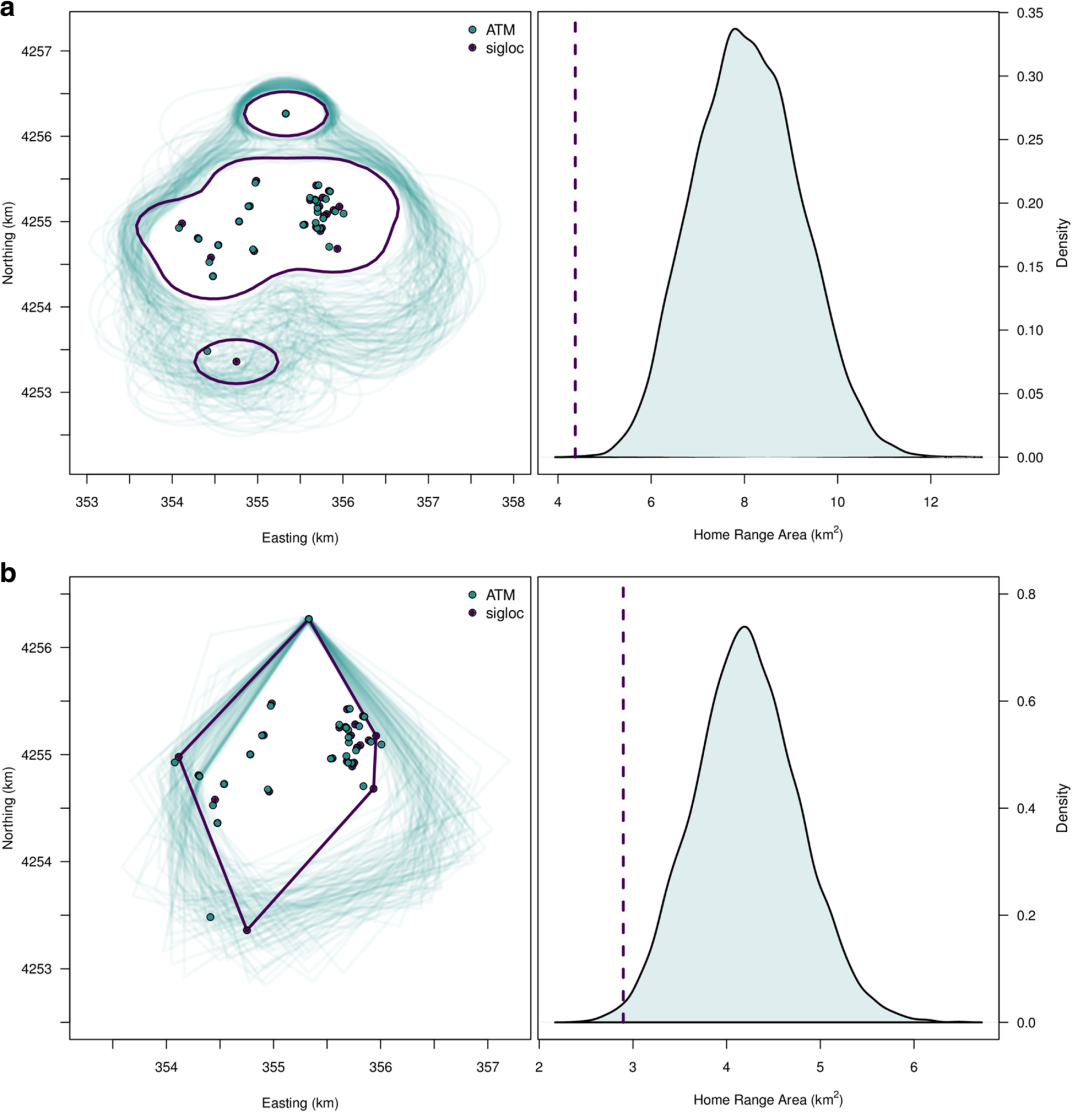
Home range shape and area estimates for an individual Gunnison sage-grouse via kernel estimation (**a**) and convex hull (**b**) where spatial location uncertainty is incorporated via the azimuthal telemetry model (ATM) or ignored using [24] estimation. Right-sided figures depict a histogram of the posterior distribution of the area of the home range when accounting for animal location uncertainty via the ATM; the vertical line is the point estimate of home range area when animal locations are estimated using [24] estimation and location uncertainty is ignored

Regardless of home range estimator, we found the spatial arrangement of the Gunnison sage-grouse home range was often different depending on whether location uncertainty was considered (Fig. 3, Additional file 6). Ignoring location uncertainty often leads to overly small home range area estimates when compared to the estimate obtained when incorporating uncertainty. However, posterior mode home range size can also be smaller when location uncertainty is included (Additional file 6). How location uncertainty in total affects home range size estimates depends largely on the shape of each location posterior distribution, their position relative to each other, and their size relative to the home range area. The contiguity of the kernel density home range was often affected by location uncertainty. Without taking into account location uncertainty, comparing home range area estimates across individuals could lead to highly biased inferences.

**Fig. 4:**
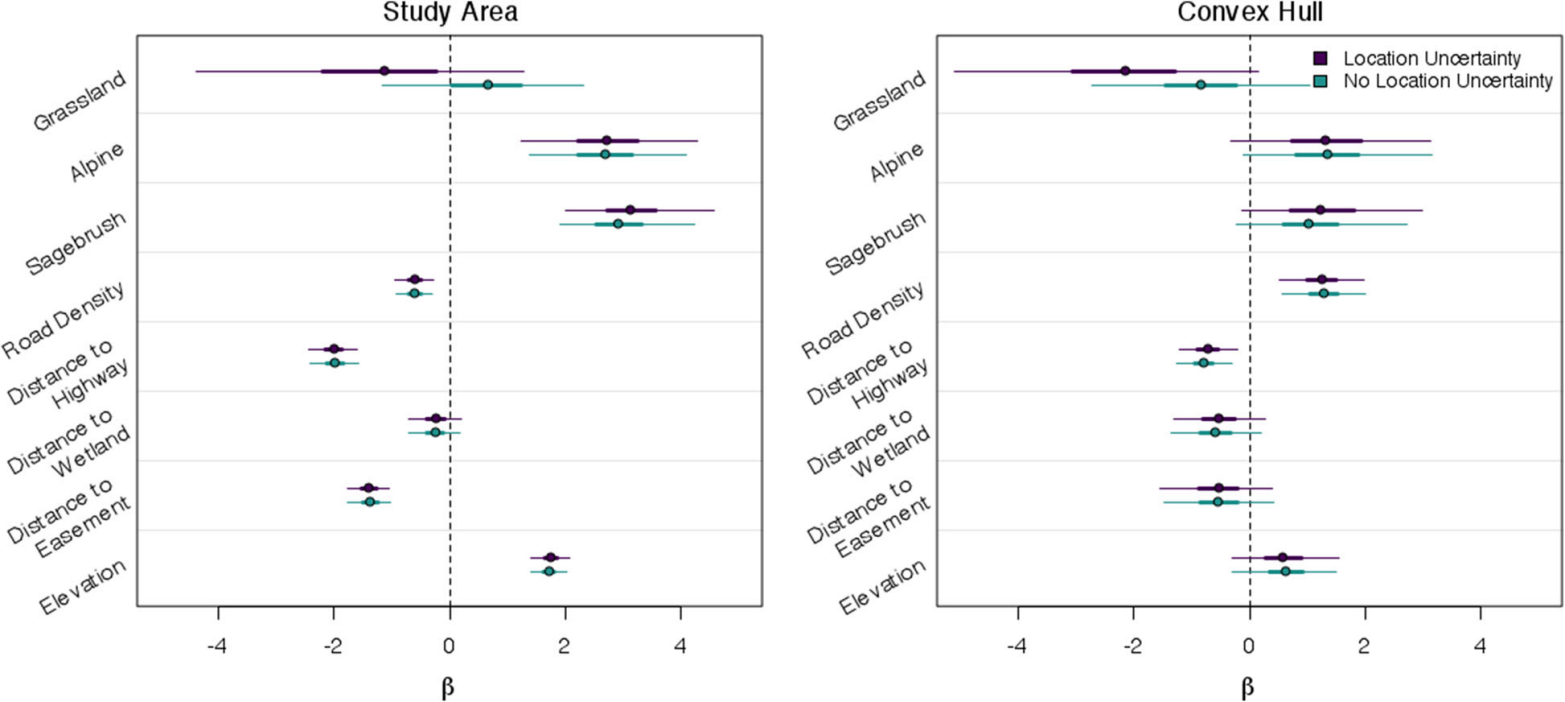
Resource selection coefficients for an individual Gunnison sage-grouse, in which location uncertainty is appropriately propagated and when it is ignored. Points represent posterior medians, thick lines are 50% credible intervals and thin lines represent 95% credible intervals. Evidence for selection or avoidance beyond the availability of a resource is supported by increasing positive or negative probability density, respectively

**Fig. 5:**
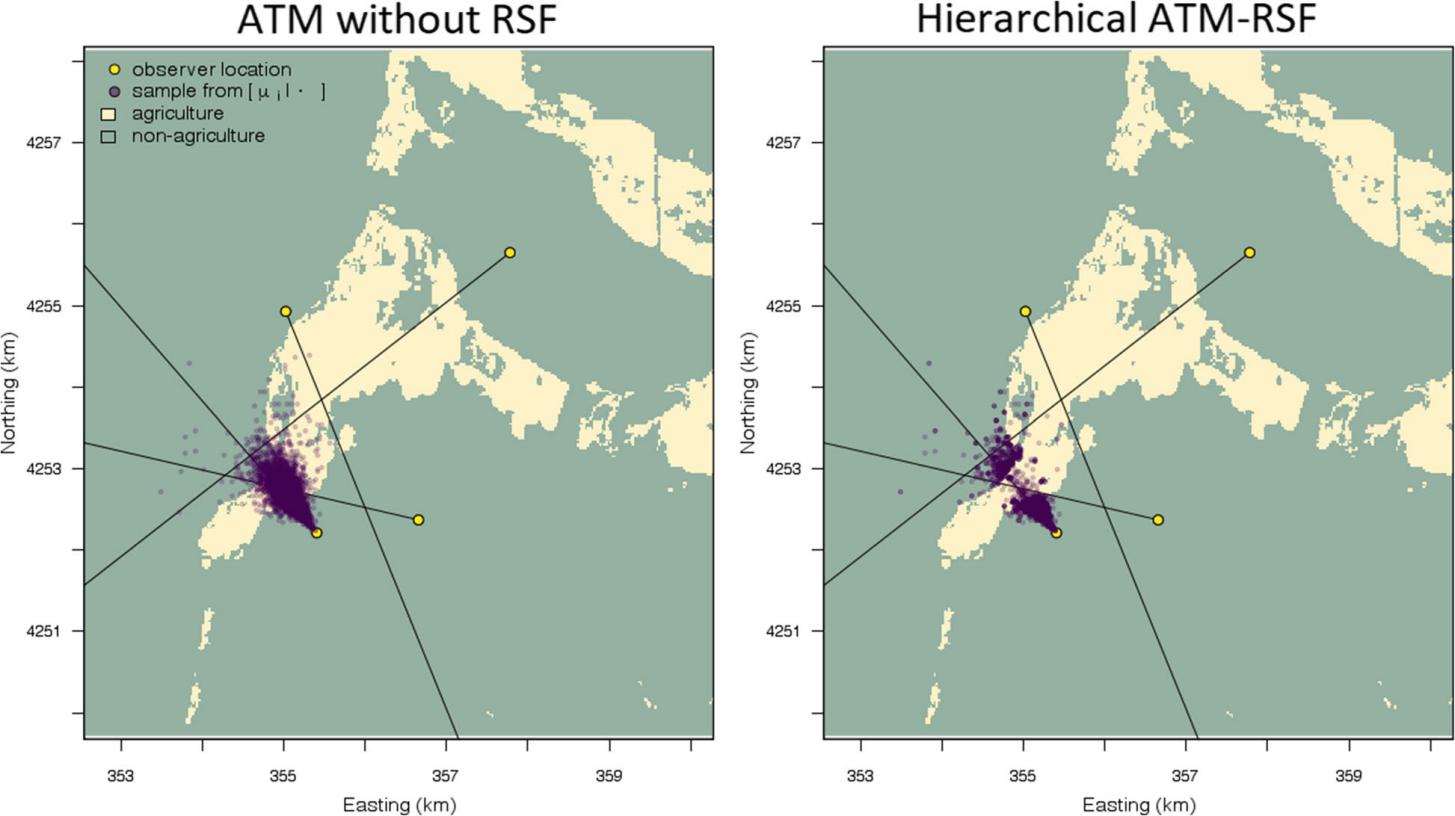
Posterior distribution location samples of a Gunnison sage-grouse location from the ATM only (left subplot) and when there is feedback from a resource selection function (RSF) model to the ATM (right subplot; ATM-RSF). Notice that the RSF can improve location estimation by using the selection coefficients to help inform where the sage-grouse likely was and was not

Resource selection inference depended on how we measured resource availability and whether we included location uncertainty (Fig. 4, Additional file 7: Figures S1-S5). For example, road density was negatively selected at the study area region, but is slightly positively selected at the home range (Fig. 4). Additionally, elevation was positively selected at the study area region, but was selected in proportion to availability (i.e., 95% credible interval includes zero) at the home range level. We found that properly accounting for location uncertainty does not always increase parameter uncertainty (Fig. 4, Additional file 7: Figures S1-S5). Across individuals, we found the categorical vegetation variables were most affected by incorporating location uncertainty, such that including location uncertainty shifted the probability density more negative, even changing the inference and interpretation of the amount of evidence for selection of grasslands to avoidance of grasslands under the study area availability definition. The continuous variables were largely not affected when including location uncertainty, likely due to small location uncertainty relative to the adjacent spatial variability in covariate values. Finally, a considerable advantage of the hierarchical ATM-RSF model is that selection coefficients can inform the location estimation to where individuals were and were not likely to be on the landscape, thus reducing location uncertainty (Fig. 5).

**Fig. 6:**
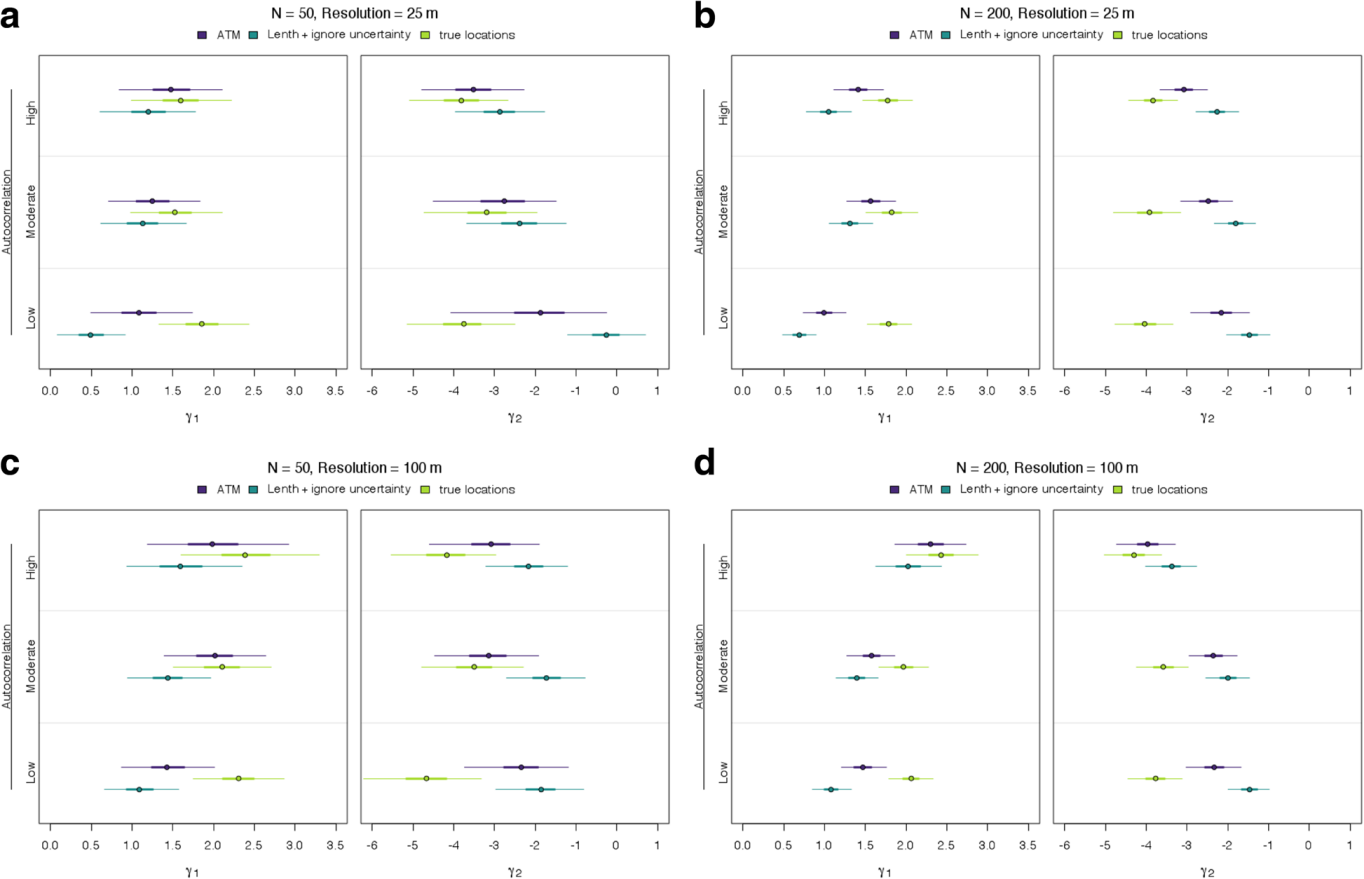
Simulation comparing regression coefficient point estimates from a resource selection function analysis that incorporates location uncertainty (i.e., azimuthal telemetry model (ATM)), locations estimated using Lenth’s (1981) maximum likelihood estimate and ignores location uncertainty, and when true spatial location values are known with complete certainty. Coefficient point estimates correspond to a continuous and categorical variable (*γ*_1_,*γ*_2_, respectively) under low to high autocorrelation. Thick lines are 50% credible intervals and thin lines are 95% credible intervals. The top row (**a**, **b**) correspond to using covariates simulated at a high spatial resolution (25 m) and the bottom row (**c**, **d**) correspond to using low spatial resolution covariates (100 m). The columns differ in the size of the simulated dataset: 50 or 200 locations

From our RSF simulation, we found that differences in regression coefficients among approaches increased as spatial autocorrelation in the covariate value decreased (i.e., higher spatial heterogeneity; Fig. 6). This was the case for both sample sizes and spatial resolutions, however, there was much greater uncertainty with datasets of 50 locations, compared to that of 200. Under all conditions, accounting for location uncertainty results in intervals overlapping the credible interval based on true locations to a higher degree compared to ignoring location uncertainty (Fig. 6). The difference between the ATM-RSF coefficients and those when an RSF model is fit with the known locations is because the ATM does not necessarily estimate locations with the highest posterior density centered on the true location (with high uncertainty in *κ*; Table 1). While we found that incorporating location uncertainty improves our inference about RSF regression coefficients, compared to ignoring location uncertainty, further improvement could be gained by decreasing our azimuthal uncertainty (*κ*) or increasing our certainty in animal locations by taking many more azimuths (Table 1) or requiring animals always be encircled. Finally, we found little difference among coefficients due to the spatial resolution of covariates (25 m vs. 100 m); the most pronounced change was that covariates with high spatial autocorrelation and a lower resolution (100 m) led to similar coefficient estimates regardless of location uncertainty compared to those with high resolution covariates (25 m; only at the high sample size of *N* = 200).

## Discussion

Our model developments have important implications for interpreting historical radio-telemetry data analyses and to study designs for future research projects. While state-of-the-art tracking technologies (e.g., GPS) are increasingly used, animal telemetry via VHF radio is still widely used and will likely continue due to its lower cost and miniaturization [8]. The development of the ATM addresses several complicating factors when dealing with azimuthal data. Foremost is that our model appropriately characterizes azimuthal telemetry uncertainty and allows the uncertainty to synthetically be propagated into spatial models. Appropriately accounting for uncertainties in ecological inference is needed to ensure appropriate inference ([21, 38]; Figs. 3, 4, and 6). The ATM illustrates that the magnitude and shape of location uncertainty from azimuthal telemetry data is complex and highly variable. Previous methods have led to over confidence in the precision of animal locations, the certainty in resource selection, and the size of home ranges.

A current condition of the ATM is that the researcher is required to set a maximum distance in which an animal may be reliably detected. However, alternative prior specifications (e.g., multivariate Gaussian) can be used, but the computational benefits of the Uniform prior imply that posterior distributions are more quickly and fully explored, thus facilitating algorithm convergence. To set the maximum distance, we suggest knowledge of the study area and experience of the researcher should be employed, because the distance will largely depend on the terrain complexity and the behavior of the animal. Field trials can be conducted as part of the study, where a transmitter is placed in known locations and observers attempt to detect the radio signal at increasing distances. Field trials could even be conducted jointly while sampling animals, without the knowledge of the observer, thereby efficiently collecting data on known locations to be used as information for modeling. It should be clear though, the prior has limited influence when typical radio-telemetry data are collected, such that three or more azimuths are taken in a way that they all intersect. The prior will be more important with only 1-2 azimuths and more so if the two azimuths do not intersect. In these cases, the researcher should consider the absolute maximum distance the animal could likely be from the observer, given the surrounding terrain complexity. The maximum distance may likely be different for every location an azimuth is taken. For example, this distance will be quite different if locating a ring-tailed cat (*Bassariscus astutus*) in a desert canyon or flat. The location uncertainty will also depend on how *κ* is being modeled. Because one azimuth provides very little information for estimating *κ*, sharing information about *κ* across spatial locations will help determine how narrow or wide the uncertainty should be around the single azimuth.

Modeling *κ* is a major benefit of the ATM, because it overcomes the issue of limited experimental field trials by allowing telemetry uncertainty to be directly modeled, therefore accounting for telemetry uncertainty in location estimates. Researchers may allow for heterogeneity in *κ* across multi-dimensions, such as across observers, individual animals, spatial or temporal regions, or specific spatial covariates. If the goal is to minimize location uncertainty, we found that it is prudent to encircle the animal, as well as obtain more than three azimuths (Fig. 1d, Table 1, Additional file 2: Figure S3). However, the optimal study design will ultimately depend on the questions being considered (e.g., home range vs. RSF study); researchers can pair the ATM with spatial models to identify optimal study designs that minimize logistical costs and maximizing model performance, something that was not previously possible.

Throughout, we have considered situations where an animal is at a specified location, and our goal is to estimate the animal location via azimuths from known observer locations. Of course animals do not always stay in one place for long, and thus it is important to consider the total amount of time it takes to get the first and last azimuth. Ideally, the amount of time will be short, but this time depends entirely on the species being studied and the season and time of day. If an animal does move during the time period azimuths are taken, it is likely several azimuths will poorly intersect each other, such that the location uncertainty will be fairly large, which is appropriate. A future extension of the ATM could incorporate an animal movement process linked to the amount of time taken in between azimuths, thus properly accounting for this issue. Researchers should be especially concerned of potential bias due to animal movement in situations when only two azimuths are taken and a long time has elapsed between them. Movement is of less concern with only one azimuth and many azimuths will likely help determine whether an animal is moving.

Another concern in radio-telemetry is the issue of signal bounce, in which the radio signal bounces off geographic features, obscuring the direction to the animal. The ATM could be used to model this effect by including terrain complexity as a covariate for *κ*, as long as these azimuths were included as data. It is common to exclude azimuths that may be due to signal bounce. An alternative approach would be to extend the ATM akin to the M-estimators proposed by Length (1981) to identify and remove outliers.

We found the affects of location uncertainty on ecological inference are not straightforward. Our RSF investigation demonstrated how the affect of location uncertainty on parameter estimates depends on the definition of availability [39], whether covariates were categorical or continuous, and the degree of spatial autocorrelation in the covariate. Our simulation clarified that incorporating location uncertainty helps reduce bias in RSF coefficients across all levels of covariate spatial autocorrelation. Previous RSF studies that used azimuthal data and ignored the location uncertainty should be viewed cautiously. Furthermore, our home range results suggest that previous studies that ignored location uncertainty could have been conservative in their estimate of home range areas; ignoring location uncertainty can strongly affect the shape and size of home range estimates.

The ATM may be useful beyond the contexts we have considered. For example, the ATM may be applied to directional frequency analysis and recording sonobuoys of recorded azimuths to whale vocalizations or use azimuths to estimate buoy drift [40]. Locating animals by vocalization direction in a spatial-capture recapture framework may also be a potential utility of the ATM [41]. More generally, the ATM may be useful for acoustic vector sensor data to identify the location of noise sources [42].

## Conclusion

Methodology using azimuthal data has received much less attention than satellite-based telemetry technologies, despite it being a common source of data for small and volant wild animals. More so, it was the sole type of spatial data for wildlife telemetry studies for many decades. We found previous methods using azimuthal data likely led to poor inference, due to disregarding data and ignoring location uncertainty. Specifically, RSF coefficients could be biased and home ranges overly conservative. We also found location estimation and uncertainty is improved using the ATM framework, which can be used to model location uncertainty and has not been possible previously. Most importantly, the ATM can be integrated with spatial ecological models to account for location uncertainty and thus reduce potential biases. Furthermore, the ATM provides considerable flexibility in the design of radio-telemetry studies because it can estimate animal locations from any number of intersecting or non-intersecting azimuths. Future radio-telemetry studies should use the ATM to consider design tradeoffs in an optimal design framework.

## Additional files

Additional file 1 Background information on the Gunnison sage-grouse and telemetry data collected. (PDF 160 kb)

Additional file 2 Visual representation of the von Mises distribution concentration parameter, an animated sequence of observer locations encircling a radio-tagged animal, and estimated distances between observers and Gunnison sage-grouse. (PDF 857 kb)

Additional file 3 Full conditional distributions and Markov chain Monte Carlo algorithm for estimating parameters, and simulation algorithms for the different radio-telemetry study designs. (PDF 301 kb)

Additional file 4 R code to simulate azimuthal data under different study designs and fit the azimuthal telemetry model. (ZIP 50 kb)

Additional file 5 Details of the resource selection analysis simulation. (PDF 1106 kb)

Additional file 6 Estimated home ranges from different individual Gunnison sage-grouse. (PDF 273 kb)

Additional file 7 Estimated resource selection coefficients from different individual Gunnison sage-grouse. (PDF 9042 kb)
