# Supplementary material for: Accounting for location uncertainty in azimuthal telemetry data improves ecological inference
Source: Mov Ecol. 2018 Jul 25;6:14. doi: 10.1186/s40462-018-0129-1 (PMC6058391; doi:10.1186/s40462-018-0129-1)
Supplement: Supplementary file 1 — Background information on the Gunnison sage-grouse and telemetry data collected. (PDF 160 kb) [file 40462_2018_129_MOESM1_ESM.pdf]

Appendix 1

March 3, 2018

Gerber, B. D., M. B. Hooten, C. P. Peck, M. B. Rice, J. H. Gammonley, A. D. Apa, and A. J. Davis. 2018. Accounting for location uncertainty in azimuthal telemetry data improves ecological inference.

Gunnison Sage-grouse

Background

Gunnison sage-grouse (*Centrocercus minimus*; hereafter GUSG) are a federally threatened species (U.S. Fish and Wildlife Service 2014), recently having been distinguished from the closely related Greater sage-grouse (*C. urophasianus*; Young et al. 2000). GUSG have distinct genetic (Oyler-McCance et al. 1999), morphological, and behavioral (Young et al. 1994, 2000) differences from greater sage-grouse (Hupp and Braun 1991). The current distribution of GUSG is also distinct from that of the Greater sage-grouse and has declined to an approximate 10% of its historical distribution (Schroeder et al. 2004). The current population of GUSG is limited to seven disjunct populations in southwest Colorado and one in southeastern Utah (Schroeder et al. 2004). The majority of individuals ($\approx$ 85-90%) of the species exist in a single population within Colorado's Gunnison Basin, including the counties Gunnison and Saguache (GSRSC 2005).

The Gunnison Basin is a mosaic of landcover types and landowners. The area is approximately 2,000 km^2 ranging in elevation from 2,300 to 2,900 m; the Gunnison Basin consists of agriculture and wetlands along low-lying areas, dominated sagebrush (*Artemisia* sp.) and grasslands in the mid-elevations and aspen and conifer forest in the higher elevation mountain areas. Much of the Gunnison Basin is managed by the Bureau of Land Management.

Telemetry Study

Starting in 2004, Colorado Parks and Wildlife initiated a detailed study to better understand demographic and spatial drivers of the GUSG. Between 2004 and 2010 individual GUSG were captured from March to May using spot-lighting techniques (Wakkinen et al. 1992). Individuals were fit with a 16-17 g necklace style VHF ("very high frequency") radio transmitter ($\leq$ 2% of average GUSG body weight; model A4050 by Advanced Telemetry systems or model R12B by Holohil Systems, Ltd.). Each bird was also fit with a uniquely-numbered leg band (National Band and Tag Company). Trapping and handling protocols were approved by the Colorado Parks and Wildlife Animal Care and Use Committee (permit # 02-2005).

Radio-marked individuals were relocated on the ground using hand held Yagi antennas once every 1-3 days, beginning from the date of capture through September. The telemetry study design varied across the years and spatial locations of the birds. Observers were trained to maximize the accuracy of azimuths while considering constraints, such as private property and field conditions. In total, 38,212 azimuths were collected to estimate 9,476 location of GUSG. The number of unique observers varied from 1 to 10, depending on the year. Across the entire study, the number of azimuths recorded per spatial location of GUSG varied from 2 to 12, but was most commonly 4 (Figure 1). Observers most often took azimuths to radio-tagged birds that were $\leq$ 100 m away, but could be as far as 1 km (see ESM 6); these results were obtained by fitting the azimuthal telemetry model (ATM) to the GUSG data from 2005-2010 and calculating the distance from each observer to the highest probable location (i.e., posterior mode).

Resource Selection Function

We included covariates in our resource selection function (RSF) based on a previous analysis of the GUSG data (Rice et al. 2017). All covariates were aligned and set to a 25m x 25m resolution. The vegetation layer was constructed in 2005 from landsat imagery as part of the Colorado Vegetation Classification Project administered by Colorado Parks and Wildlife in collaboration with the Bureau of Land Management and the U.S.D.A. Forest Service. Vegetation classification was simplified to eight categories: forest, sagebrush, bare ground, riparian, grassland, agriculture, and water. See Rice et al. 2017 for details. For our RSF analysis, six individual grouse were analyzed. Some grouse did not encounter all vegetation categories and thus we could not estimate these selection coefficients; estimable coefficients are are presented in Figure 3 and Supplementary Material Appendix 3.

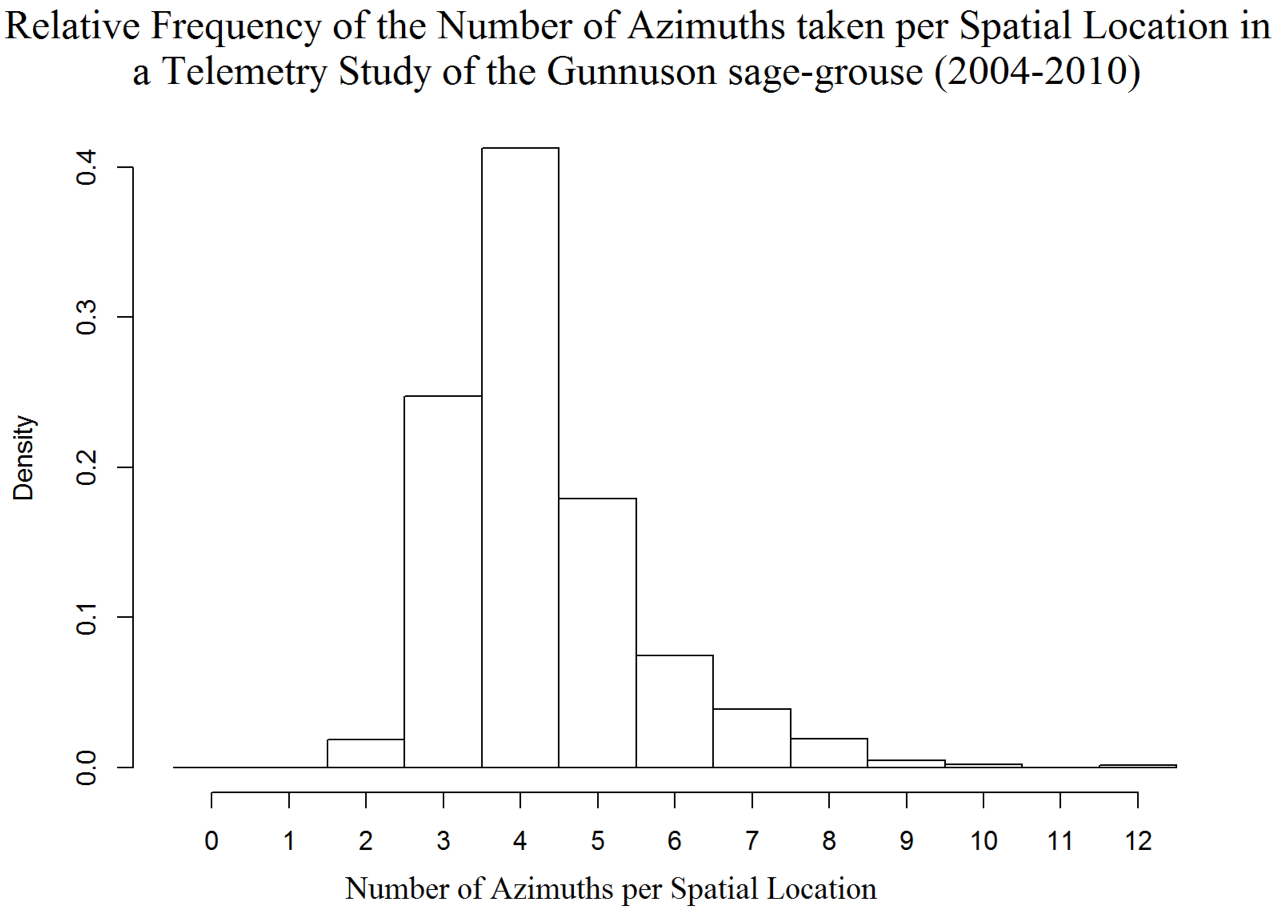

Figure 1: Frequency distribution of the number of azimuths taken to estimate the location of Gunnison sage-grouse.

Literature Cited

Gunnison Sage-grouse Rangewide Steering Committee (GSRSC). (2005). Gunnison sage-grouse rangewide conservation plan. Colorado Division of Wildlife, Denver, CO.

Hupp, J. W., & Braun, C. E. 1991. Geographic variation among sage grouse in Colorado. The Wilson Bulletin **103**:255-261.

OylerMcCance, S. J., Kahn, N. W., Burnham, K. P., Braun, C. E., & Quinn, T. W. 1999. A population genetic comparison of largeand smallbodied sage grouse in Colorado using microsatellite and mitochondrial DNA markers. Molecular Ecology **8**:1457-1465.

Rice, M., Apa, A.D., and Wiechman L. 2017. The importance of seasonal resource selection when managing a threatened species: targeting conservation actions within critical habitat designations for the Gunnison sage-grouse. Wildlife Biology.

Schroeder, M.A., Aldridge, C. L., Apa, A. D., Bohne, J. R., Braun, C.E., Bunnell, S.D., Connelly, J.W., Deibert, P.A., Gardner, S.C., Hilliard, M.A., Kobriger, G.D., McAdam, S.M., McCArthy, C.W., McCarthy, J.J., Mitchell, D.L., Rickerson, E.V., and Stiver, S.J. 2004. Distribution of sage-grouse in North America. The Condor **106**:363-376.

Young, J. R., Hupp, J. W., Bradbury, J. W., & Braun, C. E. 1994. Phenotypic divergence of secondary sexual traits among sage grouse, Centrocercus urophasianus, populations. Animal behaviour **47**:1353-1362.

Young, J. R., Braun, C. E., Oyler-McCance, S. J., Hupp, J. W., & Quinn, T. W. 2000. A new species of sage-grouse (Phasianidae: Centrocercus) from southwestern Colorado. The Wilson Bulletin **112**:445-453.

U.S. Fish and Wildlife Service (2014) Endangered and Threatened Wildlife and Plants; Designation of Critical Habitat for Gunnison Sage-grouse. 50 CFR Part 17; Federal Register **79**:69312-69363.

Wakkinen, W.L., Reese, K.P., Connelly, J.W., and Fischer, R.A. 1992. An improved spotlighting technique for capturing sage-grouse. Wildlife Society Bulletin **20**:425-426.
