# Supplementary material for: Accounting for location uncertainty in azimuthal telemetry data improves ecological inference
Source: Mov Ecol. 2018 Jul 25;6:14. doi: 10.1186/s40462-018-0129-1 (PMC6058391; doi:10.1186/s40462-018-0129-1)
Supplement: Supplementary file 2 — Visual representation of the von Mises distribution concentration parameter, an animated sequence of observer locations encircling a radio-tagged animal, and estimated distances between observers and Gunnison sage-grouse. (PDF 857 kb) [file 40462_2018_129_MOESM2_ESM.pdf]

Appendix 2

March 3, 2018

Gerber, B. D., M. B. Hooten, C. P. Peck, M. B. Rice, J. H. Gammonley, A. D. Apa, and A. J. Davis. 2018. Accounting for location uncertainty in azimuthal telemetry data improves ecological inference.

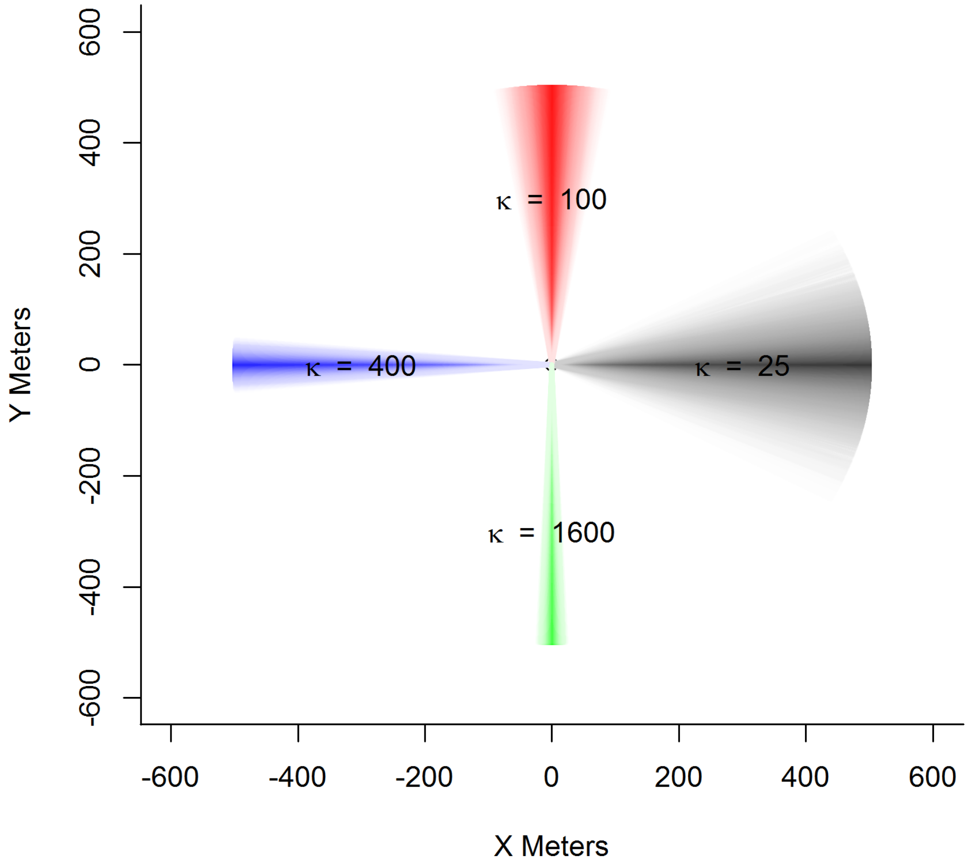

Figure 1. Visual depiction of azimuth uncertainty described by the von Mises distribution concentration parameter, κ.

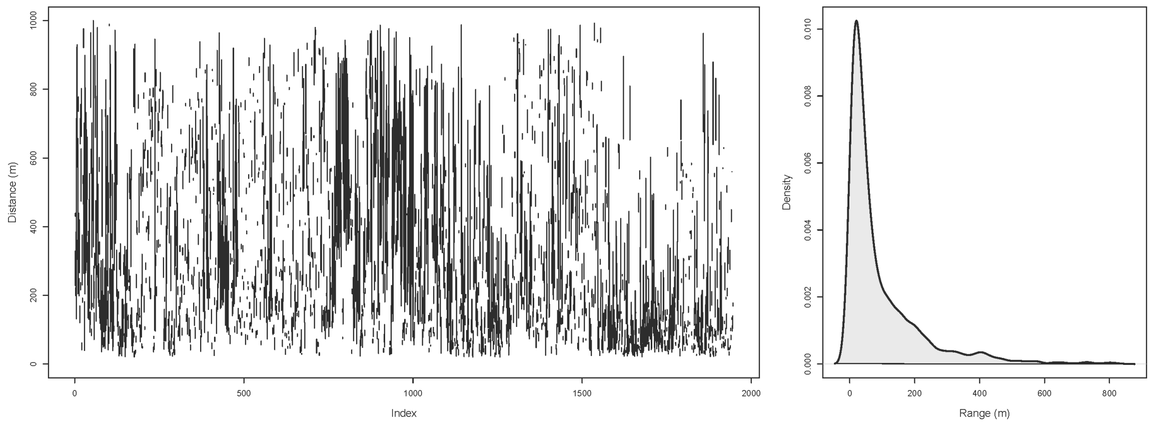

(a) Three azimuths per location.

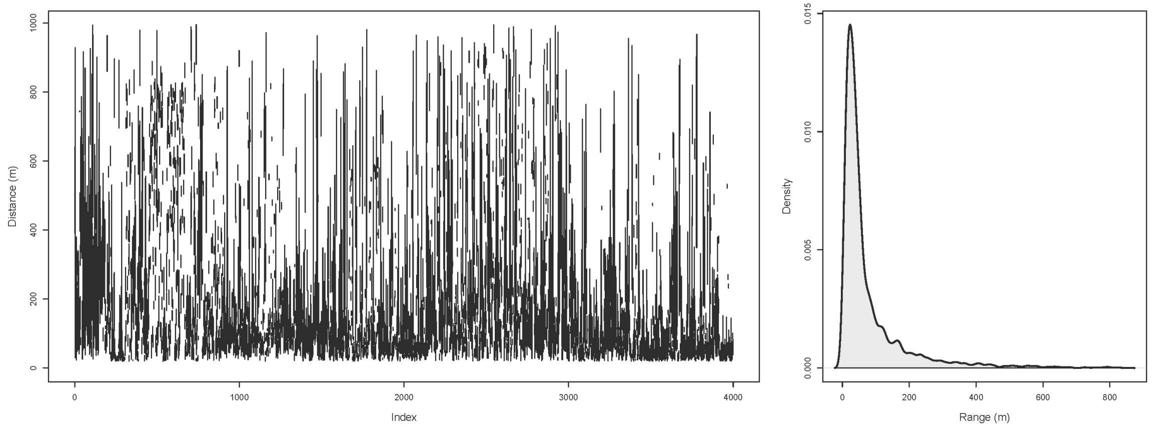

(b) Four azimuths per location.

Figure 2. Ranges of estimated distances between observers and Gunnison sage-grouse from relocations with three or four azimuths along with the marginal density of all ranges.

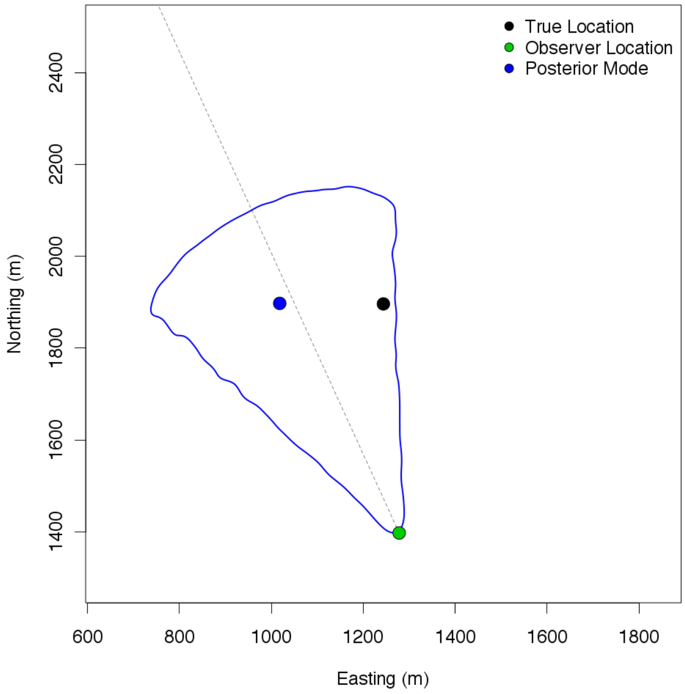

(a)

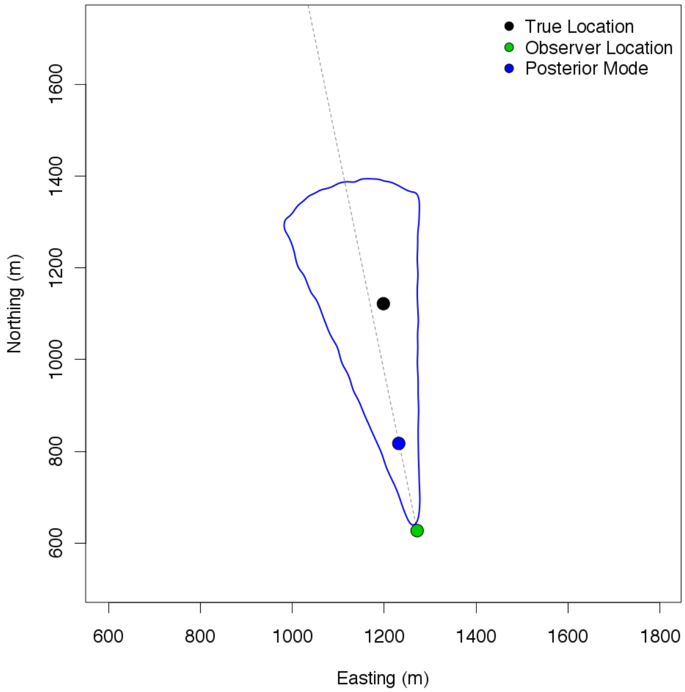

(b)

Figure 3. Animated sequence of observer locations encircling a radio-tagged animal. Azimuths (dotted lines) are made with uncertainty of $\kappa = 25$ (a) and $\kappa = 100$ (b). The blue polygon is the 95% posterior probability contour of the estimated spatial location from the azimthual telemetry model.
