# Supplementary material for: Accounting for location uncertainty in azimuthal telemetry data improves ecological inference
Source: Mov Ecol. 2018 Jul 25;6:14. doi: 10.1186/s40462-018-0129-1 (PMC6058391; doi:10.1186/s40462-018-0129-1)
Supplement: Supplementary file 3 — Full conditional distributions and Markov chain Monte Carlo algorithm for estimating parameters, and simulation algorithms for the different radio-telemetry study designs. (PDF 301 kb) [file 40462_2018_129_MOESM3_ESM.pdf]

Appendix 3

March 3, 2018

Gerber, B. D., M. B. Hooten, C. P. Peck, M. B. Rice, J. H. Gammonley, A. D. Apa, and A. J. Davis. 2018. Accounting for location uncertainty in azimuthal telemetry data improves ecological inference.

A Full-conditional distributions and Markov chain Monte Carlo algorithm for parameter estimation

The proposed azimuthal telemetry models were fit using a Markov chain Monte Carlo (MCMC) algorithm implemented in the R computing environment.

A.1 Observer model from "Azimuthal Telemetry Model (ATM)"

Suppose that multiple individuals ($l = 1, \ldots, L$) are fixed with a radio-transmitter within a study region and are subsequently relocated using radio-telemetry on $i = 1, \ldots, n_l$ days. For each relocation attempt, an observer records a set of azimuths (θ_{lij}; $j = 1, \ldots, J_{li}$) at known locations $\boldsymbol{s}_{lij} = (s_{1lij}, s_{2lij})'$ to estimate the true transmitter spatial location, $\boldsymbol{\mu}_{li} = (\mu_{1li}, \mu_{2li})'$.

Model Statement[1]

$$\begin{aligned}
\theta_{lij} &\sim \text{vonMises}\left(\tilde{\theta}_{lij}, \kappa_{li}\right) \\
\tilde{\theta}_{lij} &= \tan^{-1}\left(\frac{\mu_{2li} - s_{2lij}}{\mu_{1li} - s_{1lij}}\right) \\
\boldsymbol{\mu}_{li} &\sim \text{U}\left(\mathcal{S}_{li}\right) \\
\mathcal{S}_{li} &= \bigcup_{j=1}^{J_{li}} \left\{(x, y) \,|\, (x - s_{1lij})^2 + (y - s_{2lij})^2 \leq r^2\right\} \\
\log\left(\kappa_{li}\right) &\sim \text{N}\left(\beta_0 + \beta_1 \mathbf{1}_{\{\texttt{B}\}}, \sigma^2_\kappa\right) \\
\beta_0 &\sim \text{N}\left(\mu_\beta, \sigma^2_\beta\right) \\
\beta_1 &\sim \text{N}\left(\mu_\beta, \sigma^2_\beta\right) \\
\sigma_\kappa &\sim \text{Inv-gamma}\left(\alpha_\sigma, \beta_\sigma\right)
\end{aligned}$$

[1]Assuming 2 observers (A,B) with 1 observer per location i

Full-Conditional Distributions

$$[\boldsymbol{\mu}_{li} \,|\, \cdot\,] \propto \left\{ \prod_{j=1}^{J_{li}} [\theta_{lij} \,|\, \boldsymbol{\mu}_{li}, \kappa_{li}] \right\} [\boldsymbol{\mu}_{li}]$$

$$[\kappa_{li} \,|\, \cdot\,] \propto \left\{ \prod_{j=1}^{J_{li}} [\theta_{lij} \,|\, \boldsymbol{\mu}_{li}, \kappa_{li}] \right\} [\,\log(\kappa_{li}) \,|\, \boldsymbol{\beta}, \sigma_\kappa]$$

$$[\beta_0 \,|\, \cdot\,] \propto \left\{ \prod_{l=1}^{L} \prod_{i=1}^{n_l} [\,\log(\kappa_{li}) \,|\, \boldsymbol{\beta}, \sigma_\kappa] \right\} [\beta_0]$$

$$[\beta_1 \,|\, \cdot\,] \propto \left\{ \prod_{l=1}^{L} \prod_{i=1}^{n_l} [\,\log(\kappa_{li}) \,|\, \boldsymbol{\beta}, \sigma_\kappa] \right\} [\beta_1]$$

$$[\sigma_\kappa \,|\, \cdot\,] \propto \left\{ \prod_{l=1}^{L} \prod_{i=1}^{n_l} [\,\log(\kappa_{li}) \,|\, \boldsymbol{\beta}, \sigma_\kappa] \right\} [\sigma_\kappa]$$

MCMC Algorithm for Parameter Estimation

To estimate the parameters above, an MCMC algorithm was implemented as follows:

1. Define initial values for the model parameters: $\kappa_{li}^{(0)}$, $\beta_0^{(0)}$, $\beta_1^{(0)}$, and $\sigma_\kappa^{(0)}$. Define values for hyperparameters: r, μ_β, σ_β^2, α_σ, and β_σ.

2. Generate grid of points on $\mathcal{S}_{li}$ and compute $\tilde{\theta}_{lij}$ for each grid point. Set $k = 1$.

3. Direct approximation of $[\boldsymbol{\mu}_{li} \,|\, \cdot]$:

   (a) Obtain $\boldsymbol{\mu}_{li}^{(k)}$ by taking a random draw from the set of grid points $m = 1, \ldots, M$ where the probability of each grid point m is defined as:

$$p_m = \frac{\prod_{j=1}^{J_{li}} \left[\theta_{lij} \,|\, \boldsymbol{\mu}_m, \kappa_{li}^{(k-1)}\right]}{\sum_{m=1}^{M} \prod_{j=1}^{J_{li}} \left[\theta_{lij} \,|\, \boldsymbol{\mu}_m, \kappa_{li}^{(k-1)}\right]}.$$

4. Metropolis-Hastings step for $[\kappa_{li} \,|\, \cdot]$:

   (a) Sample $\kappa_{li}^{(*)}$ from the proposal distribution $\left[\kappa_{li}^{(*)} \,|\, \kappa_{li}^{(k-1)}\right] = \mathrm{N}_{(0,\infty)}\left(\kappa_{li}^{(k-1)}, \tau_\kappa^2\right)$ where τ_κ^2 is a tuning parameter and $\mathrm{N}_{(0,\infty)}(\,\cdot\,)$ represents a truncated normal distribution with support $(0, \infty)$.

   (b) Compute the ratio of densities,

$$a = \frac{\left\{\prod_{j=1}^{J_{li}} \left[\theta_{lij} \,|\, \boldsymbol{\mu}_{li}^{(k)}, \kappa_{li}^{(*)}\right]\right\} \left[\log\left(\kappa_{li}^{(*)}\right) \,|\, \beta_0^{(k-1)}, \beta_1^{(k-1)}, \sigma_\kappa^{(k-1)}\right]}{\left\{\prod_{j=1}^{J_{li}} \left[\theta_{lij} \,|\, \boldsymbol{\mu}_{li}^{(k)}, \kappa_{li}^{(k-1)}\right]\right\} \left[\log\left(\kappa_{li}^{(k-1)}\right) \,|\, \beta_0^{(k-1)}, \beta_1^{(k-1)}, \sigma_\kappa^{(k-1)}\right]} \cdot \frac{\left[\kappa_{li}^{(k-1)} \,|\, \kappa_{li}^{(*)}\right]}{\left[\kappa_{li}^{(*)} \,|\, \kappa_{li}^{(k-1)}\right]}$$

   (c) Set

$$\kappa_{li}^{(k)} = \begin{cases} \kappa_{li}^{(*)} & \text{with probability } \min(a, 1) \\ \kappa_{li}^{(k-1)} & \text{otherwise} \end{cases}$$

5. Metropolis step for $[\beta_0 \,|\, \cdot]$:

   (a) Sample $\beta_0^{(*)}$ from the proposal distribution $\left[\beta_0^{(*)} \,|\, \beta_0^{(k-1)}\right] = \mathrm{N}\left(\beta_0^{(k-1)}, \tau_{\beta_0}^2\right)$ where $\tau_{\beta_0}^2$ is a tuning parameter.

   (b) Compute the ratio of densities,

$$a = \frac{\left\{\prod_{l=1}^{L} \prod_{i=1}^{n_l} \left[\log\left(\kappa_{li}^{(k)}\right) \,|\, \beta_0^{(*)}, \beta_1^{(k-1)}, \sigma_\kappa^{(k-1)}\right]\right\} \left[\beta_0^{(*)} \,|\, \mu_\beta, \sigma_\beta^2\right]}{\left\{\prod_{l=1}^{L} \prod_{i=1}^{n_l} \left[\log\left(\kappa_{li}^{(k)}\right) \,|\, \beta_0^{(k-1)}, \beta_1^{(k-1)}, \sigma_\kappa^{(k-1)}\right]\right\} \left[\beta_0^{(k-1)} \,|\, \mu_\beta, \sigma_\beta^2\right]}$$

   (c) Set

$$\beta_0^{(k)} = \begin{cases} \beta_0^{(*)} & \text{with probability } \min(a, 1) \\ \beta_0^{(k-1)} & \text{otherwise} \end{cases}$$

6. Metropolis step for $[\beta_1 \,|\, \cdot]$:

   (a) Sample $\beta_1^{(*)}$ from the proposal distribution $\left[\beta_1^{(*)} \,|\, \beta_1^{(k-1)}\right] = \mathrm{N}\left(\beta_1^{(k-1)}, \tau_{\beta_1}^2\right)$ where $\tau_{\beta_1}^2$ is a tuning parameter.

(b) Compute the ratio of densities,

$$a = \frac{\left\{\prod_{l=1}^{L}\prod_{i=1}^{n_l}\left[\log\left(\kappa_{li}^{(k)}\right) \mid \beta_0^{(k)}, \beta_1^{(*)}, \sigma_\kappa^{(k-1)}\right]\right\}\left[\beta_1^{(*)} \mid \mu_\beta, \sigma_\beta^2\right]}{\left\{\prod_{l=1}^{L}\prod_{i=1}^{n_l}\left[\log\left(\kappa_{li}^{(k)}\right) \mid \beta_0^{(k)}, \beta_1^{(k-1)}, \sigma_\kappa^{(k-1)}\right]\right\}\left[\beta_1^{(k-1)} \mid \mu_\beta, \sigma_\beta^2\right]}$$

(c) Set

$$\beta_1^{(k)} = \begin{cases}\beta_1^{(*)} & \text{with probability } \min(a,1)\\ \beta_1^{(k-1)} & \text{otherwise}\end{cases}$$

7. Metropolis-Hastings step for $[\sigma_\kappa \mid \cdot]$:

(a) Sample $\sigma_\kappa^{(*)}$ from the proposal distribution $\left[\sigma_\kappa^{(*)} \mid \sigma_\kappa^{(k-1)}\right] = \mathrm{N}_{(0,\infty)}\left(\sigma_\kappa^{(k-1)}, \tau_\sigma^2\right)$ where τ_σ^2 is a tuning parameter and $\mathrm{N}_{(0,\infty)}(\,\cdot\,)$ represents a truncated normal distribution with support $(0,\infty)$

(b) Compute the ratio of densities,

$$a = \frac{\left\{\prod_{l=1}^{L}\prod_{i=1}^{n_l}\left[\log\left(\kappa_{li}^{(k)}\right) \mid \beta_0^{(k)}, \beta_1^{(k)}, \sigma_\kappa^{(*)}\right]\right\}\left[\sigma_\kappa^{(*)} \mid \alpha_\sigma, \beta_\sigma\right]}{\left\{\prod_{l=1}^{L}\prod_{i=1}^{n_l}\left[\log\left(\kappa_{li}^{(k)}\right) \mid \beta_0^{(k)}, \beta_1^{(k)}, \sigma_\kappa^{(k-1)}\right]\right\}\left[\sigma_\kappa^{(k-1)} \mid \alpha_\sigma, \beta_\sigma\right]} \cdot \frac{\left[\sigma_\kappa^{(k-1)} \mid \sigma_\kappa^{(*)}\right]}{\left[\sigma_\kappa^{(*)} \mid \sigma_\kappa^{(k-1)}\right]}$$

(c) Set

$$\sigma_\kappa^{(k)} = \begin{cases}\sigma_\kappa^{(*)} & \text{with probability } \min(a,1)\\ \sigma_\kappa^{(k-1)} & \text{otherwise}\end{cases}$$

8. Save $\boldsymbol{\mu}_{li}^{(k)}$, $\kappa_{li}^{(k)}$, $\beta_0^{(k)}$, $\beta_1^{(k)}$, and $\sigma_\kappa^{(k)}$. Set $k = k+1$ and return to step 3. Iterate algorithm by repeating steps 3–7 until a sufficiently large sample has been obtained from which to approximate the posterior distribution.

A.2 Single κ model from "Simulation"

Suppose that a single individual is fixed with a radio-transmitter within a study region and is subsequently relocated using radio-telemetry on $i = 1, \ldots, n$ days. For each relocation attempt, an observer records a set of azimuths (θ_{ij}; $j = 1, \ldots, J_i$) at known locations $\boldsymbol{s}_{ij} = (s_{1ij}, s_{2ij})'$ to estimate the true transmitter spatial location, $\boldsymbol{\mu}_i = (\mu_{1i}, \mu_{2i})'$.

Model Statement

$$\begin{aligned}
\theta_{ij} &\sim \text{vonMises}\left(\tilde{\theta}_{ij}, \kappa\right)\\
\tilde{\theta}_{ij} &= \tan^{-1}\left(\frac{\mu_{2i} - s_{2ij}}{\mu_{1i} - s_{1ij}}\right)\\
\boldsymbol{\mu}_i &\sim \mathrm{U}\left(\mathcal{S}_i\right)\\
\mathcal{S}_i &= \bigcup_{j=1}^{J_i}\left\{(x,y) \mid (x - s_{1ij})^2 + (y - s_{2ij})^2 \le r^2\right\}\\
\kappa &\sim \mathrm{U}\left(\alpha, \beta\right)
\end{aligned}$$

Full-Conditional Distributions

$$[\boldsymbol{\mu}_i \mid \cdot\,] \propto \prod_{j=1}^{J_i}[\theta_{ij} \mid \boldsymbol{\mu}_i, \kappa][\boldsymbol{\mu}_i]$$

$$[\kappa \mid \cdot\,] \propto \prod_{i=1}^{n}\prod_{j=1}^{J_i}[\theta_{ij} \mid \boldsymbol{\mu}_i, \kappa][\kappa]$$

MCMC Algorithm for Parameter Estimation

To estimate the parameters above, an MCMC algorithm was implemented as follows:

1. Define initial value for von Mises concentration parameter $\kappa^{(0)}$. Define values for hyperparameters: r, α, and β.

2. Generate grid of points on $\mathcal{S}_i$ and compute $\tilde{\theta}_{ij}$ for each grid point. Set $k = 1$.

3. Direct approximation of $[\boldsymbol{\mu}_i \,|\, \cdot]$:

   (a) Obtain $\boldsymbol{\mu}_i^{(k)}$ by taking a random draw from the set of grid points $m = 1, \ldots, M$ where the probability of each grid point m is defined as:

$$p_m = \frac{\prod_{j=1}^{J_i} \left[\theta_{ij} \,|\, \boldsymbol{\mu}_m, \kappa^{(k-1)}\right]}{\sum_{m=1}^{M} \prod_{j=1}^{J_i} \left[\theta_{ij} \,|\, \boldsymbol{\mu}_m, \kappa^{(k-1)}\right]}$$

4. Metropolis-Hastings step for $[\kappa \,|\, \cdot]$:

   (a) Sample $\kappa^{(*)}$ from the proposal distribution $\left[\kappa^{(*)} \,|\, \kappa^{(k-1)}\right] = \text{N}_{[\alpha,\beta]}\left(\kappa^{(k-1)}, \tau_\kappa^2\right)$ where τ_κ^2 is a tuning parameter and $\text{N}_{[\alpha,\beta]}(\,\cdot\,)$ represents a truncated normal distribution with support $[\alpha, \beta]$.

   (b) Compute the ratio of densities,

$$a = \frac{\prod_{i=1}^{n} \prod_{j=1}^{J_i} \left[\theta_{ij} \,|\, \boldsymbol{\mu}_i^{(k)}, \kappa^{(*)}\right]}{\prod_{i=1}^{n} \prod_{j=1}^{J_i} \left[\theta_{ij} \,|\, \boldsymbol{\mu}_i^{(k)}, \kappa^{(k-1)}\right]} \cdot \frac{\left[\kappa^{(k-1)} \,|\, \kappa^{(*)}\right]}{\left[\kappa^{(*)} \,|\, \kappa^{(k-1)}\right]}$$

   (c) Set

$$\kappa^{(k)} = \begin{cases} \kappa^{(*)} & \text{with probability } \min(a, 1) \\ \kappa^{(k-1)} & \text{otherwise} \end{cases}$$

5. Save $\boldsymbol{\mu}_i^{(k)}$ and $\kappa^{(k)}$. Set $k = k + 1$ and return to step 3. Iterate algorithm by repeating steps 3–4 until a sufficiently large sample has been obtained from which to approximate the posterior distribution.

A.3 ATM-RSF model from "Resource Selection Model"

The following details are in addition to those presented in §A.2.

Model Statement

$$[\boldsymbol{\mu}_i \,|\, \boldsymbol{\gamma}] = \frac{\exp\left(\boldsymbol{x}'(\boldsymbol{\mu}_i)\boldsymbol{\gamma}\right)}{\int \exp\left(\boldsymbol{x}'(\boldsymbol{\mu})\boldsymbol{\gamma}\right) d\boldsymbol{\mu}}$$

$$\gamma_h \sim \text{N}\left(\mu_\gamma, \sigma_\gamma^2\right), \text{ for } h = 1, \ldots, 7$$

Full-Conditional Distributions

$$[\gamma_h \,|\, \cdot\,] = \left\{ \prod_{i=1}^{n} \frac{\exp\left(\boldsymbol{x}'(\boldsymbol{\mu}_i)\gamma_h\right)}{\int \exp\left(\boldsymbol{x}'(\boldsymbol{\mu})\boldsymbol{\gamma}\right) d\boldsymbol{\mu}} \right\} [\gamma_h]$$

MCMC Algorithm for Parameter Estimation

To estimate the parameters above, an MCMC algorithm was implemented concurrently with the algorithm presented in §A.2 as follows:

1. Define initial values for the RSF covariates: $\gamma_1^{(0)}, \gamma_2^{(0)}, \ldots, \gamma_7^{(0)}$. Define values for hyperparameters: μ_γ and σ_γ^2. Set $k = 1$.
2. Metropolis step for $[\gamma_1 \mid \cdot]$:
   (a) Sample $\gamma_1^{(*)}$ from the proposal distribution $\left[\gamma_1^{(*)} \mid \gamma_1^{(k-1)}\right] = \mathrm{N}\left(\gamma_1^{(k-1)}, \sigma_{\gamma_1}^2\right)$ where $\sigma_{\gamma_1}^2$ is a tuning parameter.
   (b) Compute the ratio of densities,
   $$a = \frac{\left\{\prod_{i=1}^{n} \frac{\exp\left(\boldsymbol{x}'\left(\boldsymbol{\mu}_i^{(k)}\right)\gamma_1^{(*)}\right)}{\int \exp\left(\boldsymbol{x}'(\boldsymbol{\mu})\boldsymbol{\gamma}^*\right) d\boldsymbol{\mu}}\right\}\left[\gamma_1^{(*)} \mid \mu_\gamma, \sigma_\gamma^2\right]}{\left\{\prod_{i=1}^{n} \frac{\exp\left(\boldsymbol{x}'\left(\boldsymbol{\mu}_i^{(k)}\right)\gamma_1^{(k-1)}\right)}{\int \exp\left(\boldsymbol{x}'(\boldsymbol{\mu})\boldsymbol{\gamma}^{**}\right) d\boldsymbol{\mu}}\right\}\left[\gamma_1^{(k-1)} \mid \mu_\gamma, \sigma_\gamma^2\right]},$$
   where $\boldsymbol{\gamma}^* = \left(\gamma_1^{(*)}, \gamma_2^{(k-1)}, \ldots, \gamma_7^{(k-1)}\right)'$ and $\boldsymbol{\gamma}^{**} = \left(\gamma_1^{(k-1)}, \gamma_2^{(k-1)}, \ldots, \gamma_7^{(k-1)}\right)'$
   (c) Set
   $$\gamma_1^{(k)} = \begin{cases} \gamma_1^{(*)} & \text{with probability } \min(a, 1) \\ \gamma_1^{(k-1)} & \text{otherwise} \end{cases}$$
3. Metropolis step for $[\gamma_2 \mid \cdot]$:
   (a) Sample $\gamma_2^{(*)}$ from the proposal distribution $\left[\gamma_2^{(*)} \mid \gamma_2^{(k-1)}\right] = \mathrm{N}\left(\gamma_2^{(k-1)}, \sigma_{\gamma_2}^2\right)$ where $\sigma_{\gamma_2}^2$ is a tuning parameter.
   (b) Compute the ratio of densities,
   $$a = \frac{\left\{\prod_{i=1}^{n} \frac{\exp\left(\boldsymbol{x}'\left(\boldsymbol{\mu}_i^{(k)}\right)\gamma_2^{(*)}\right)}{\int \exp\left(\boldsymbol{x}'(\boldsymbol{\mu})\boldsymbol{\gamma}^*\right) d\boldsymbol{\mu}}\right\}\left[\gamma_2^{(*)} \mid \mu_\gamma, \sigma_\gamma^2\right]}{\left\{\prod_{i=1}^{n} \frac{\exp\left(\boldsymbol{x}'\left(\boldsymbol{\mu}_i^{(k)}\right)\gamma_2^{(k-1)}\right)}{\int \exp\left(\boldsymbol{x}'(\boldsymbol{\mu})\boldsymbol{\gamma}^{**}\right) d\boldsymbol{\mu}}\right\}\left[\gamma_2^{(k-1)} \mid \mu_\gamma, \sigma_\gamma^2\right]},$$
   where $\boldsymbol{\gamma}^* = \left(\gamma_1^{(k)}, \gamma_2^{(*)}, \gamma_3^{(k-1)}, \ldots, \gamma_7^{(k-1)}\right)$ and $\boldsymbol{\gamma}^{**} = \left(\gamma_1^{(k)}, \gamma_2^{(k-1)}, \gamma_3^{(k-1)}, \ldots, \gamma_7^{(k-1)}\right)$
   (c) Set
   $$\gamma_2^{(k)} = \begin{cases} \gamma_2^{(*)} & \text{with probability } \min(a, 1) \\ \gamma_2^{(k-1)} & \text{otherwise} \end{cases}$$
4. Iterate through remaining γ parameters ($h = 3, \ldots, 7$) using Metropolis steps and continuing the updating pattern given in steps 2–3.
5. Save $\gamma_1^{(k)}, \gamma_2^{(k)}, \ldots, \gamma_7^{(k)}$. Set $k = k + 1$ and return to step 2. Iterate algorithm by repeating steps 2–4 until a sufficiently large sample has been obtained from which to approximate the posterior distribution.

A.4 ATM-HR model from "Home Range"

The 95% kernel density estimate (KDE) isopleth or convex hull of $\boldsymbol{\mu}_i$ ($i = 1, \ldots, n$) is a derived quantity and thus can be computed using $\boldsymbol{\mu}_i^{(k)}$ for each iteration k of the MCMC algorithm. The results presented in Figure 4 are derived quantities from the algorithm presented in §A.2.

B Simulation Algorithms

The following details the steps used to simulate a single location under each specified design using $\kappa = 100$ and 3 observer locations. Similar simulation steps were used for designs with $\kappa = 25$ and 4 bearings. Data used to fit the model given in §A.2 under each design are the observer locations ($\boldsymbol{s}_i = (s_{1i}, s_{2i})'$) and the observed azimuths (θ_i^{adj}).

B.1 Random Design

1. Draw $\mu_1 \sim \text{U}(1000, 2000)$ and $\mu_2 \sim \text{U}(1000, 2000)$
2. Draw $\theta_i \sim \text{U}(-\pi, \pi)$ for $i = 1, \ldots, 3$
3. Draw $d_i \sim f_d$ for $i = 1, \ldots, 3$ where f_d is a doubly truncated exponential distribution (lower = 25, upper = 2000) with rate parameter equal to $\lambda_{\text{MLE}} = 0.0039$ from the non-truncated "empirical" distribution of distances (years 2005–2010). The coordinates of $\boldsymbol{s}_i$ are obtained using the following equations:

$$s_{1i} = \mu_1 + \cos(\theta_i) \cdot d_i$$
$$s_{2i} = \mu_2 + \sin(\theta_i) \cdot d_i$$

4. For each θ_i, draw $\theta_i^{\text{adj}} \sim \text{vonMises}\left(\tilde{\theta} = \theta_i - \pi, \kappa = 100\right)$

B.2 Encircle Design

1. Draw $\mu_1 \sim \text{U}(1000, 2000)$ and $\mu_2 \sim \text{U}(1000, 2000)$
2. Draw $\theta_1 \sim \text{U}(-\pi, \pi)$. For $i = 2, 3$, draw $\theta_i' \sim \text{U}\left(\frac{\pi}{6}, \frac{\pi}{3}\right)$ and add to previous bearing: $\theta_i = \theta_{i-1} + \theta_i'$
3. Draw $d_i \sim f_d$ for $i = 1, \ldots, 3$ where f_d is a doubly truncated exponential distribution (lower = 25, upper = 2000) with rate parameter equal to $\lambda_{\text{MLE}} = 0.0039$ from the non-truncated "empirical" distribution of distances (years 2005–2010). The coordinates of $\boldsymbol{s}_i$ are obtained using the following equations:

$$s_{1i} = \mu_1 + \cos(\theta_i) \cdot d_i$$
$$s_{2i} = \mu_2 + \sin(\theta_i) \cdot d_i$$

4. For each θ_i, draw $\theta_i^{\text{adj}} \sim \text{vonMises}\left(\tilde{\theta} = \theta_i - \pi, \kappa = 100\right)$

B.3 Road Design

1. Draw $\mu_1 \sim \text{U}(1000, 2000)$ and $\mu_2 \sim \text{U}(1000, 2000)$
2. Draw $\theta^{\text{road}} \sim \text{U}(-\pi, \pi)$ and $d^{\text{road}} \sim \text{U}(50, 250)$ (i.e., direction and distance to perpendicular road feature)
3. Given a maximum span of 160°, we obtain the endpoints of the road feature $\boldsymbol{s}_k^{\text{road}}$ ($k = 1, 2$) as follows:

$$s_{11}^{\text{road}} = \mu_1 + \cos\left(\theta^{\text{road}} + \frac{4\pi}{9}\right) \cdot \frac{d^{\text{road}}}{\cos\left(\frac{4\pi}{9}\right)}$$
$$s_{21}^{\text{road}} = \mu_2 + \sin\left(\theta^{\text{road}} + \frac{4\pi}{9}\right) \cdot \frac{d^{\text{road}}}{\cos\left(\frac{4\pi}{9}\right)}$$
$$s_{12}^{\text{road}} = \mu_1 + \cos\left(\theta^{\text{road}} - \frac{4\pi}{9}\right) \cdot \frac{d^{\text{road}}}{\cos\left(\frac{4\pi}{9}\right)}$$
$$s_{22}^{\text{road}} = \mu_2 + \sin\left(\theta^{\text{road}} - \frac{4\pi}{9}\right) \cdot \frac{d^{\text{road}}}{\cos\left(\frac{4\pi}{9}\right)}$$

4. Compute angle between $\boldsymbol{s}_1^{\text{road}}$ and $\boldsymbol{s}_2^{\text{road}}$: $\theta^* = \tan^{-1}\left(\frac{s_{22}^{\text{road}} - s_{21}^{\text{road}}}{s_{12}^{\text{road}} - s_{11}^{\text{road}}}\right)$ and the Euclidean distance between them $d^* = \sqrt{(s_{22}^{\text{road}} - s_{21}^{\text{road}})^2 + (s_{12}^{\text{road}} - s_{11}^{\text{road}})^2}$

5. Split d^* into 3 equal-length segments and sample uniformly on length of each: for $i = 1, \ldots, 3$, draw $d_i \sim \text{U}\left((i-1) \cdot \frac{d^*}{3}, (i) \cdot \frac{d^*}{3}\right)$

6. Obtain observer location points: for $i = 1, \ldots, 3$,

$$s_{1i} = s_{11}^{\text{road}} + \cos(\theta^*) \cdot d_i$$
$$s_{2i} = s_{21}^{\text{road}} + \sin(\theta^*) \cdot d_i$$

7. Compute angle from $\boldsymbol{s}_i$ to μ: for $i = 1, \ldots, 3$, $\theta_i = \tan^{-1}\left(\frac{\mu_2 - s_{2i}}{\mu_1 - s_{1i}}\right)$

8. For each θ_i, draw $\theta_i^{\text{adj}} \sim \text{vonMises}\left(\tilde{\theta} = \theta_i - \pi, \kappa = 100\right)$
