# Supplementary material for: Accounting for location uncertainty in azimuthal telemetry data improves ecological inference
Source: Mov Ecol. 2018 Jul 25;6:14. doi: 10.1186/s40462-018-0129-1 (PMC6058391; doi:10.1186/s40462-018-0129-1)
Supplement: Supplementary file 5 — Details of the resource selection analysis simulation. (PDF 1106 kb) [file 40462_2018_129_MOESM5_ESM.pdf]

Appendix 5

March 4, 2018

Gerber, B. D., M. B. Hooten, C. P. Peck, M. B. Rice, J. H. Gammonley, A. D. Apa, and A. J. Davis. 2018. Accounting for location uncertainty in azimuthal telemetry data improves ecological inference.

RSF Simulation Study

It has been previously shown that factors such as covariate heterogeneity (i.e., spatial autocorrelation) and spatial resolution can affect whether location uncertainty is problematic to inference [1]. This is particularly relevant for spatial locations lying on the boundary of a categorical variable when accounting for location uncertainty. We performed the following simulation study to investigate the issue of location uncertainty for boundary cases under varying levels of covariate heterogeneity.

Methods

We first simulated continuous landscape covariates as a Gaussian random field with low (range parameter $\phi = 0.01$), moderate ($\phi = 0.1$), or high autocorrelation ($\phi = 0.25$). A categorical covariate was derived from each continuous covariate by setting a threshold to discretize the space. The resulting covariate pairs are displayed in Figure 1. Spatial locations were then simulated as an inhomogeneous Poisson spatial point process as a function of the continuous (γ_1) and categorical (γ_2) covariate with $w(\mathbf{x}'\boldsymbol{\gamma}) = e^{\gamma_0+\gamma_1 x_1+\gamma_2 x_2}$ on a subset of the spatial domain (Figure 1). Specifically, we used the following parameter specifications: $\gamma_0 = -4$, $\gamma_1 = 2$, and $\gamma_2 = -4$, which resulted in a sufficient number of locations near the boundary of the categorical covariate. To better understand the effect of sample size, we performed simulations for a low ($n = 50$) and large ($n = 200$) sample size. For each transmitter location, three observer locations were simulated using the random design detailed in ESM 5. Furthermore, simulated azimuths were randomly drawn from a von Mises distribution centered on the true azimuth with concentration parameter $\kappa = 50$. For each sample size and spatial autocorrelation combination, we fit both the RSF + ATM hierarchical model which accounts for location uncertainty and a standard RSF using Lenth estimated locations and the known locations (both of which do not account for location uncertainty). The RSF fit using the known locations provides our best estimate of the true selection coefficient and thus provides a reference to which comparisons can be made.

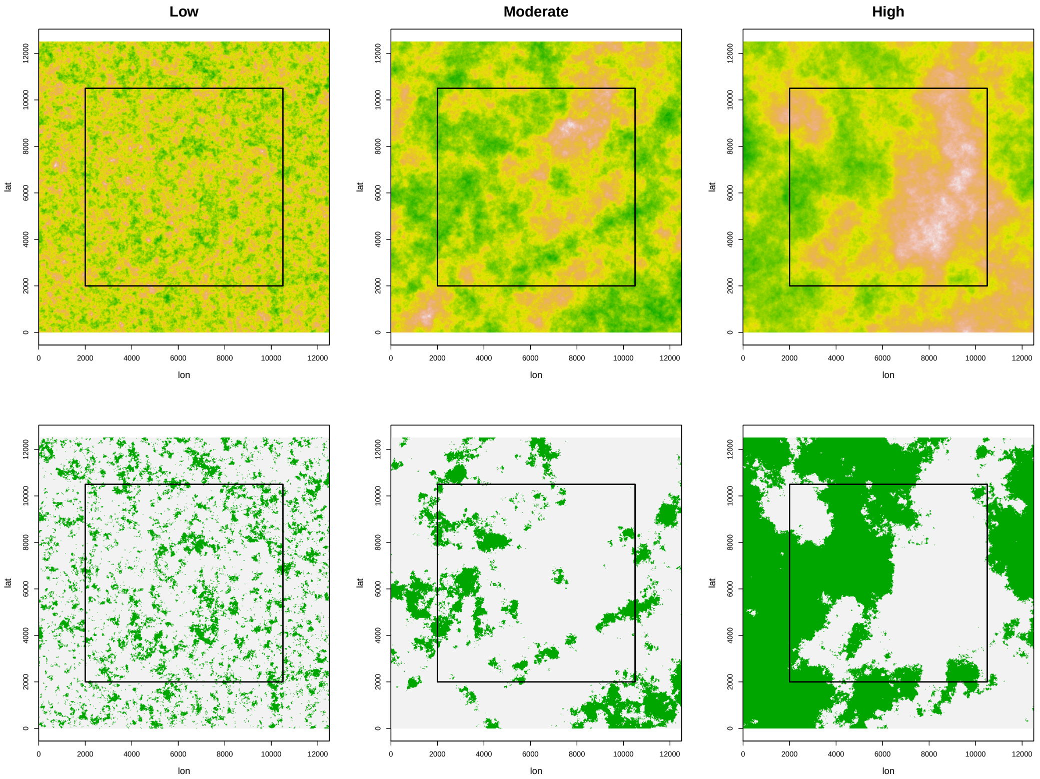

Figure 1. Simulated continuous and categorical landscape covariates used in the simulation study investigating location uncertainty for boundary cases under varying levels of covariate heterogeneity. Inner bounding box represents subset of spatial domain in which spatial locations were simulated.

Considerations

It is well documented that the mismatch in spatial extent between the availability and used locations can substantially influence coefficient estimates and inference (see [2] and the references therein).

References

[1] Robert A Montgomery, Gary J Roloff, and Jay M Ver Hoef. Implications of ignoring telemetry error on inference in wildlife resource use models. *The Journal of Wildlife Management*, 75(3):702–708, 2011.

[2] Joseph M Northrup, Mevin B Hooten, Charles R Anderson, and George Wittemyer. Practical guidance on characterizing availability in resource selection functions under a use–availability design. *Ecology*, 94(7):1456–1463, 2013.
