# Supplementary material for: Accounting for location uncertainty in azimuthal telemetry data improves ecological inference
Source: Mov Ecol. 2018 Jul 25;6:14. doi: 10.1186/s40462-018-0129-1 (PMC6058391; doi:10.1186/s40462-018-0129-1)
Supplement: Supplementary file 6 — Estimated home ranges from different individual Gunnison sage-grouse. (PDF 273 kb) [file 40462_2018_129_MOESM6_ESM.pdf]

Appendix 6

March 3, 2018

Gerber, B. D., M. B. Hooten, C. P. Peck, M. B. Rice, J. H. Gammonley, A. D. Apa, and A. J. Davis. 2018. Accounting for location uncertainty in azimuthal telemetry data improves ecological inference.

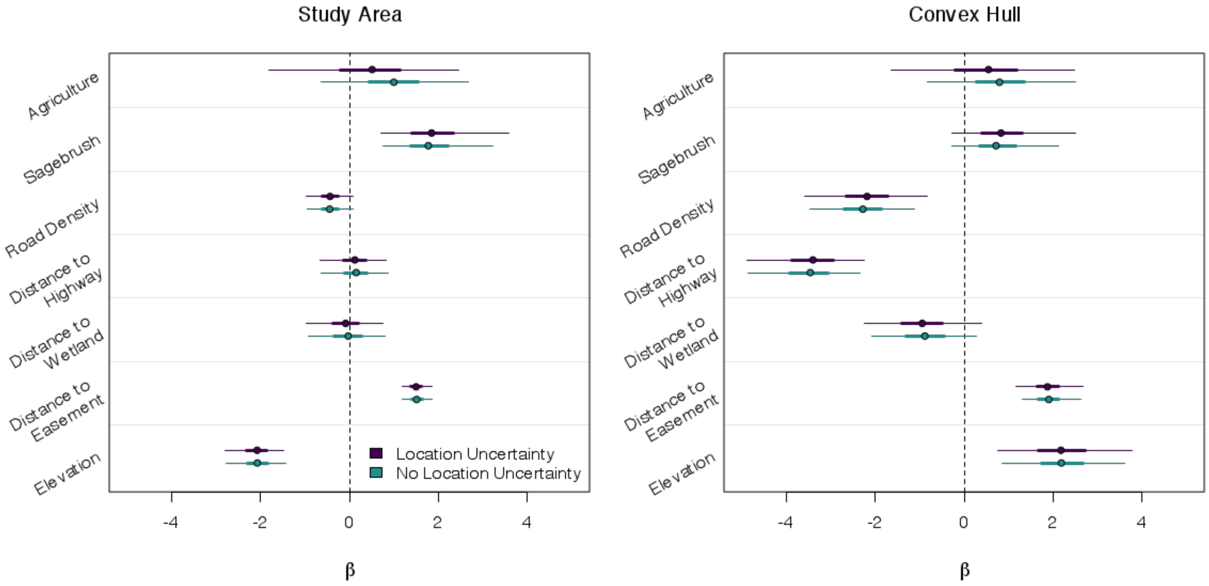

Figure 1. Resource selection coefficients for an individual Gunnison sage-grouse, in which location uncertainty is appropriately propagated and when it is ignored. Points represent posterior medians, thick lines are 50% credible intervals and thin lines represent 95% credible intervals. Evidence for selection or avoidance beyond the availability of a resource is supported by increasing positive or negative probability density, respectively.

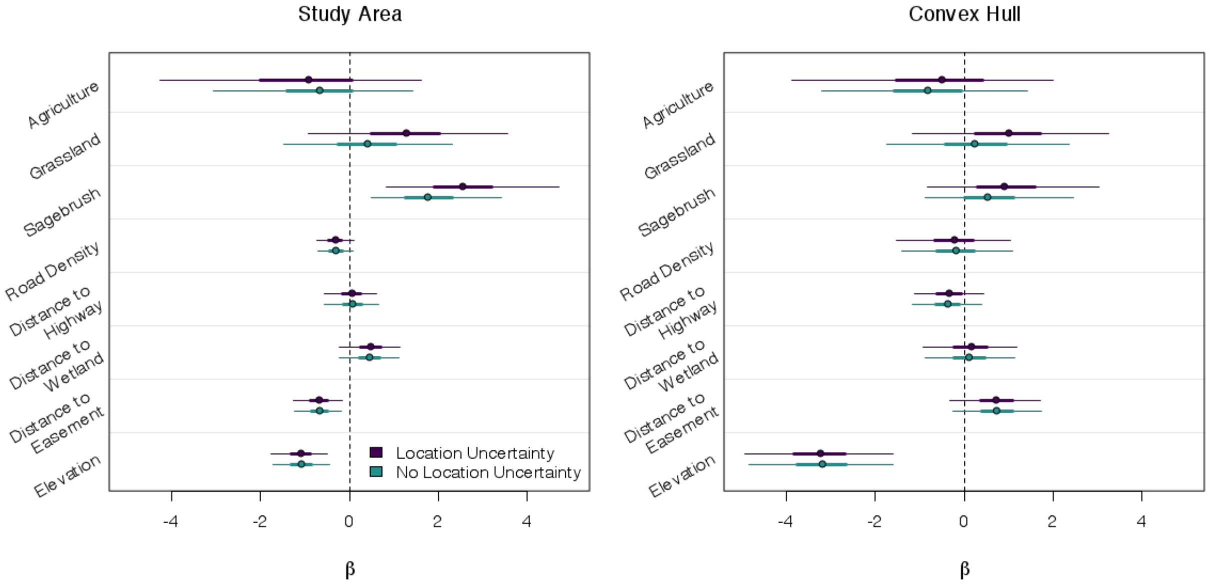

Figure 2. Resource selection coefficients for an individual Gunnison sage-grouse, in which location uncertainty is appropriately propagated and when it is ignored. Points represent posterior medians, thick lines are 50% credible intervals and thin lines represent 95% credible intervals. Evidence for selection or avoidance beyond the availability of a resource is supported by increasing positive or negative probability density, respectively.

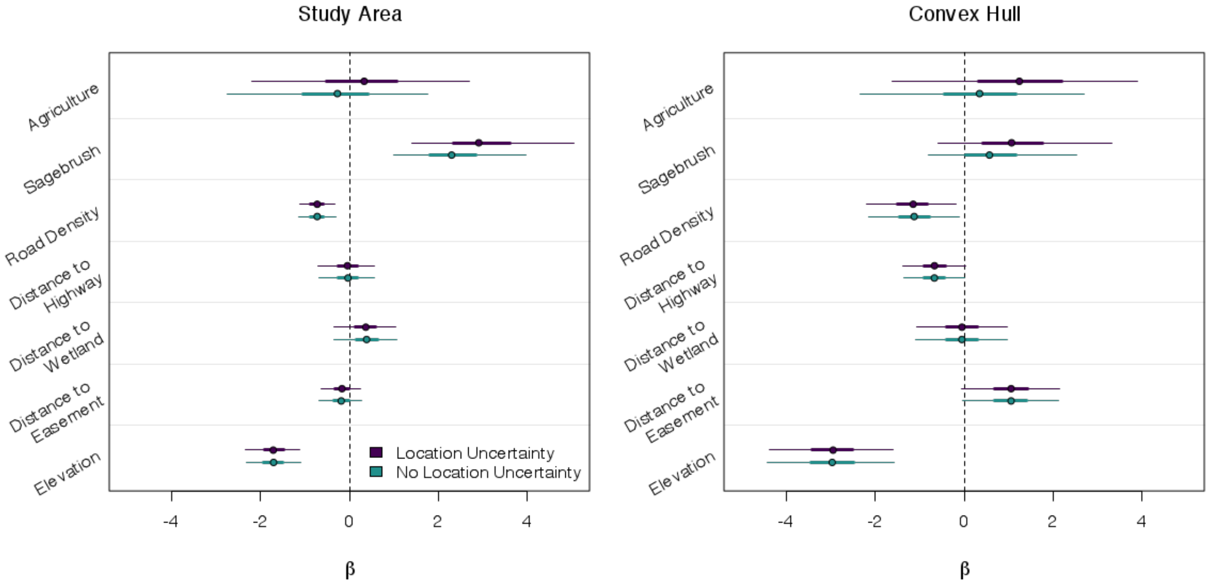

Figure 3. Resource selection coefficients for an individual Gunnison sage-grouse, in which location uncertainty is appropriately propagated and when it is ignored. Points represent posterior medians, thick lines are 50% credible intervals and thin lines represent 95% credible intervals. Evidence for selection or avoidance beyond the availability of a resource is supported by increasing positive or negative probability density, respectively.

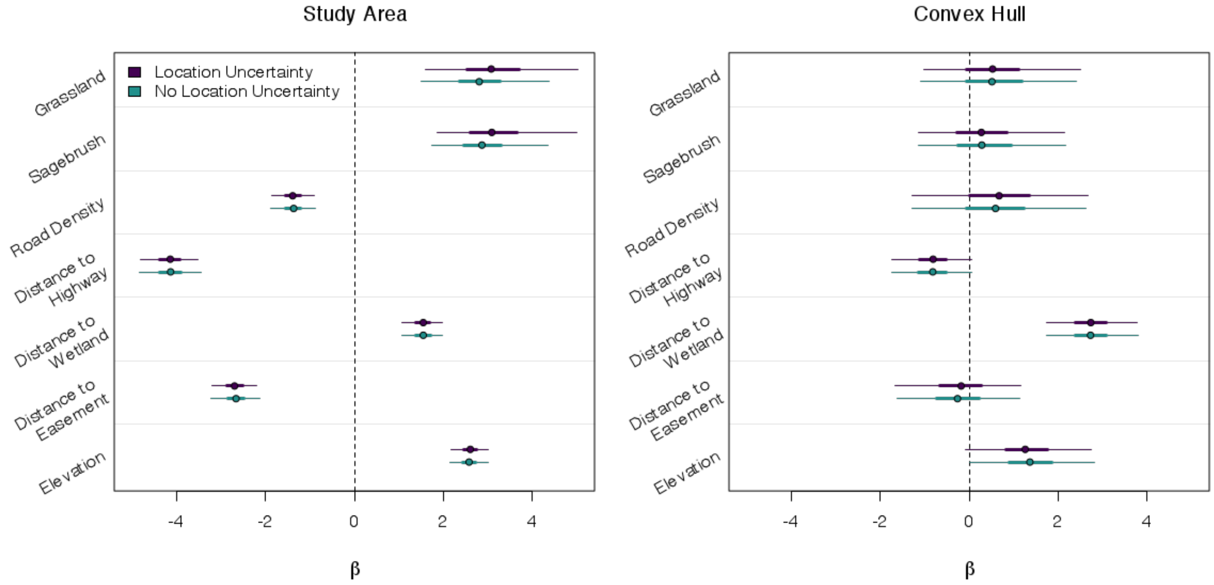

Figure 4. Resource selection coefficients for an individual Gunnison sage-grouse, in which location uncertainty is appropriately propagated and when it is ignored. Points represent posterior medians, thick lines are 50% credible intervals and thin lines represent 95% credible intervals. Evidence for selection or avoidance beyond the availability of a resource is supported by increasing positive or negative probability density, respectively.

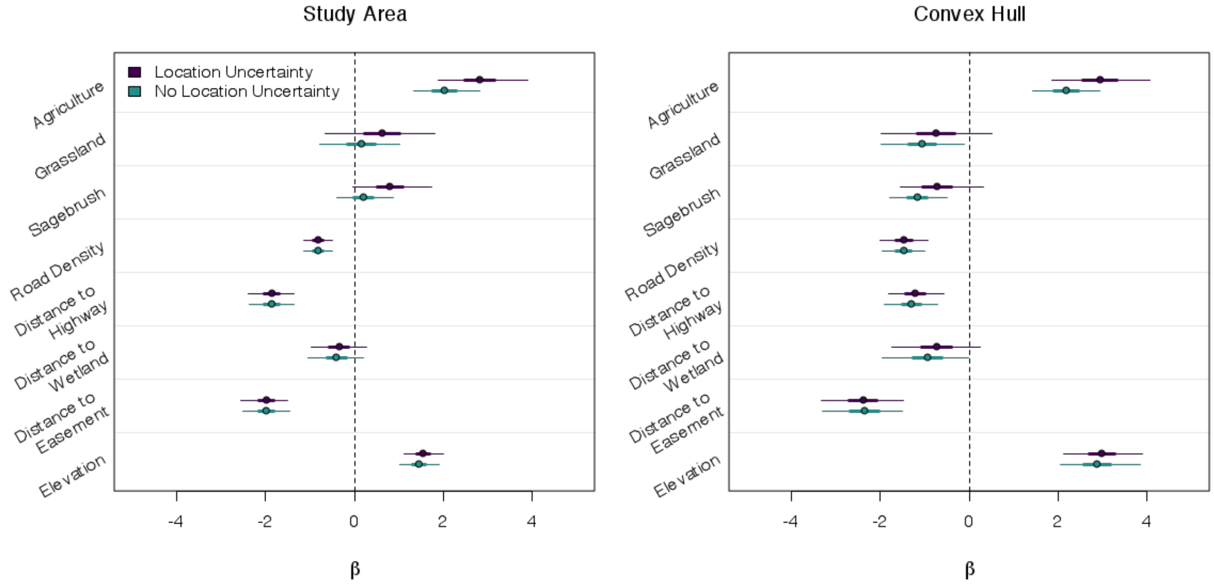

Figure 5. Resource selection coefficients for an individual Gunnison sage-grouse, in which location uncertainty is appropriately propagated and when it is ignored. Points represent posterior medians, thick lines are 50% credible intervals and thin lines represent 95% credible intervals. Evidence for selection or avoidance beyond the availability of a resource is supported by increasing positive or negative probability density, respectively.
