# Supplementary material for: Accounting for location uncertainty in azimuthal telemetry data improves ecological inference
Source: Mov Ecol. 2018 Jul 25;6:14. doi: 10.1186/s40462-018-0129-1 (PMC6058391; doi:10.1186/s40462-018-0129-1)
Supplement: Supplementary file 7 — Estimated resource selection coefficients from different individual Gunnison sage-grouse. (PDF 9042 kb) [file 40462_2018_129_MOESM7_ESM.pdf]

Appendix 7

October 30, 2017

Home Range

For six individual Gunnison sage-grouse, we estimate home range for two seasons (Breeding and Summer) across multiple years. For each figure below, home range area estimates for sage-grouse are estimated via convex hull (top plot) and kernel estimation (bottom plot) where spatial location uncertainty is incorporated via the azimuthal telemetry model (ATM) or ignored using Lenth (1981) estimation. Right-sided figures depict a histogram of the posterior distribution of the area of the home range when accounting for animal location uncertainty via the ATM; the vertical line is the point estimate of home range area when animal locations are estimated using Lenth (1981) estimation and location uncertainty is ignored.

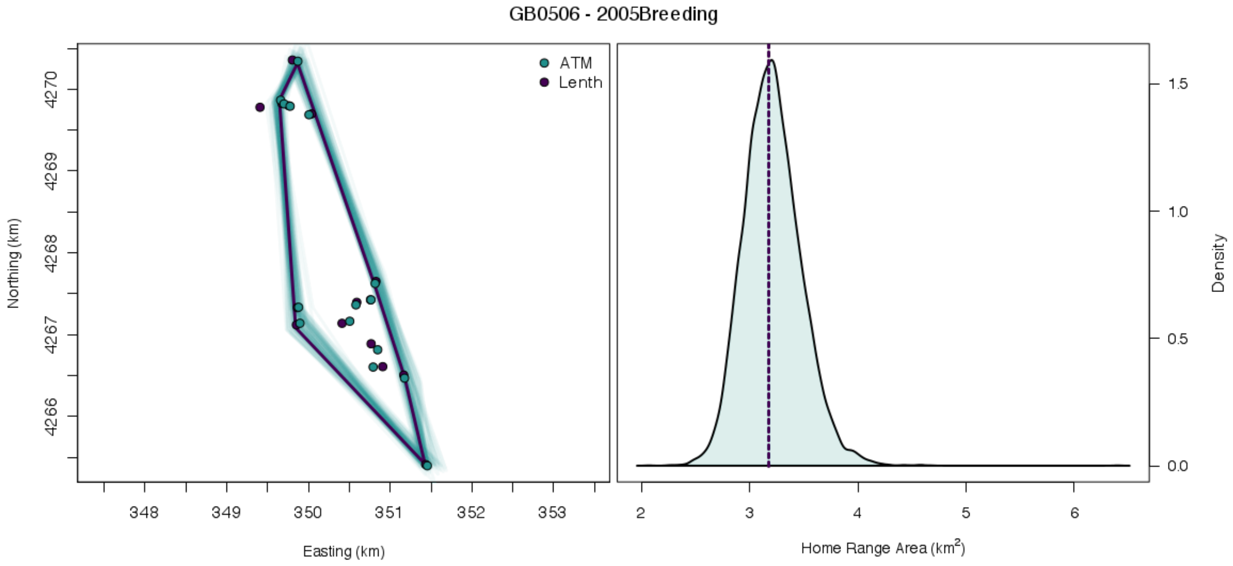

GB0506 - 2005Breeding
ATM
Lenth
Northing (km)
Easting (km)
Home Range Area (km2)
Density

(a)

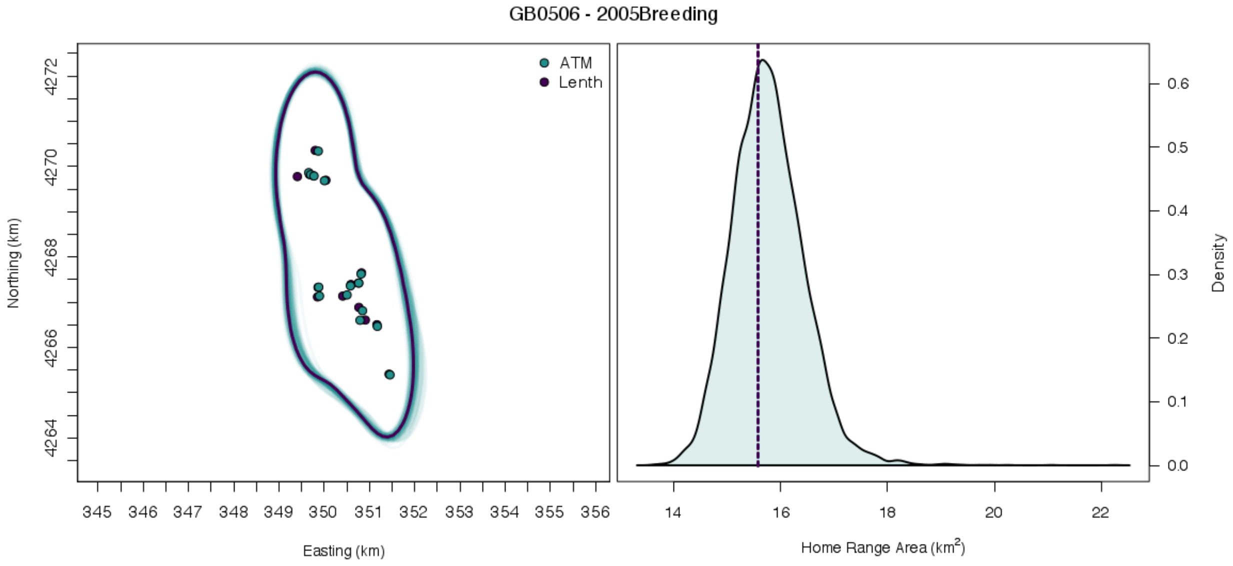

GB0506 - 2005Breeding
ATM
Lenth
Northing (km)
Easting (km)
Home Range Area (km2)
Density

(b)

Figure 1

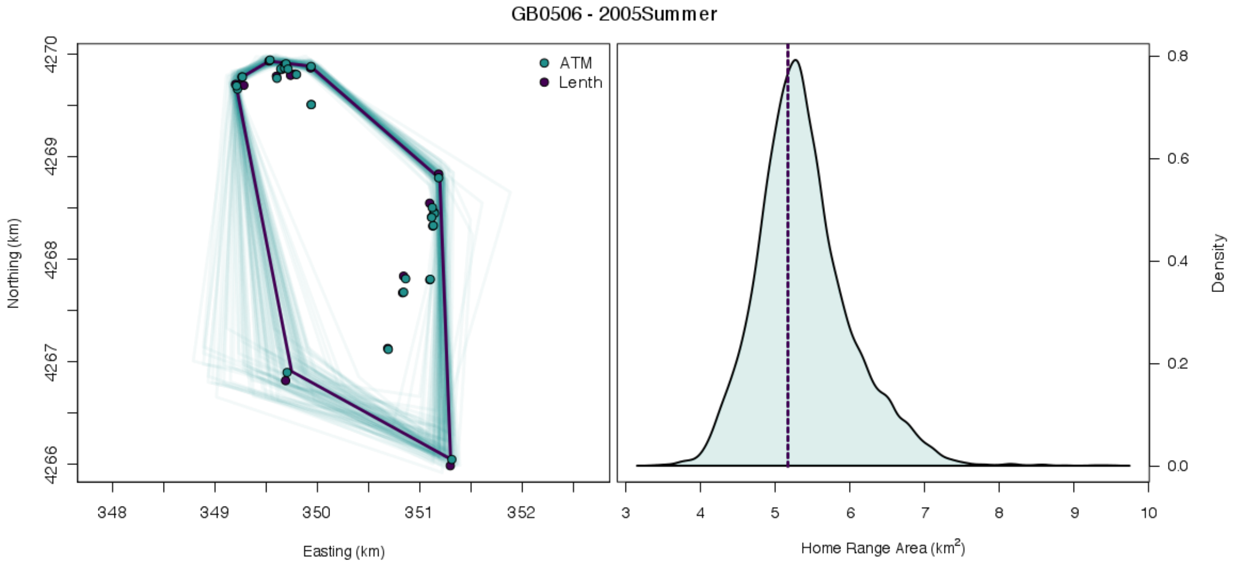
GB0506 - 2005Summer
ATM
Lenth
Northing (km)
4266
4267
4268
4269
4270
348
349
350
351
352
Easting (km)
3
4
5
6
7
8
9
10
Home Range Area (km²)
0.0
0.2
0.4
0.6
0.8
Density

(a)

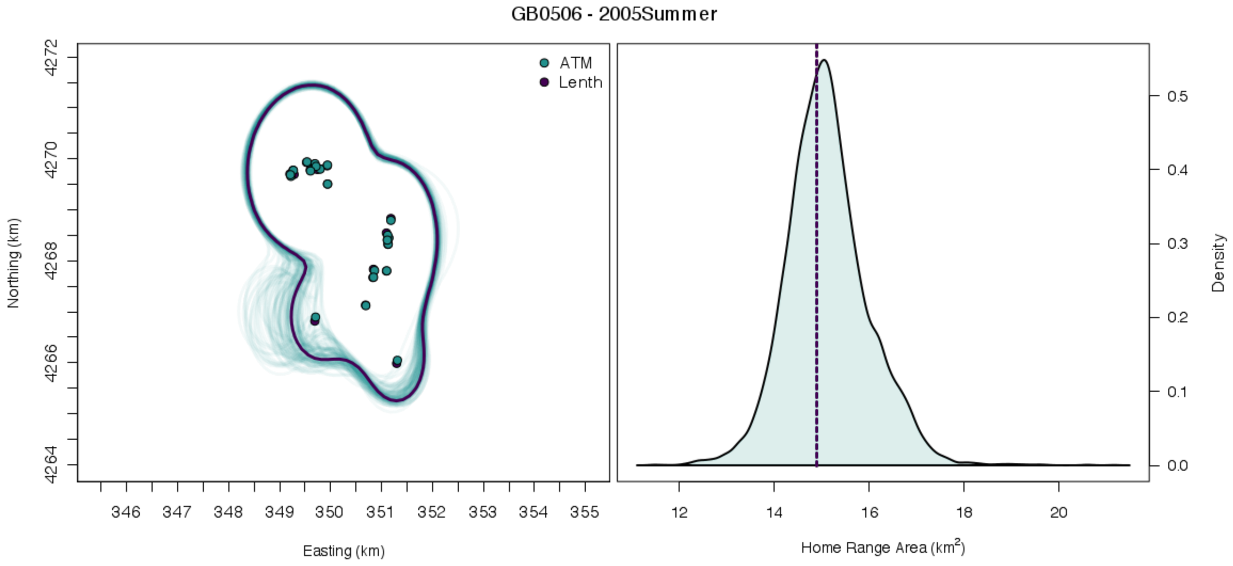
GB0506 - 2005Summer
ATM
Lenth
Northing (km)
4264
4266
4268
4270
4272
346
347
348
349
350
351
352
353
354
355
Easting (km)
12
14
16
18
20
Home Range Area (km²)
0.0
0.1
0.2
0.3
0.4
0.5
Density

(b)

Figure 2

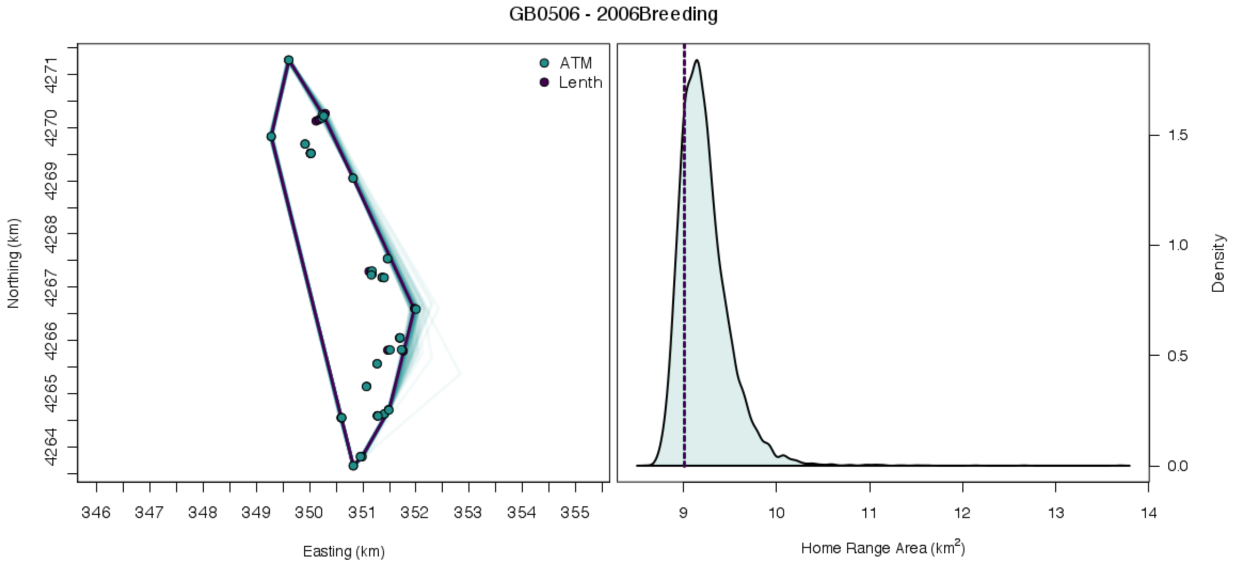
GB0506 - 2006Breeding
ATM
Lenth
Northing (km)
Easting (km)
Home Range Area (km²)
Density

(a)

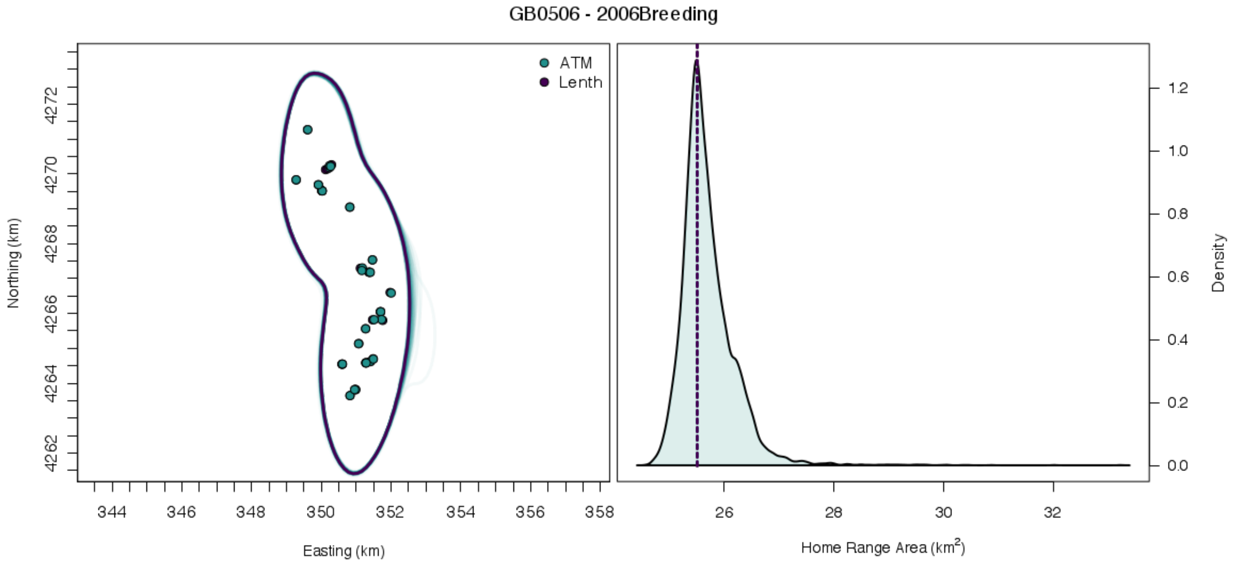
GB0506 - 2006Breeding
ATM
Lenth
Northing (km)
Easting (km)
Home Range Area (km²)
Density

(b)

Figure 3

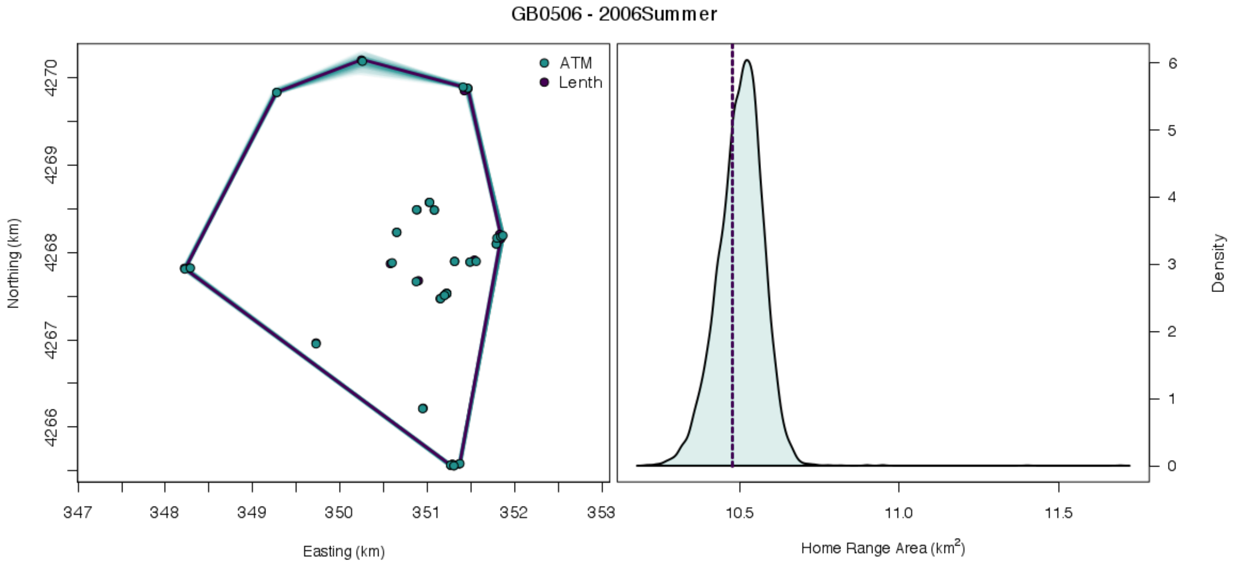
GB0506 - 2006Summer
ATM
Lenth
Northing (km)
Easting (km)
Density
Home Range Area (km^2)

(a)

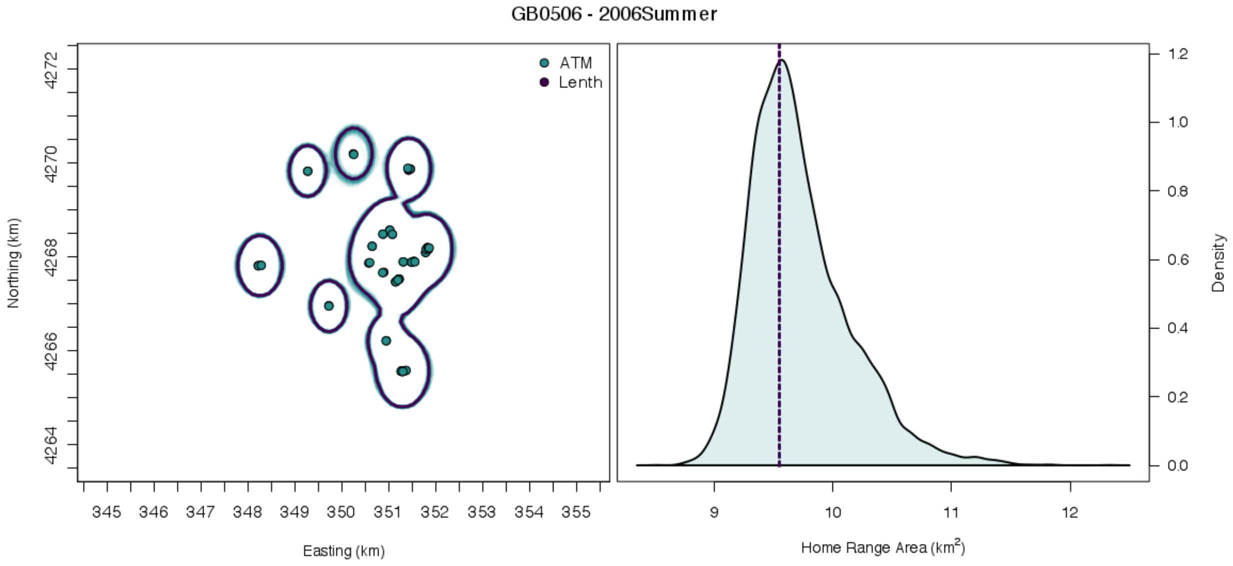
GB0506 - 2006Summer
ATM
Lenth
Northing (km)
Easting (km)
Density
Home Range Area (km^2)

(b)

Figure 4

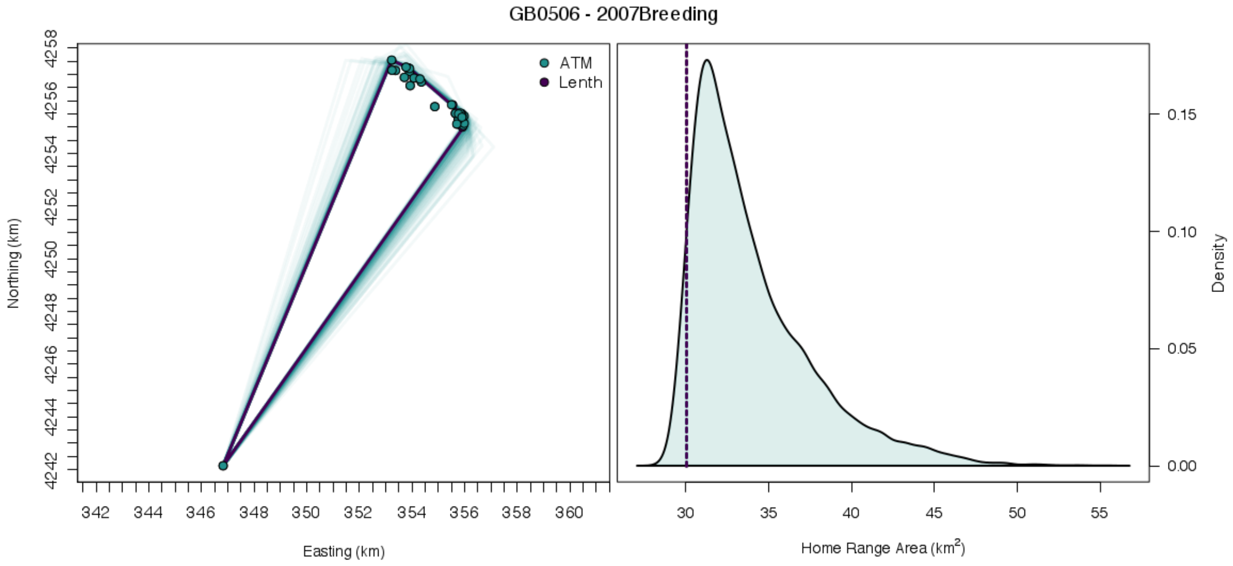
GB0506 - 2007Breeding
ATM
Lenth
Northing (km)
Easting (km)
Home Range Area (km^2)
Density

(a)

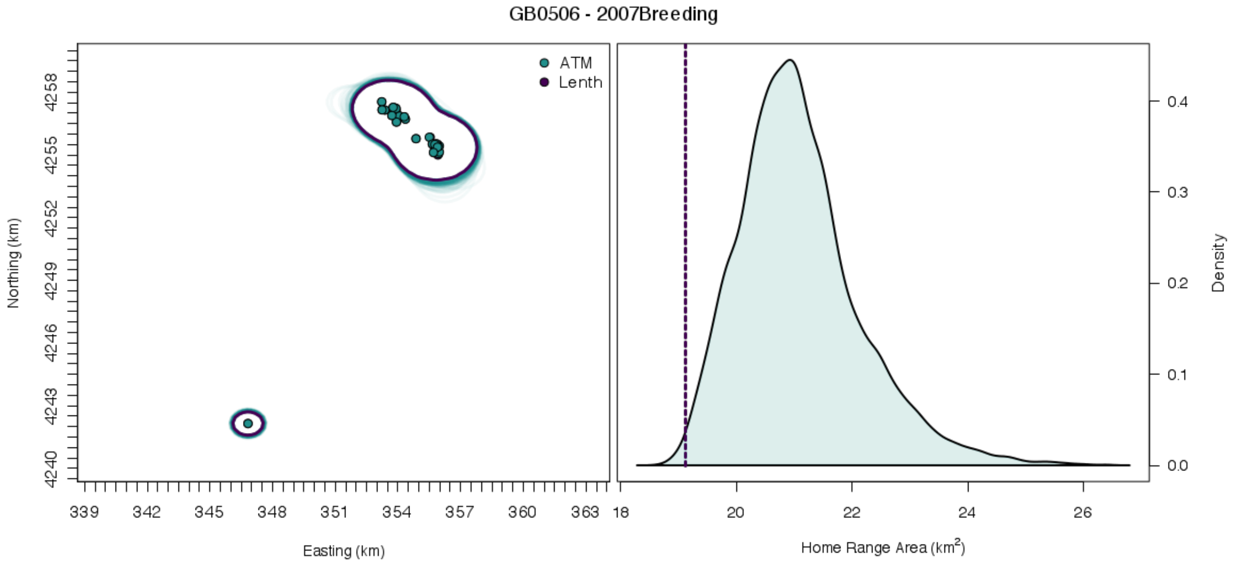
GB0506 - 2007Breeding
ATM
Lenth
Northing (km)
Easting (km)
Home Range Area (km^2)
Density

(b)

Figure 5

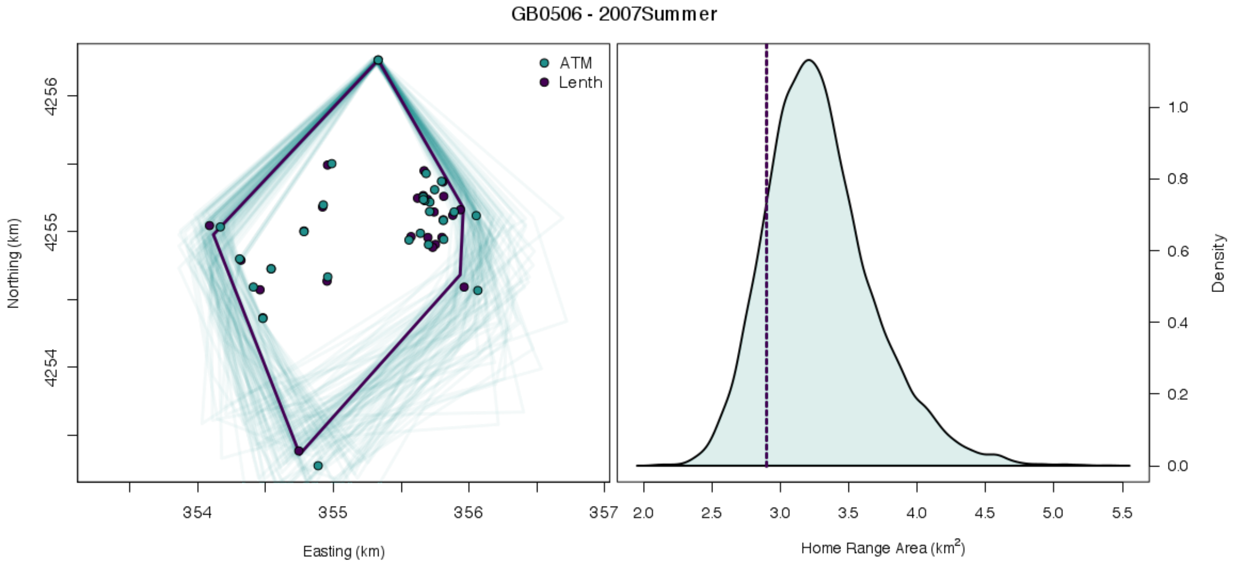
GB0506 - 2007Summer
ATM
Lenth
Northing (km)
4254
4255
4256
354
355
356
357
Easting (km)
2.0
2.5
3.0
3.5
4.0
4.5
5.0
5.5
Home Range Area (km^2)
0.0
0.2
0.4
0.6
0.8
1.0
Density

(a)

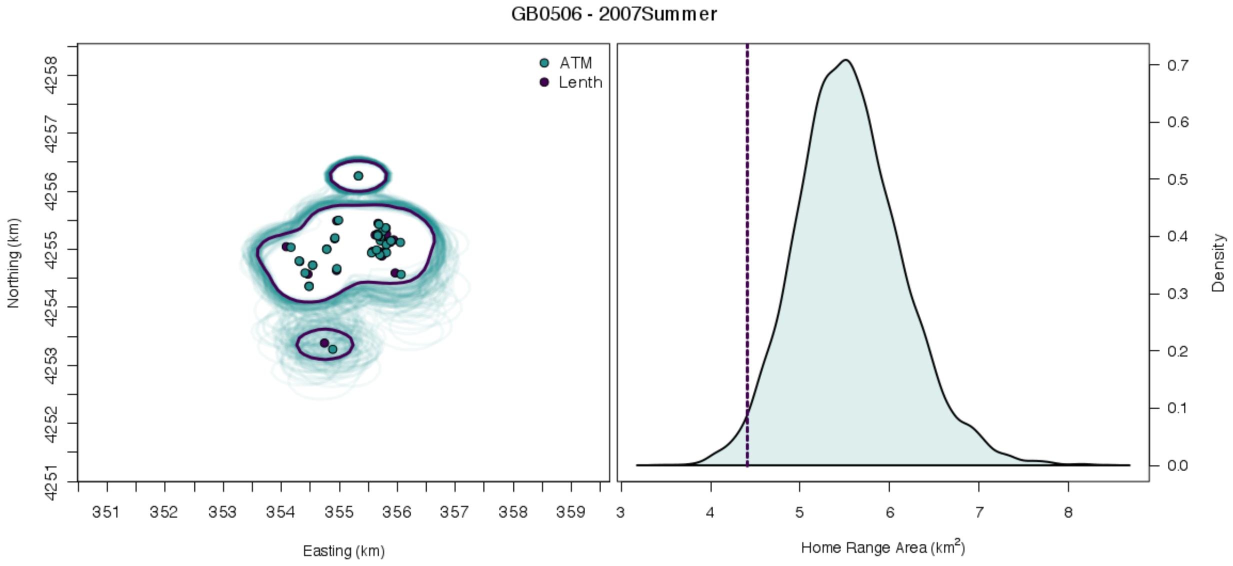
GB0506 - 2007Summer
ATM
Lenth
Northing (km)
4251
4252
4253
4254
4255
4256
4257
4258
351
352
353
354
355
356
357
358
359
Easting (km)
3
4
5
6
7
8
Home Range Area (km^2)
0.0
0.1
0.2
0.3
0.4
0.5
0.6
0.7
Density

(b)

Figure 6

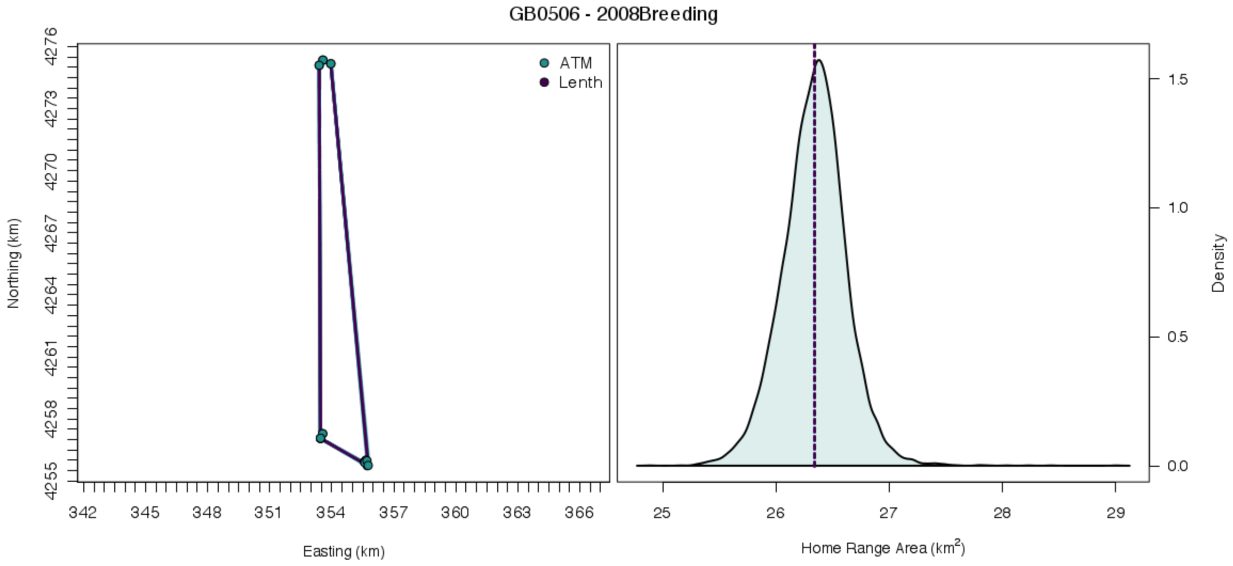
GB0506 - 2008Breeding
ATM
Lenth
Northing (km)
Easting (km)
Home Range Area (km^2)
Density

(a)

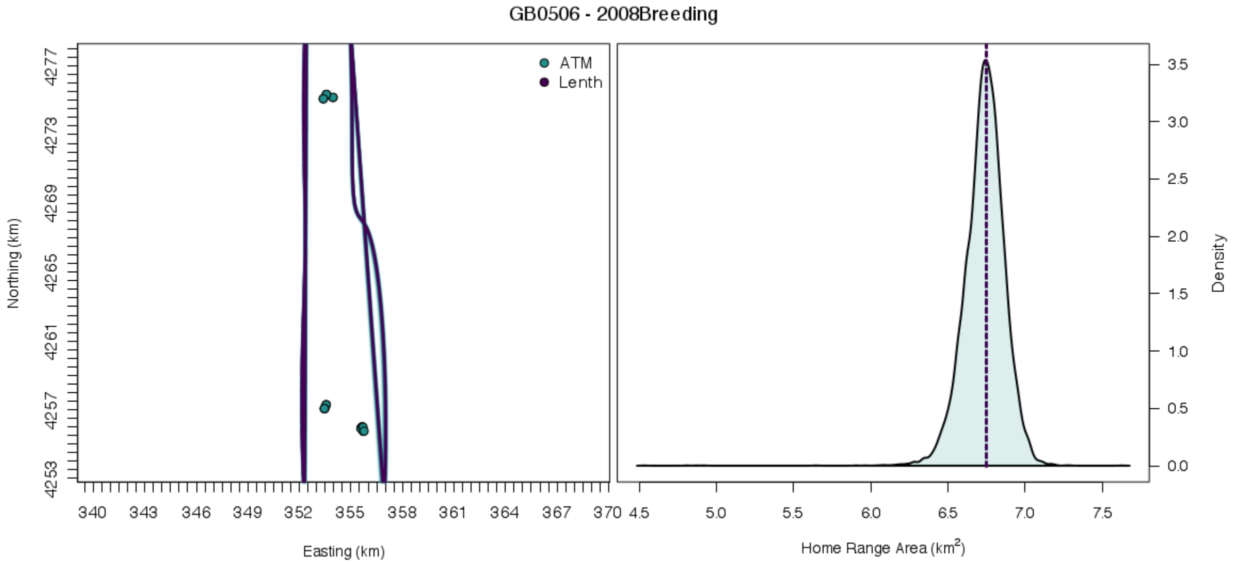
GB0506 - 2008Breeding
ATM
Lenth
Northing (km)
Easting (km)
Home Range Area (km^2)
Density

(b)

Figure 7

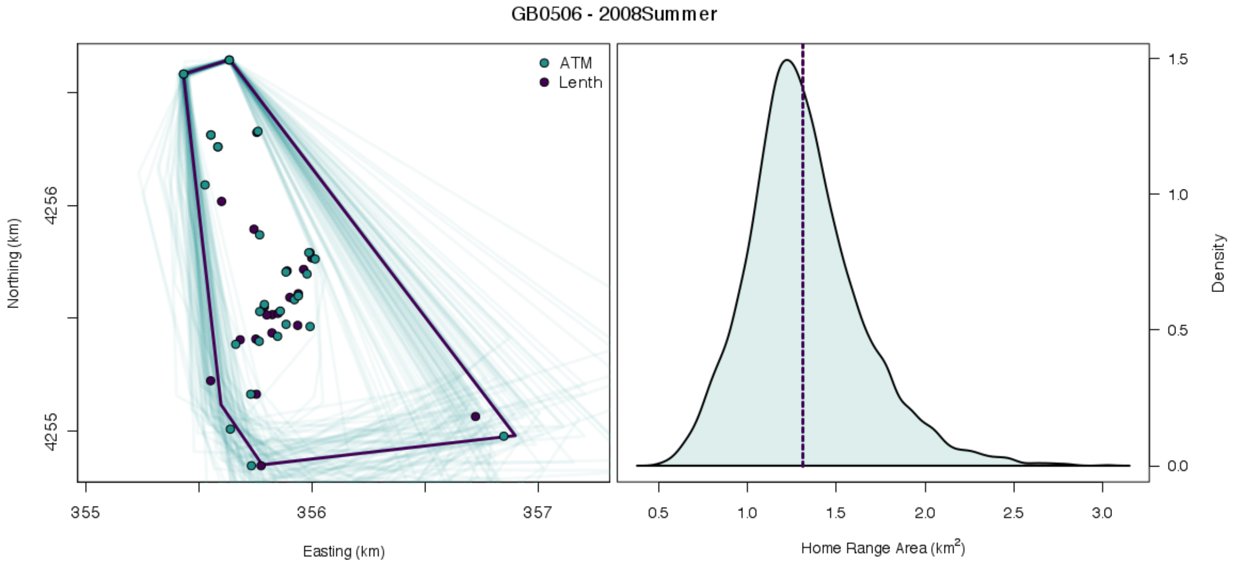
GB0506 - 2008Summer
ATM
Lenth
Northing (km)
4256
4255
355
356
357
Easting (km)
0.5
1.0
1.5
2.0
2.5
3.0
Home Range Area (km2)
Density
0.0
0.5
1.0
1.5

(a)

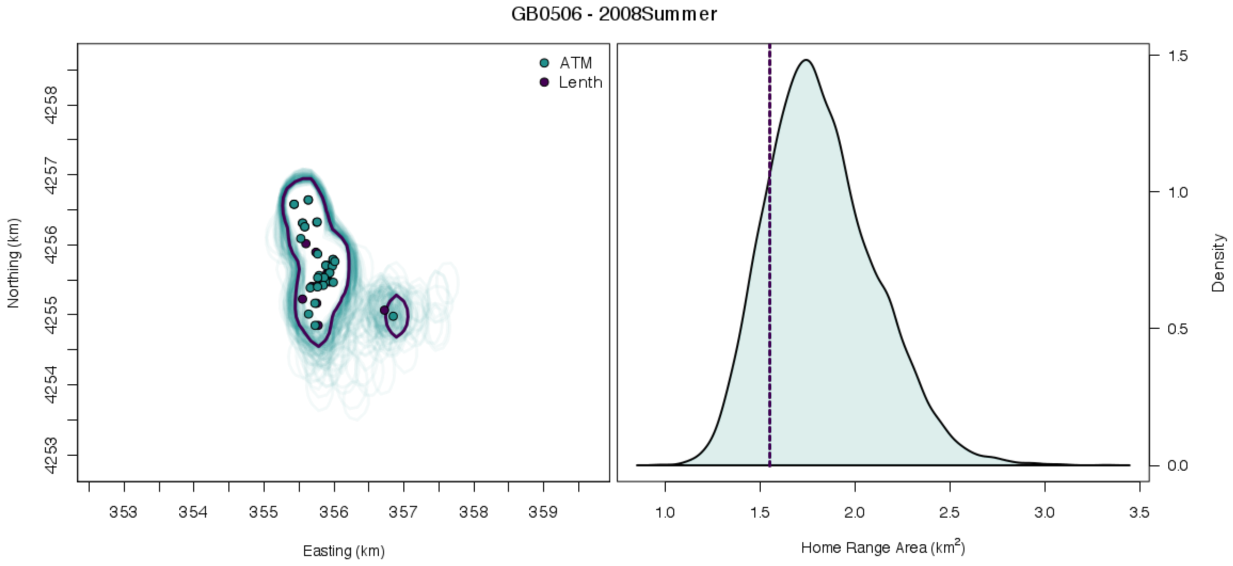
GB0506 - 2008Summer
ATM
Lenth
Northing (km)
4258
4257
4256
4255
4254
4253
353
354
355
356
357
358
359
Easting (km)
1.0
1.5
2.0
2.5
3.0
3.5
Home Range Area (km2)
Density
0.0
0.5
1.0
1.5

(b)

Figure 8

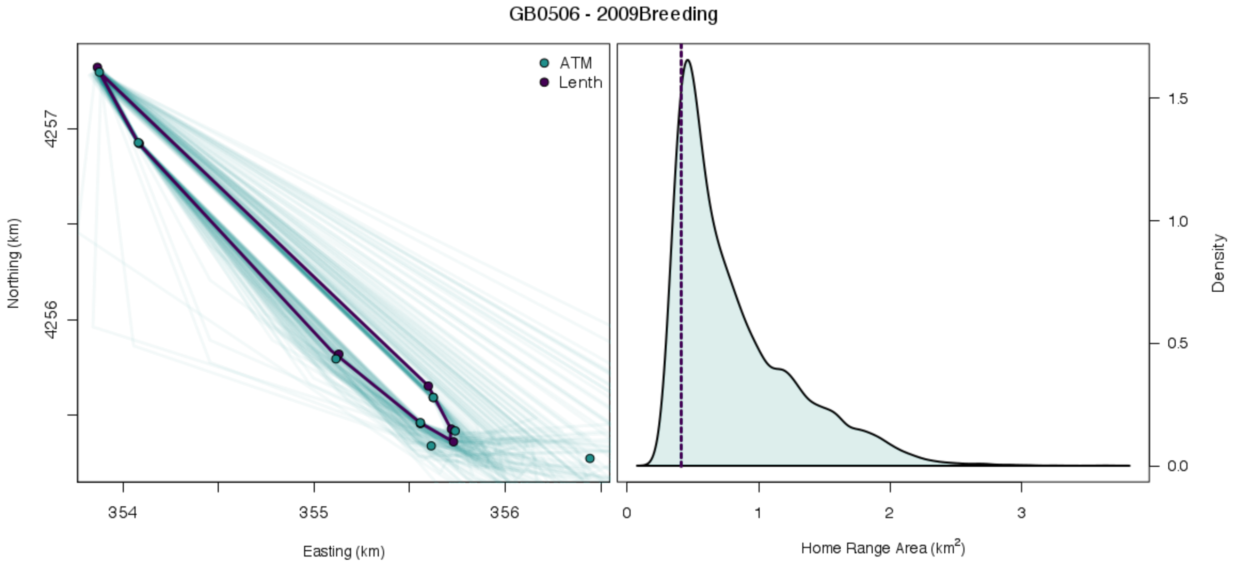
GB0506 - 2009Breeding
ATM
Lenth
Northing (km)
4257
4256
354
355
356
Easting (km)
0
1
2
3
Home Range Area (km2)
Density
1.5
1.0
0.5
0.0

(a)

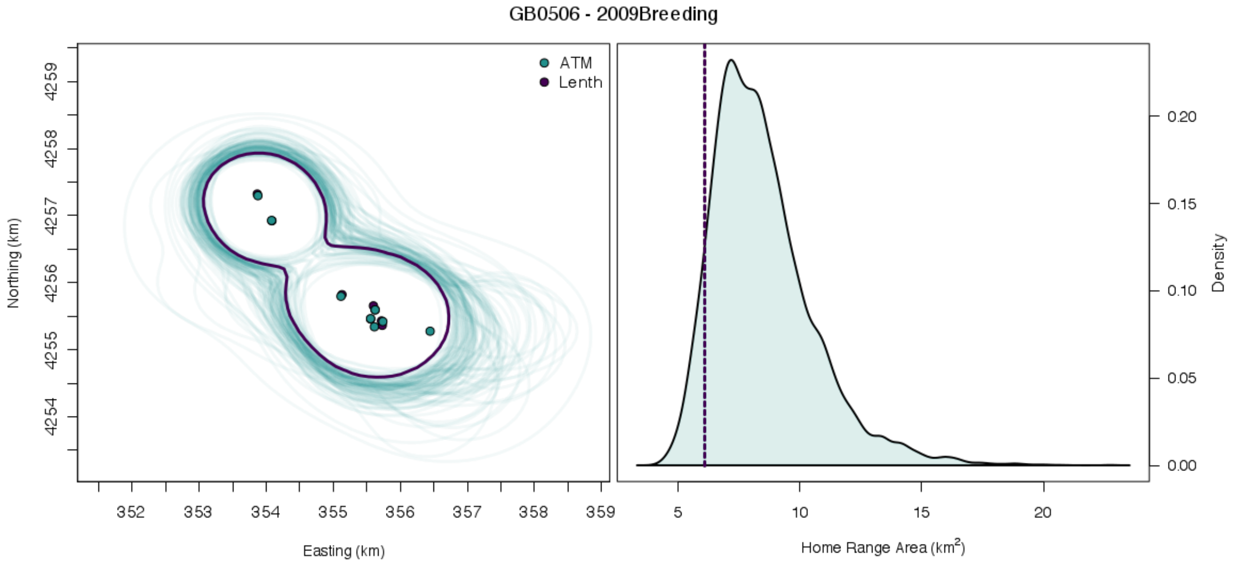
GB0506 - 2009Breeding
ATM
Lenth
Northing (km)
4259
4258
4257
4256
4255
4254
352
353
354
355
356
357
358
359
Easting (km)
5
10
15
20
Home Range Area (km2)
Density
0.20
0.15
0.10
0.05
0.00

(b)

Figure 9

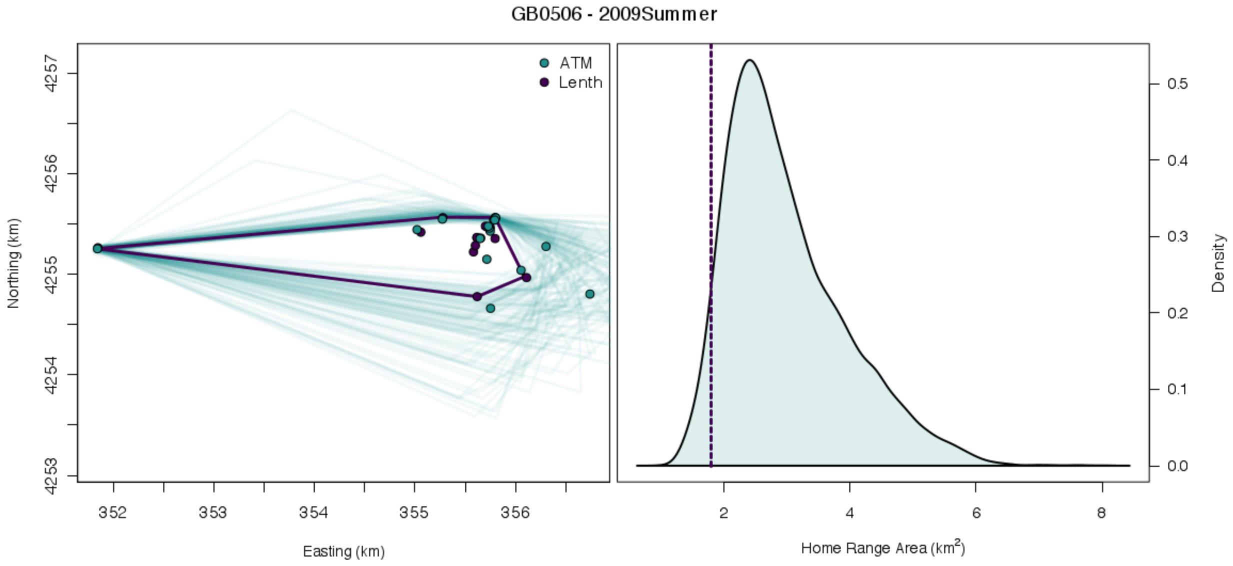
GB0506 - 2009Summer
ATM
Lenth
Northing (km)
Easting (km)
Home Range Area (km^2)
Density

(a)

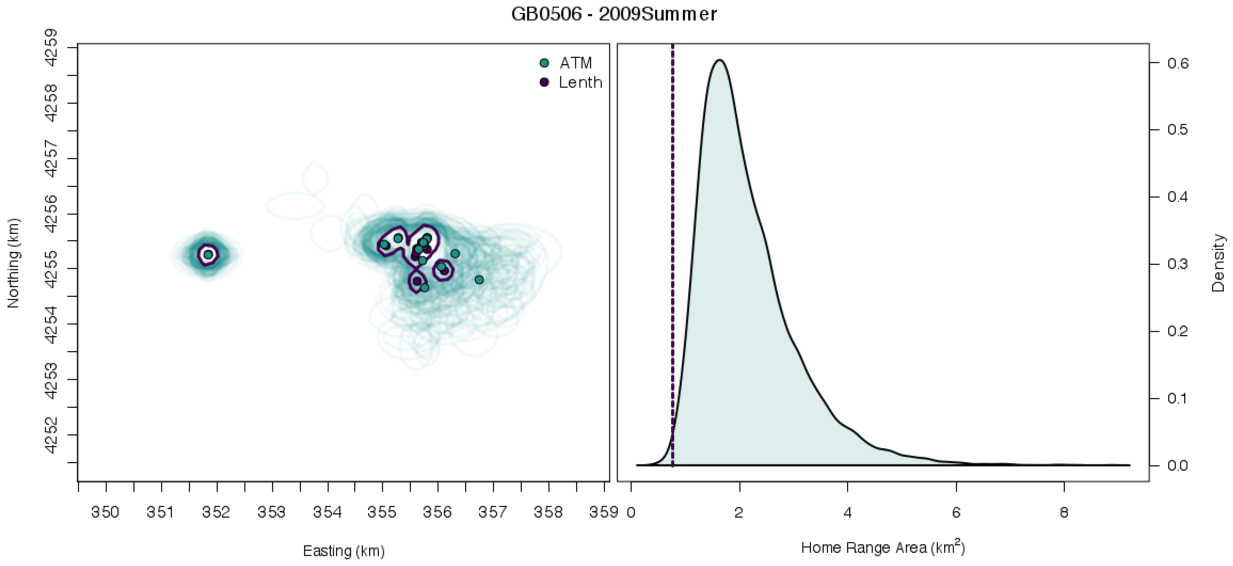
GB0506 - 2009Summer
ATM
Lenth
Northing (km)
Easting (km)
Home Range Area (km^2)
Density

(b)

Figure 10

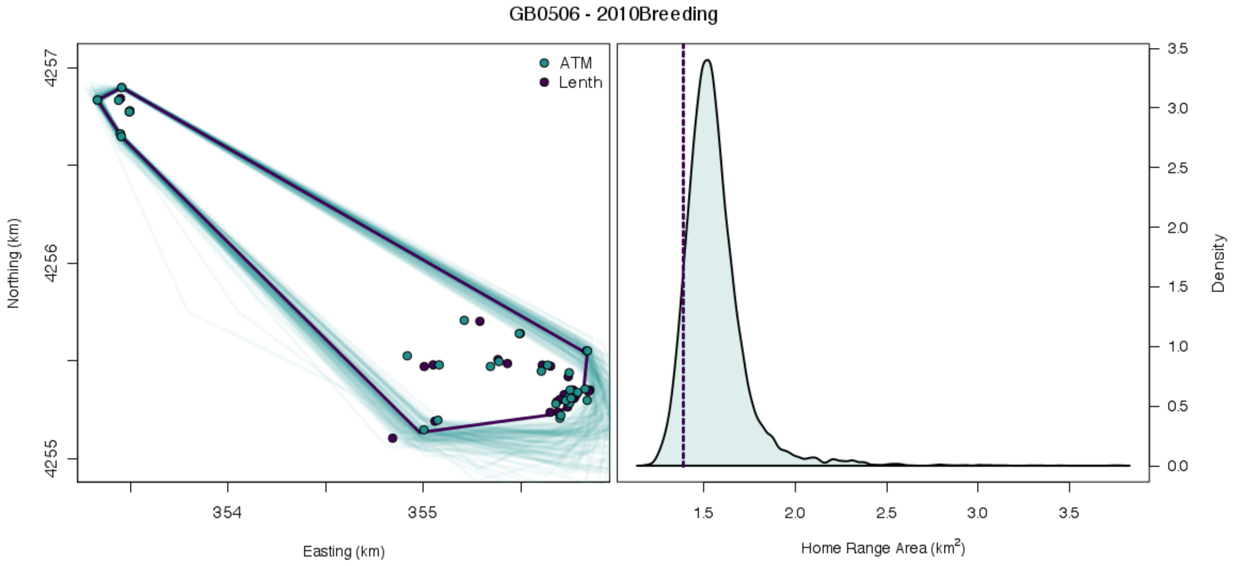
GB0506 - 2010Breeding
ATM
Lenth
Northing (km)
Easting (km)
Density
Home Range Area (km^2)

(a)

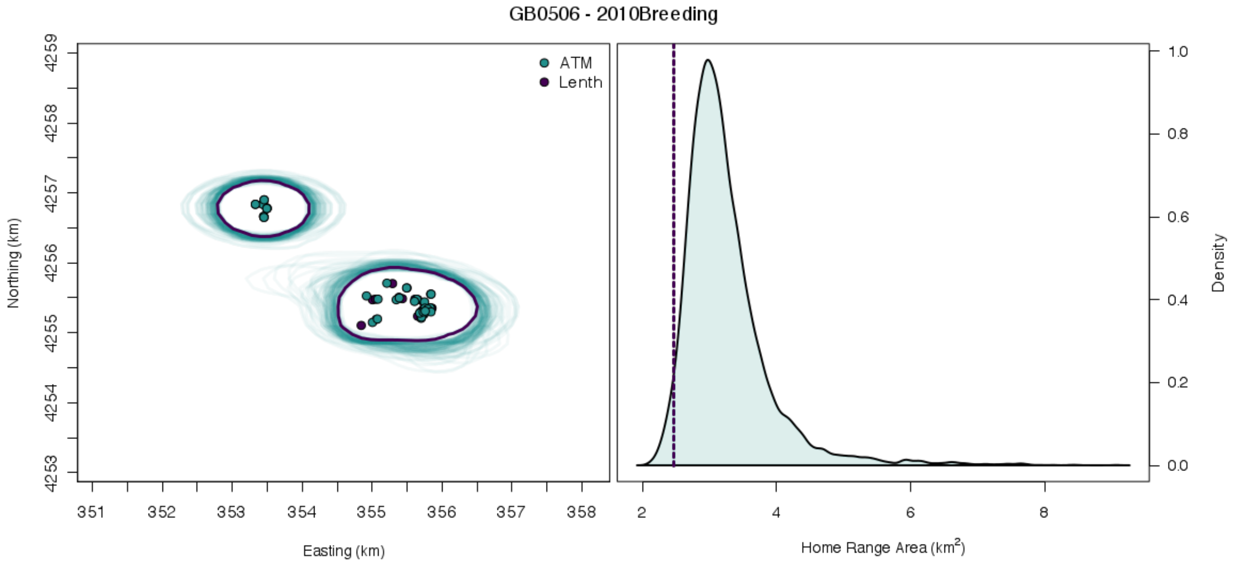
GB0506 - 2010Breeding
ATM
Lenth
Northing (km)
Easting (km)
Density
Home Range Area (km^2)

(b)

Figure 11

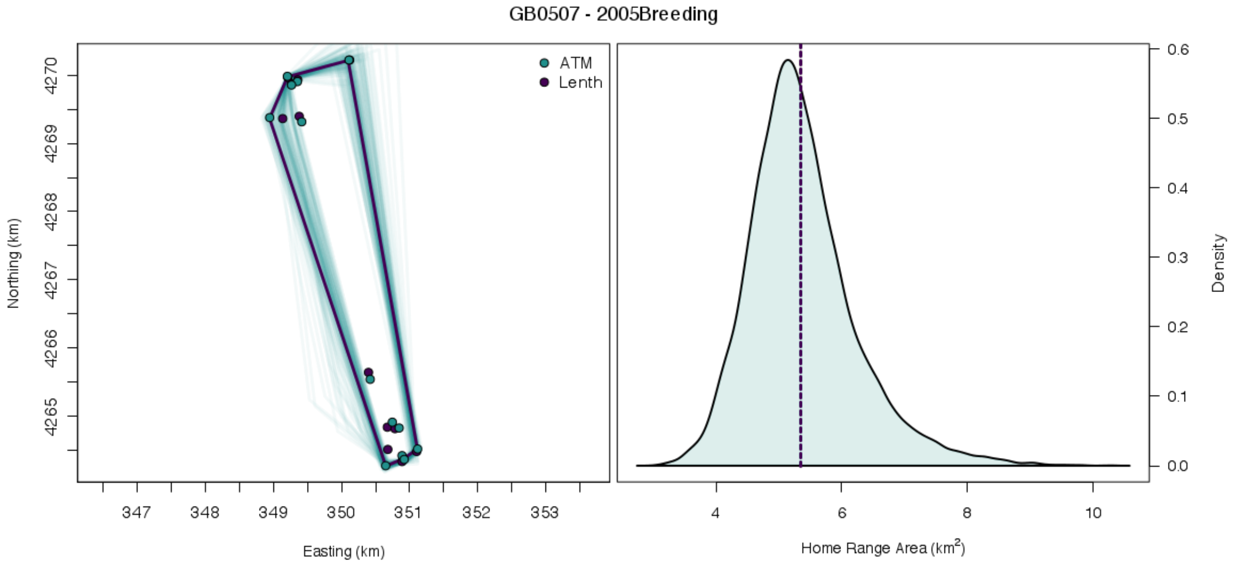
GB0507 - 2005Breeding
ATM
Lenth
Northing (km)
Easting (km)
Home Range Area (km^2)
Density

(a)

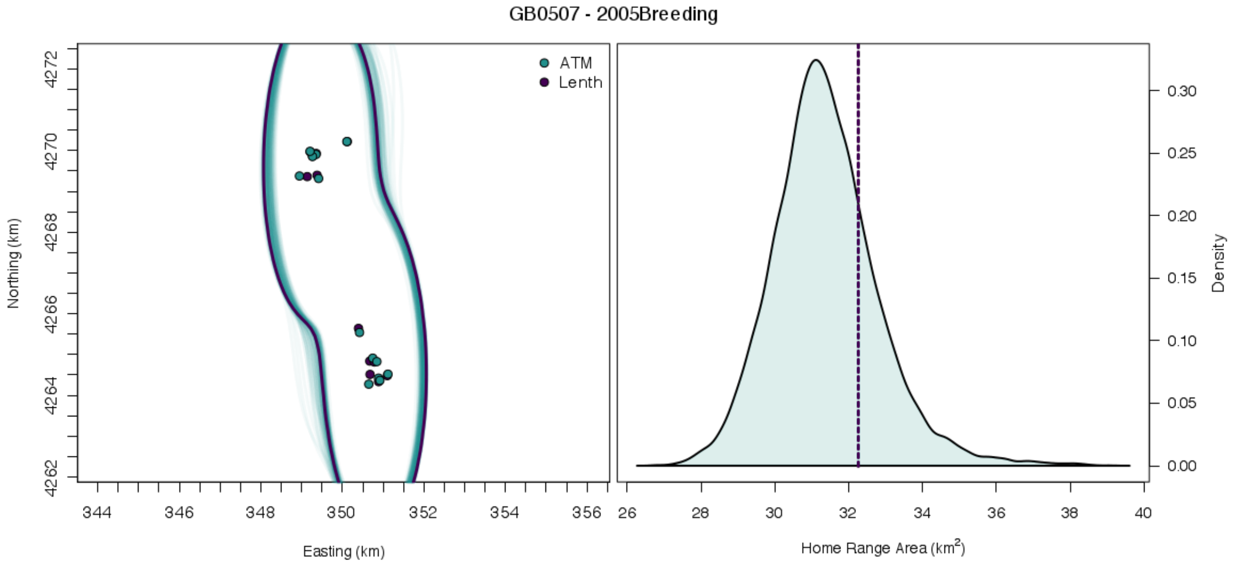
GB0507 - 2005Breeding
ATM
Lenth
Northing (km)
Easting (km)
Home Range Area (km^2)
Density

(b)

Figure 12

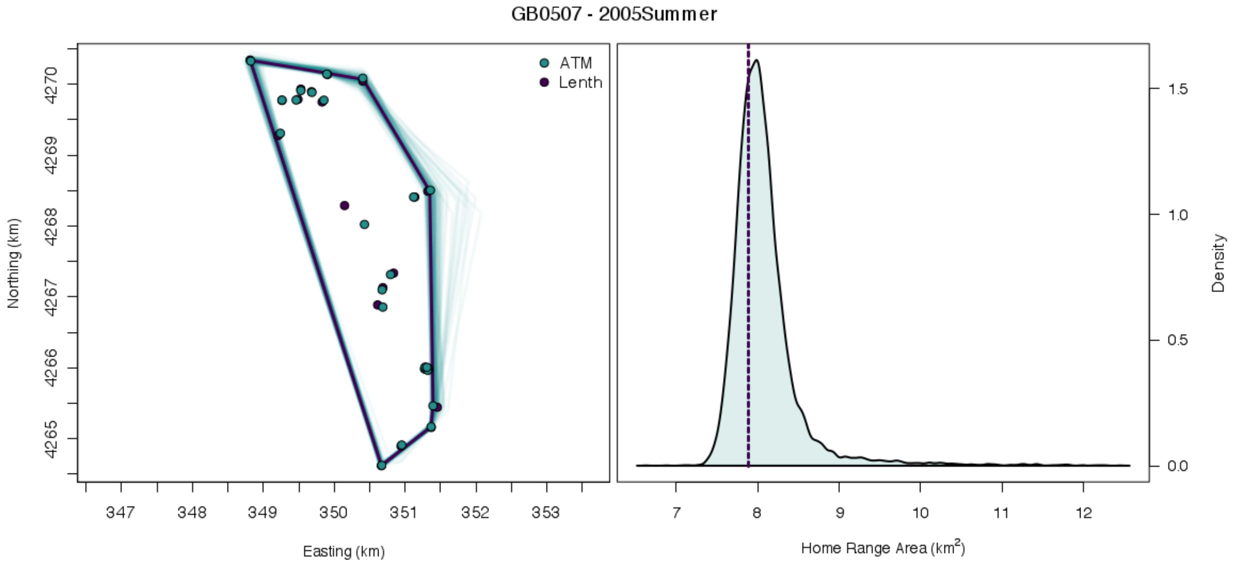
GB0507 - 2005Summer
ATM
Lenth
Northing (km)
Easting (km)
Home Range Area (km^2)
Density

(a)

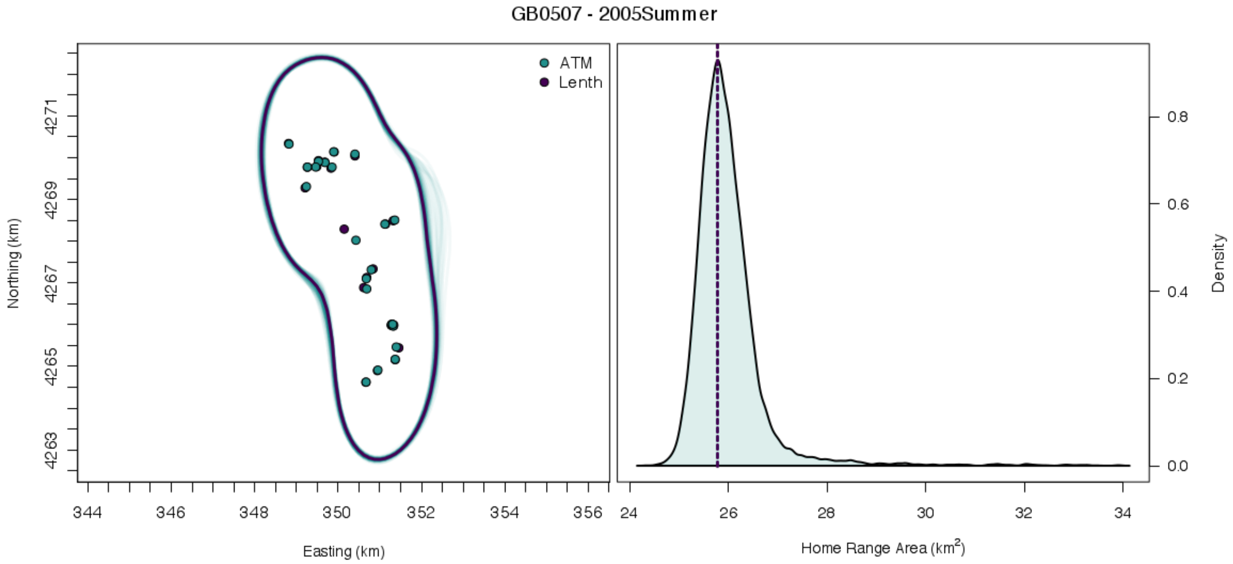
GB0507 - 2005Summer
ATM
Lenth
Northing (km)
Easting (km)
Home Range Area (km^2)
Density

(b)

Figure 13

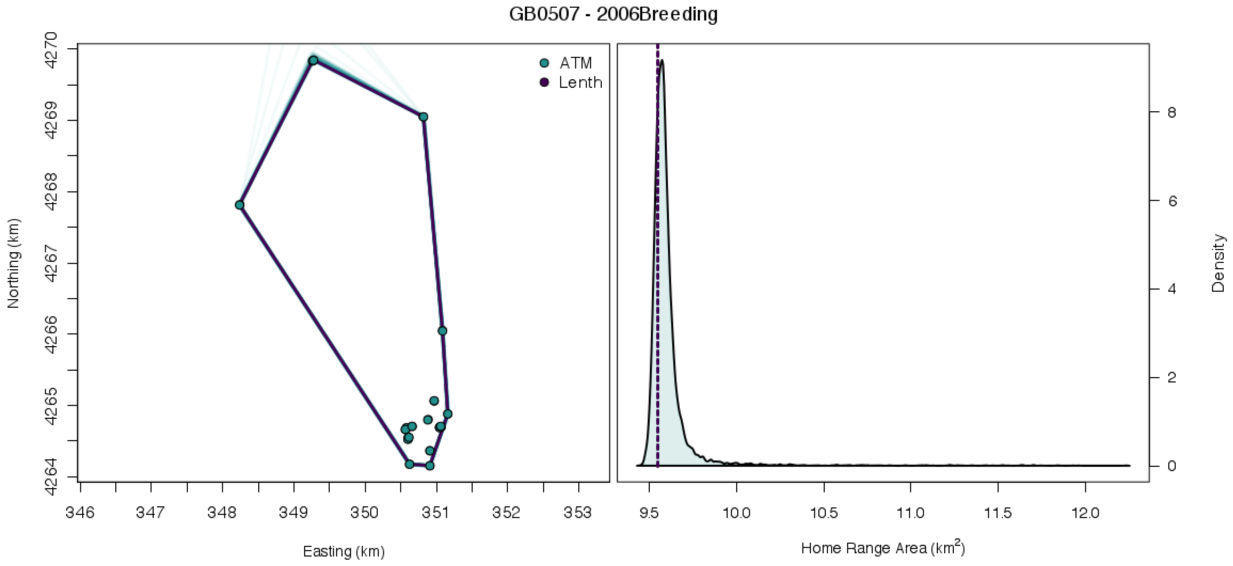
GB0507 - 2006Breeding
ATM
Lenth
Northing (km)
Easting (km)
Home Range Area (km2)
Density

(a)

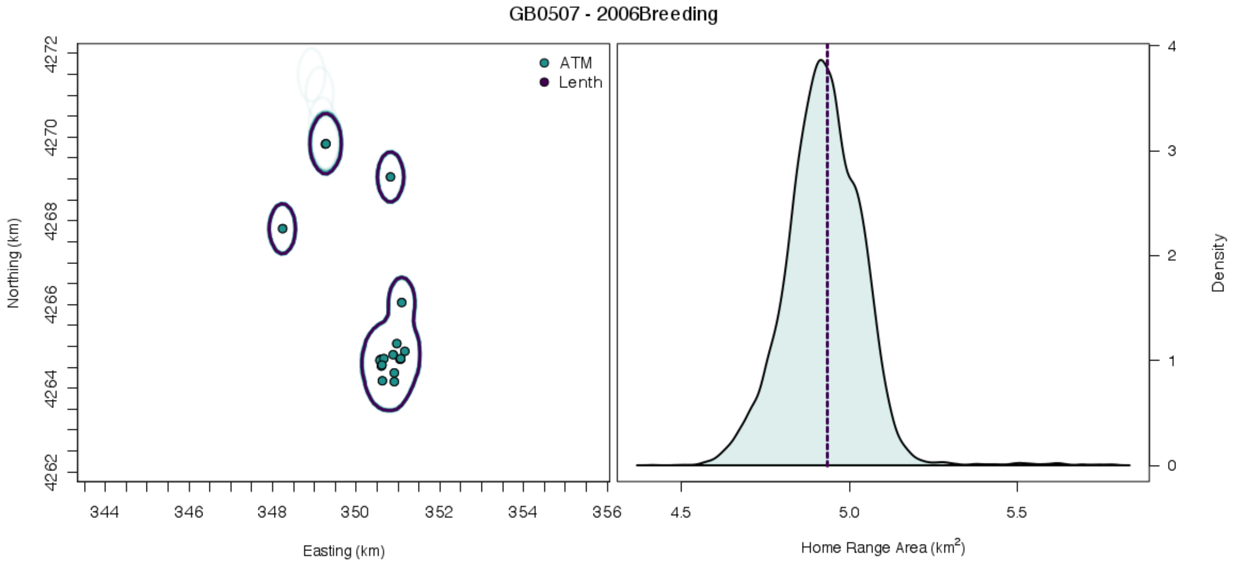
GB0507 - 2006Breeding
ATM
Lenth
Northing (km)
Easting (km)
Home Range Area (km2)
Density

(b)

Figure 14

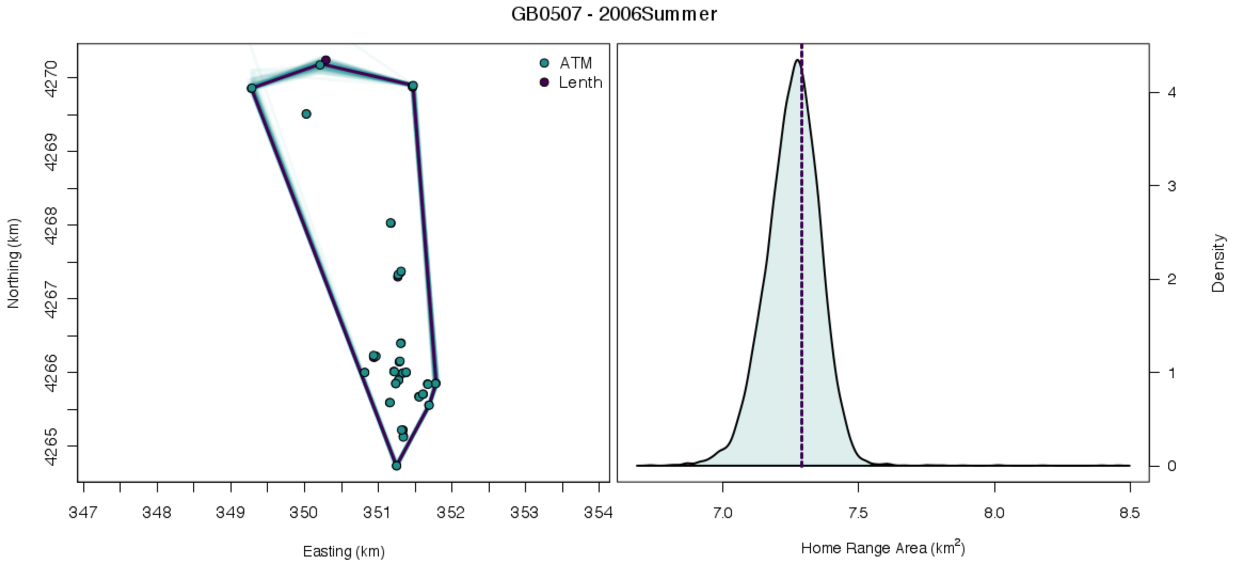
GB0507 - 2006Summer
ATM
Lenth
Northing (km)
Easting (km)
Density
Home Range Area (km²)

(a)

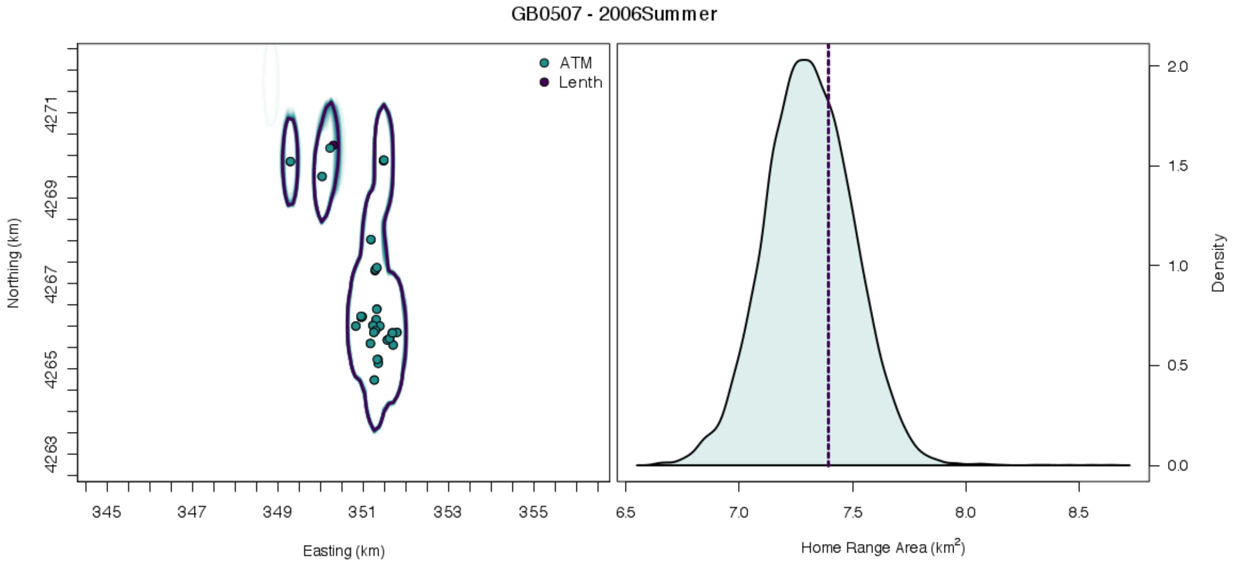
GB0507 - 2006Summer
ATM
Lenth
Northing (km)
Easting (km)
Density
Home Range Area (km²)

(b)

Figure 15

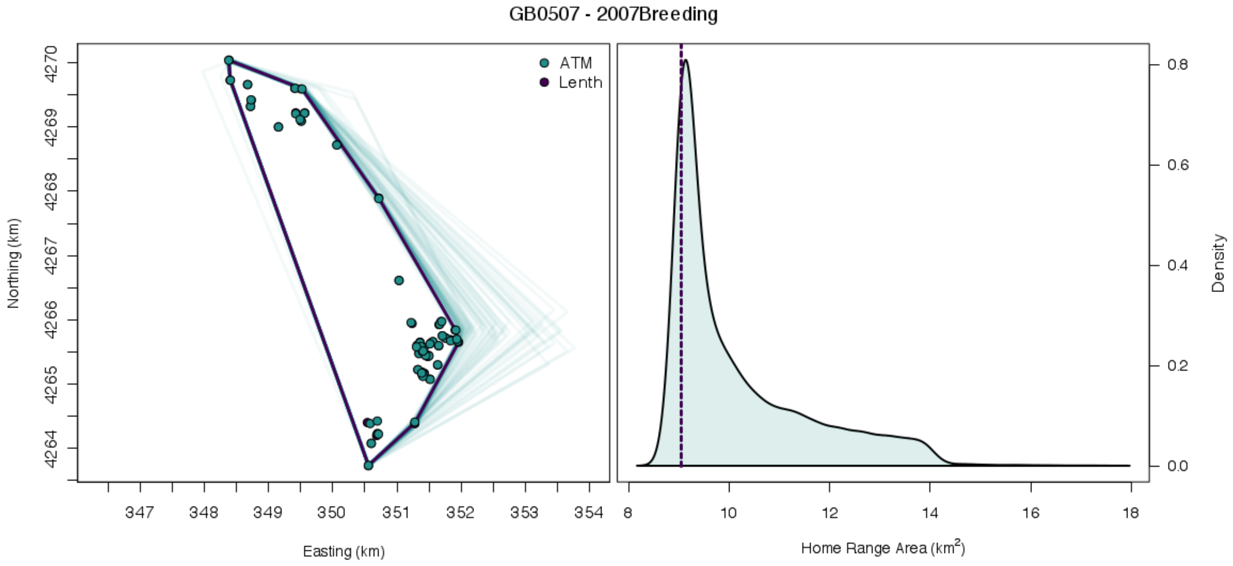
GB0507 - 2007Breeding
ATM
Lenth
Northing (km)
Easting (km)
Home Range Area (km^2)
Density

(a)

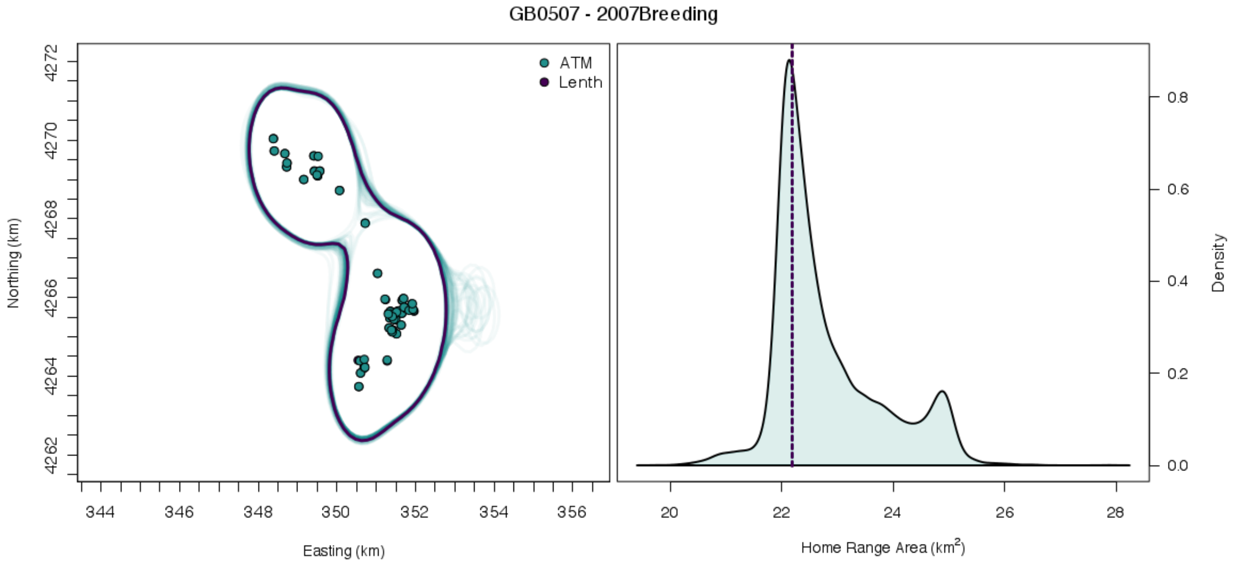
GB0507 - 2007Breeding
ATM
Lenth
Northing (km)
Easting (km)
Home Range Area (km^2)
Density

(b)

Figure 16

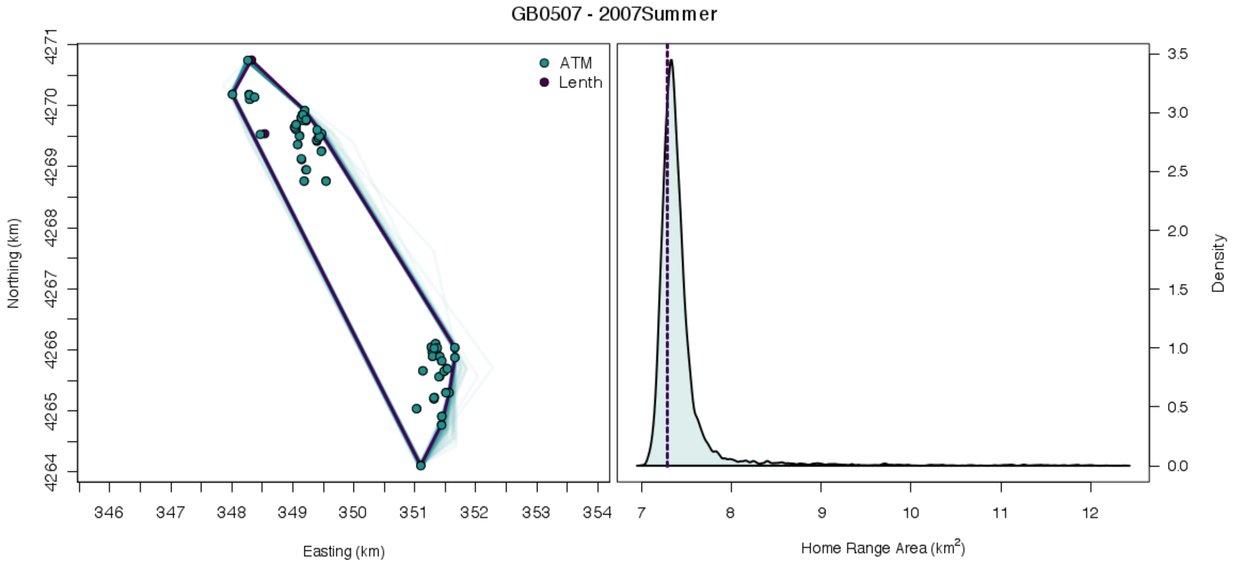
GB0507 - 2007Summer
ATM
Lenth
Northing (km)
Easting (km)
Density
Home Range Area (km²)

(a)

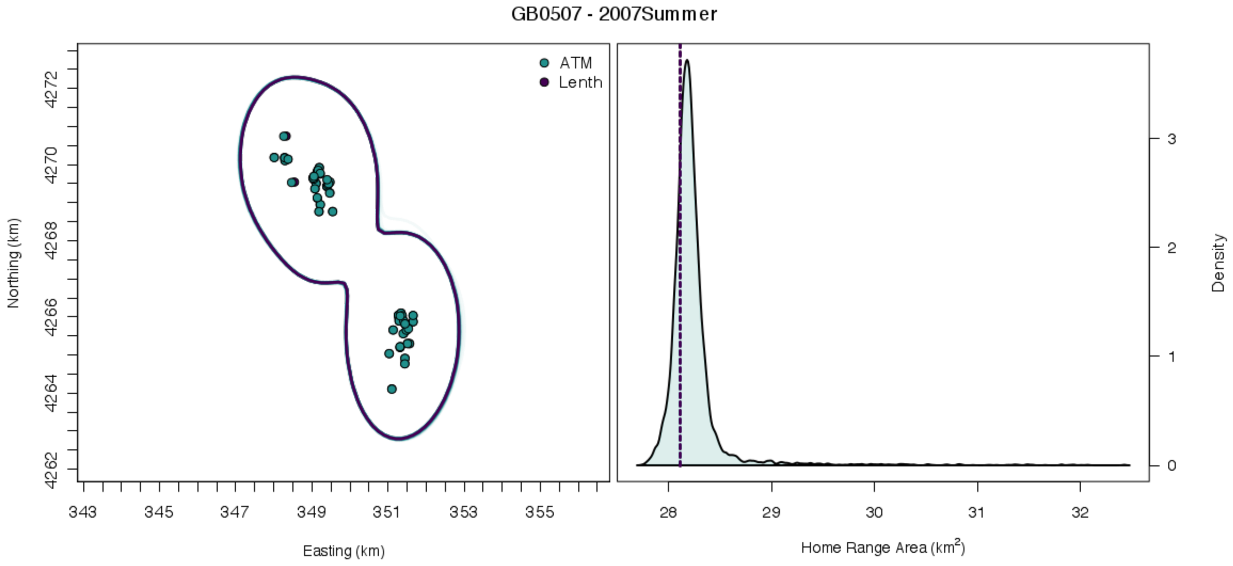
GB0507 - 2007Summer
ATM
Lenth
Northing (km)
Easting (km)
Density
Home Range Area (km²)

(b)

Figure 17

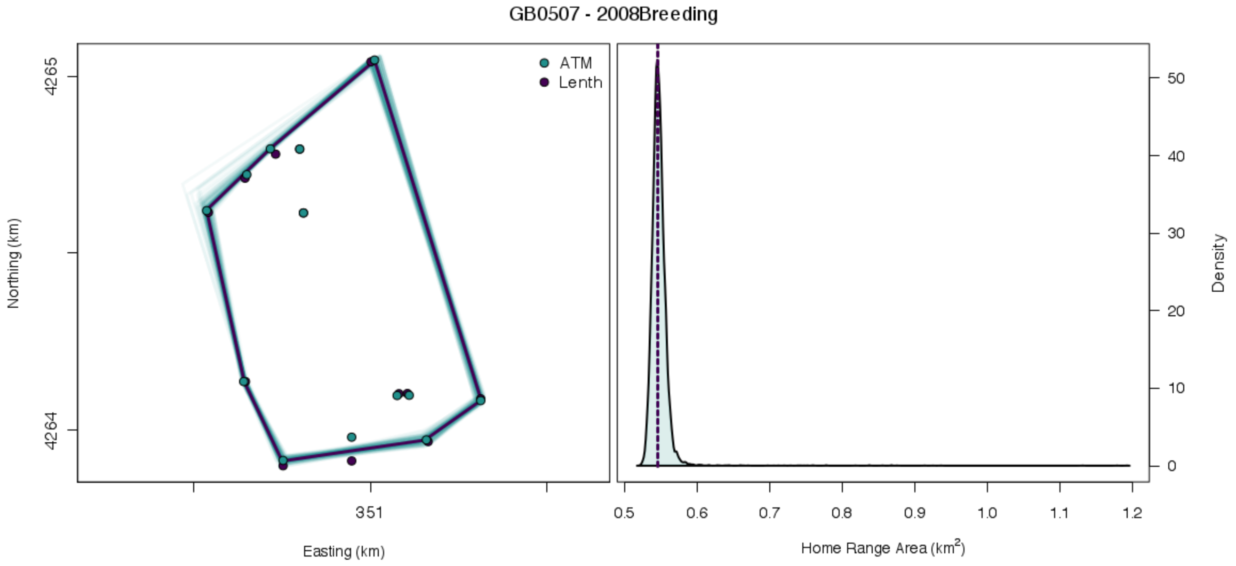
GB0507 - 2008Breeding
ATM
Lenth
Northing (km)
4265
4264
351
Easting (km)
0.5
0.6
0.7
0.8
0.9
1.0
1.1
1.2
Home Range Area (km^2)
50
40
30
20
10
0
Density

(a)

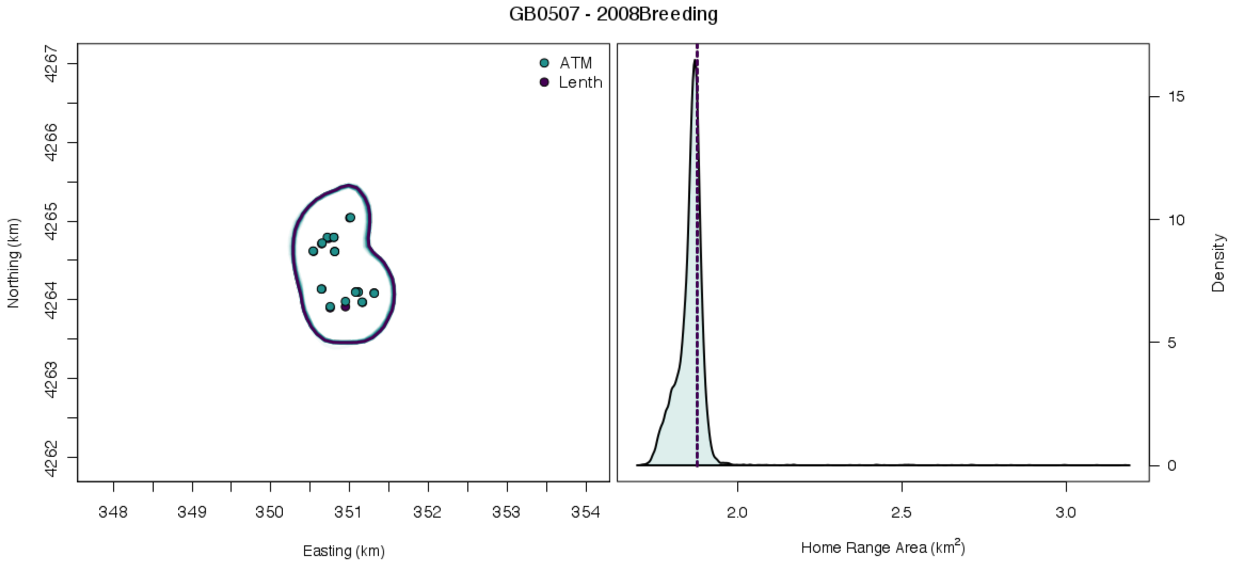
GB0507 - 2008Breeding
ATM
Lenth
Northing (km)
4267
4266
4265
4264
4263
4262
348
349
350
351
352
353
354
Easting (km)
2.0
2.5
3.0
Home Range Area (km^2)
15
10
5
0
Density

(b)

Figure 18

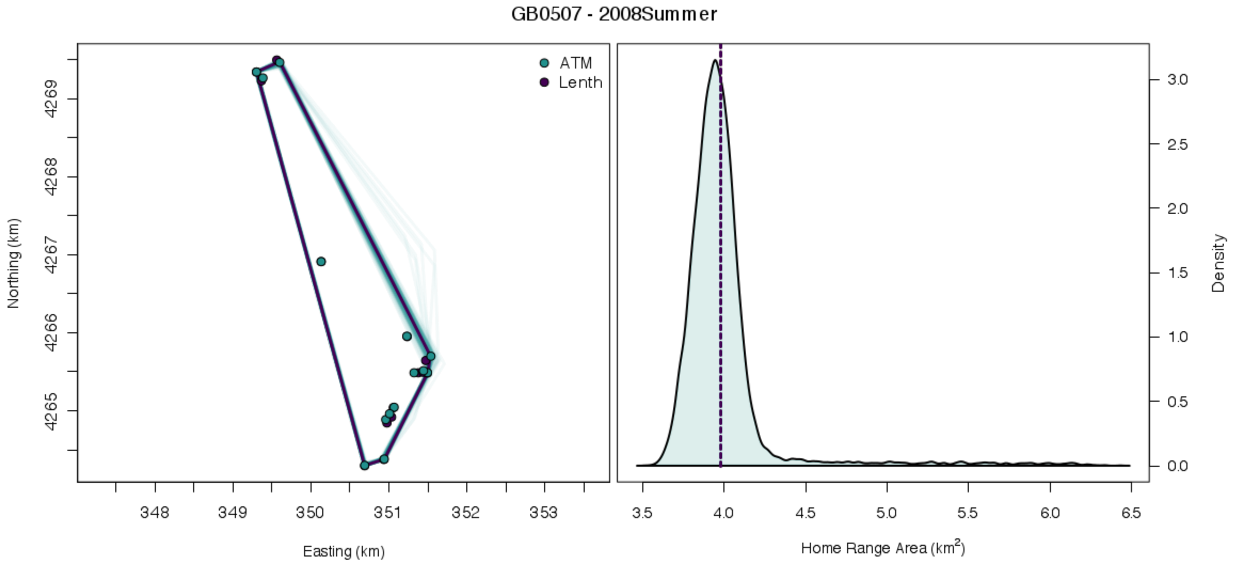
GB0507 - 2008Summer
ATM
Lenth
Northing (km)
Easting (km)
Density
Home Range Area (km^2)

(a)

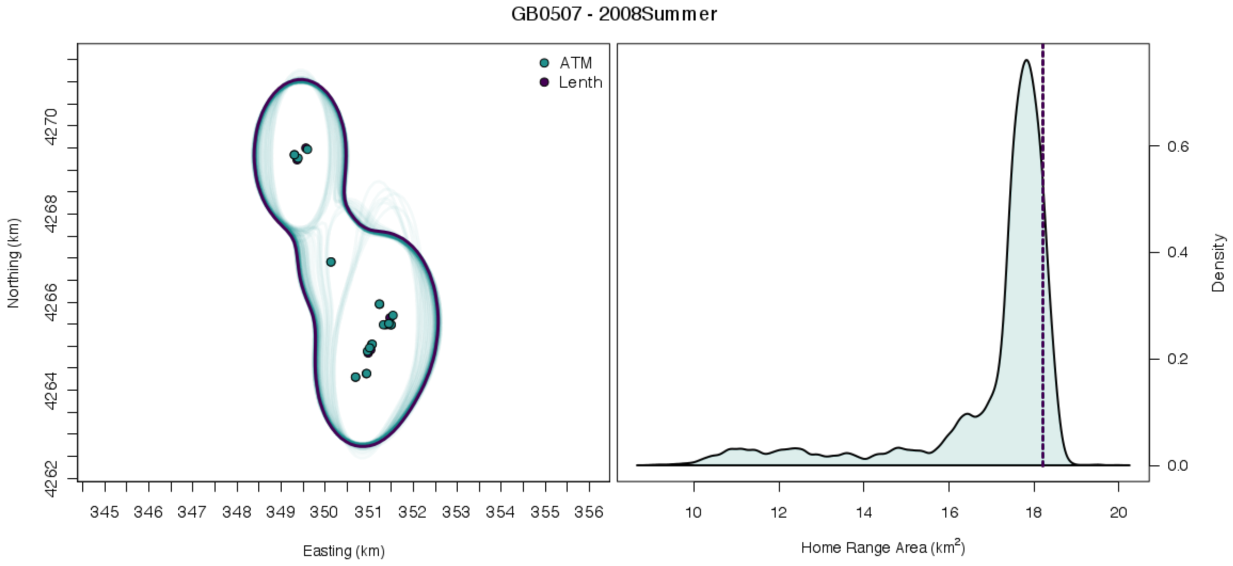
GB0507 - 2008Summer
ATM
Lenth
Northing (km)
Easting (km)
Density
Home Range Area (km^2)

(b)

Figure 19

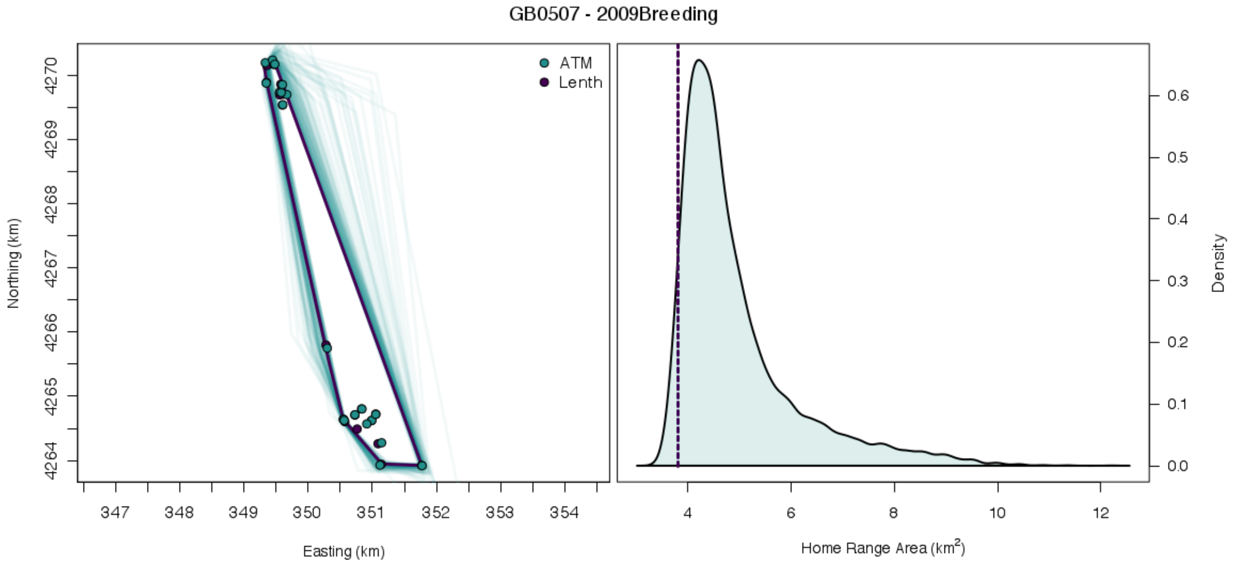
GB0507 - 2009Breeding
ATM
Lenth
Northing (km)
Easting (km)
Home Range Area (km²)
Density

(a)

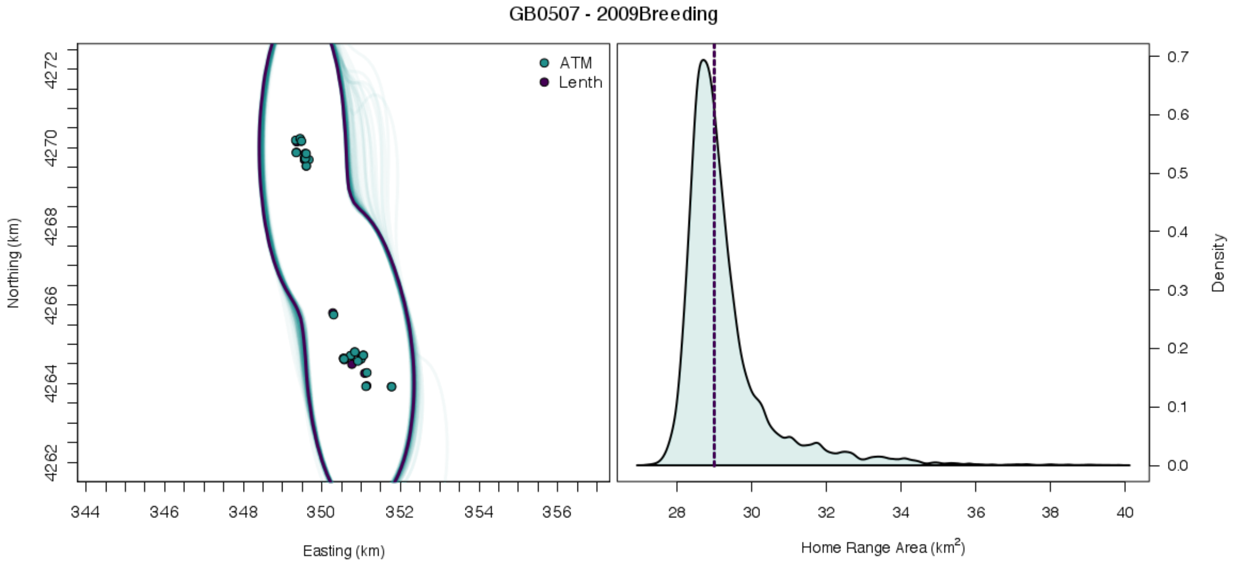
GB0507 - 2009Breeding
ATM
Lenth
Northing (km)
Easting (km)
Home Range Area (km²)
Density

(b)

Figure 20

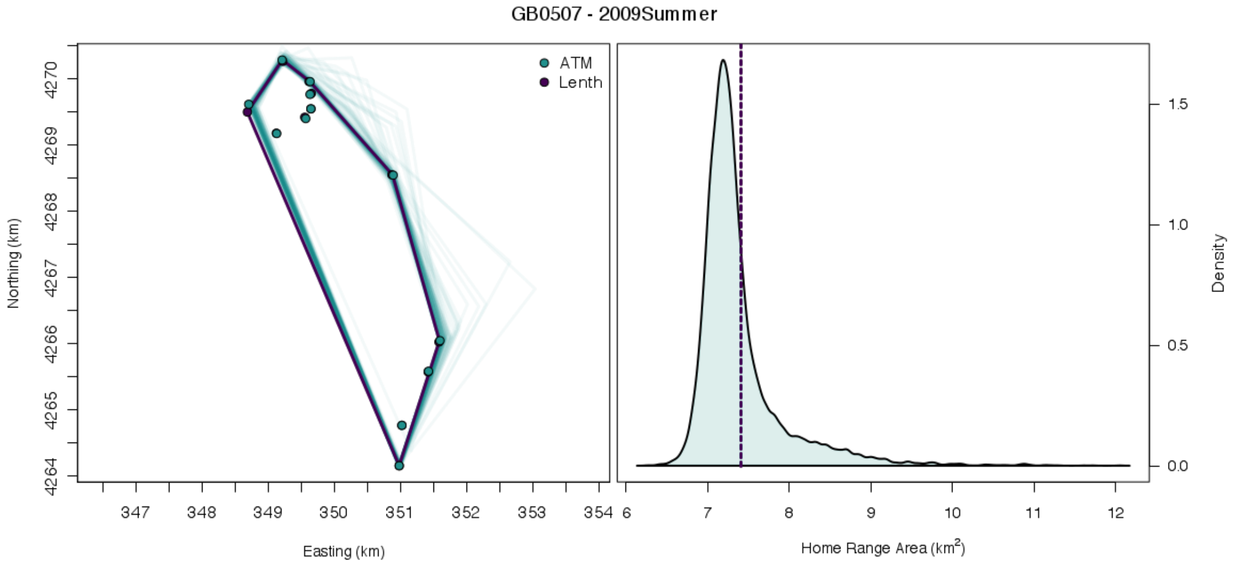
GB0507 - 2009Summer
ATM
Lenth
Northing (km)
Easting (km)
Home Range Area (km^2)
Density

(a)

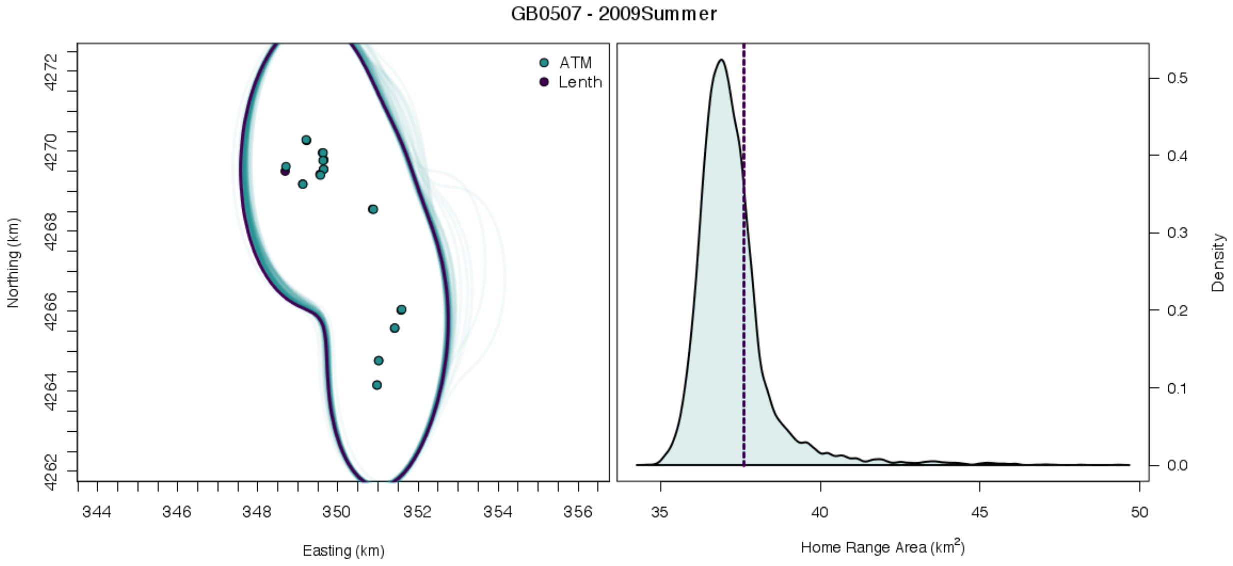
GB0507 - 2009Summer
ATM
Lenth
Northing (km)
Easting (km)
Home Range Area (km^2)
Density

(b)

Figure 21

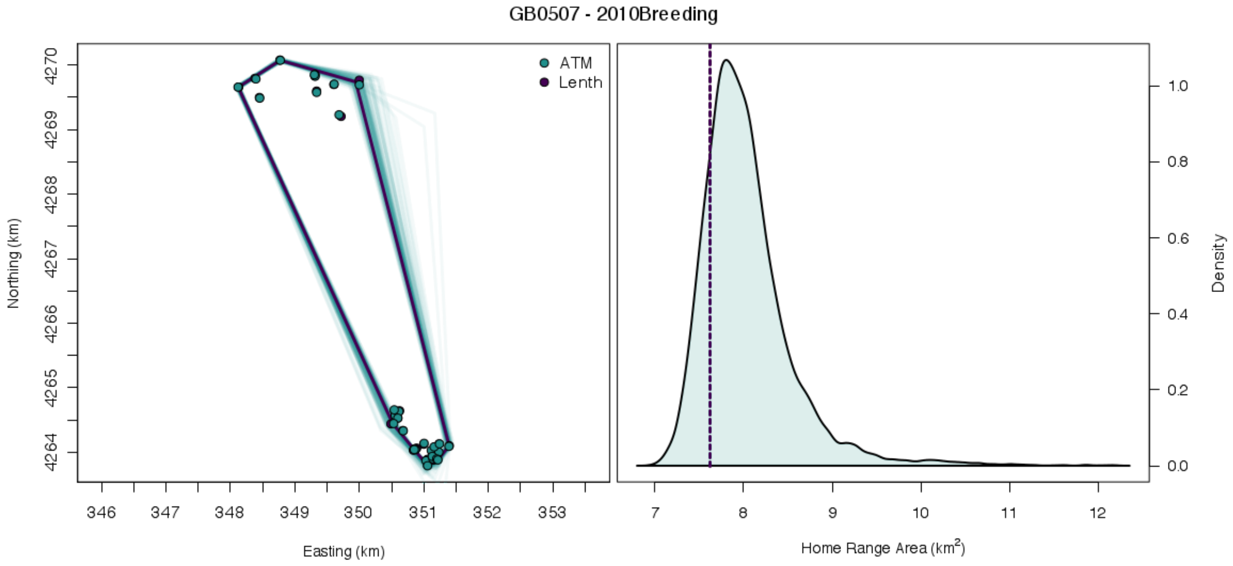
GB0507 - 2010Breeding
ATM
Lenth
Northing (km)
Easting (km)
Home Range Area (km^2)
Density

(a)

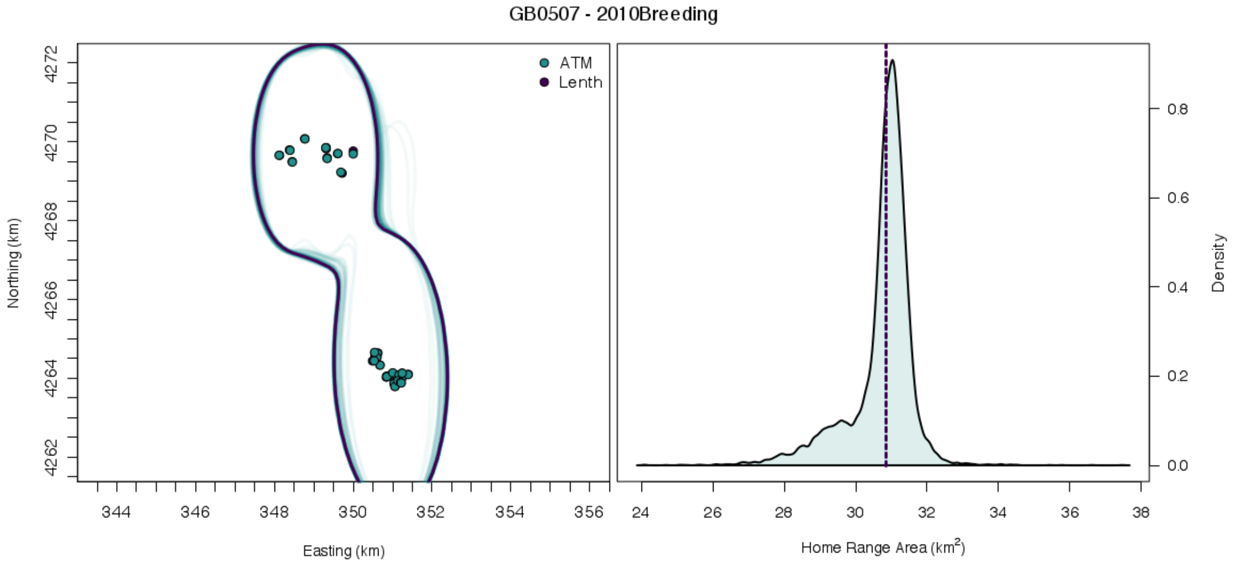
GB0507 - 2010Breeding
ATM
Lenth
Northing (km)
Easting (km)
Home Range Area (km^2)
Density

(b)

Figure 22

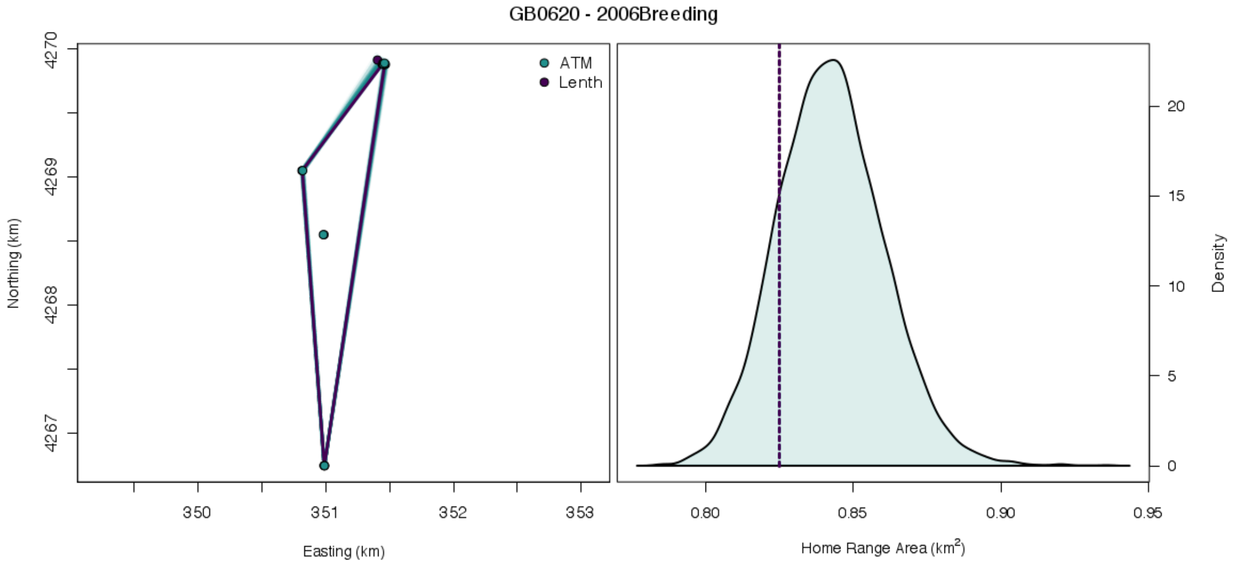
GB0620 - 2006Breeding
ATM
Lenth
Northing (km)
Easting (km)
Home Range Area (km²)
Density

(a)

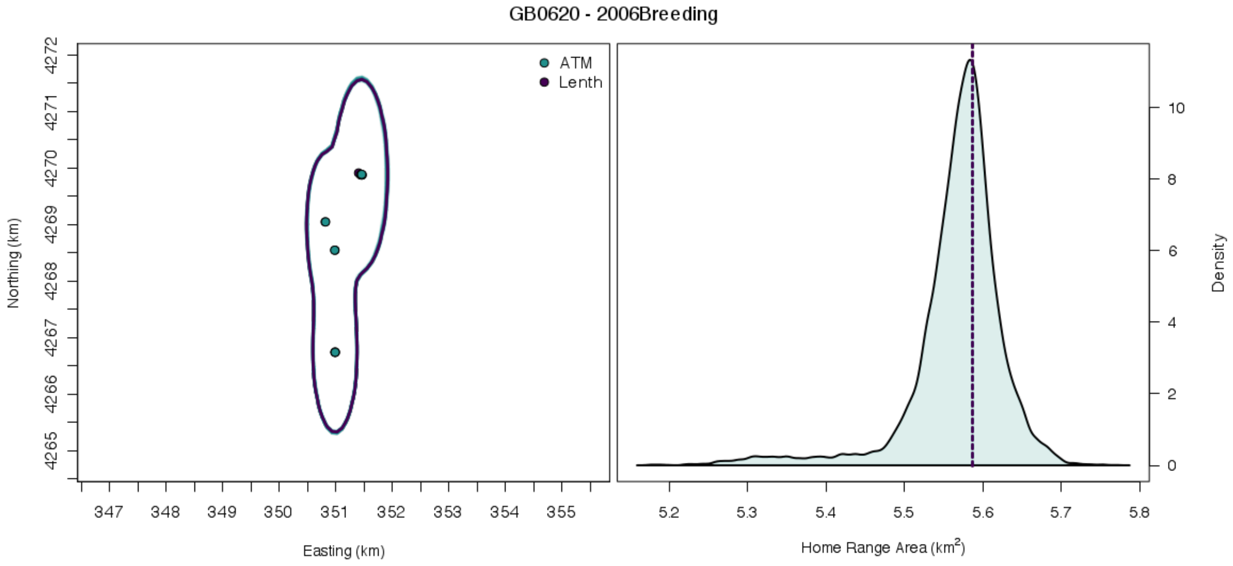
GB0620 - 2006Breeding
ATM
Lenth
Northing (km)
Easting (km)
Home Range Area (km²)
Density

(b)

Figure 23

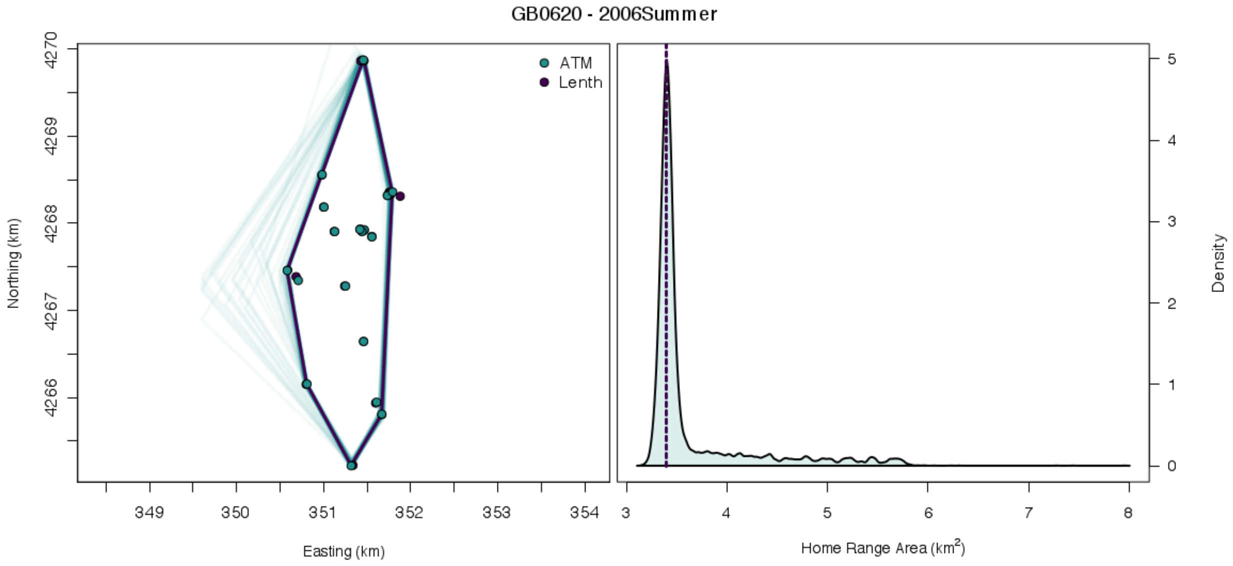
GB0620 - 2006Summer
ATM
Lenth
Northing (km)
Easting (km)
Home Range Area (km^2)
Density

(a)

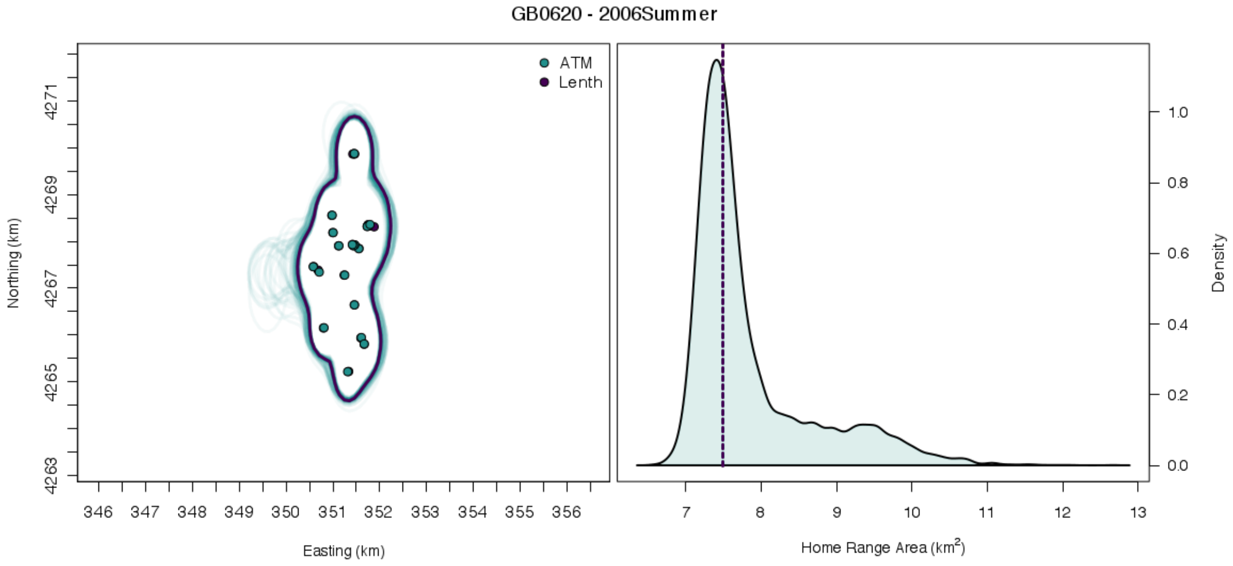
GB0620 - 2006Summer
ATM
Lenth
Northing (km)
Easting (km)
Home Range Area (km^2)
Density

(b)

Figure 24

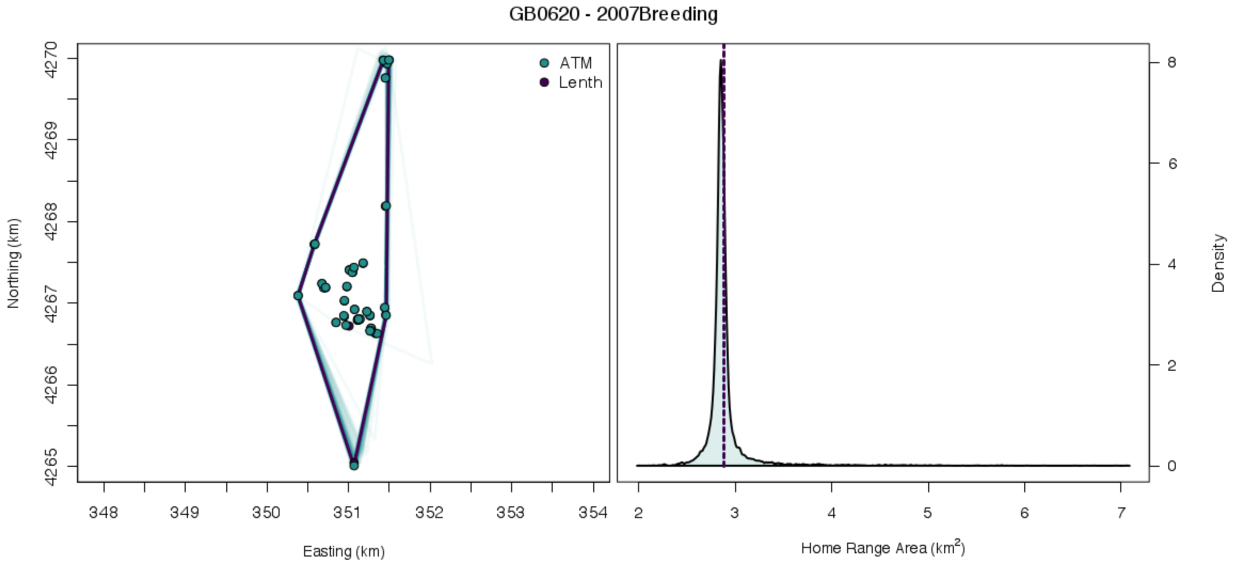
GB0620 - 2007Breeding
ATM
Lenth
Northing (km)
Easting (km)
Home Range Area (km^2)
Density

(a)

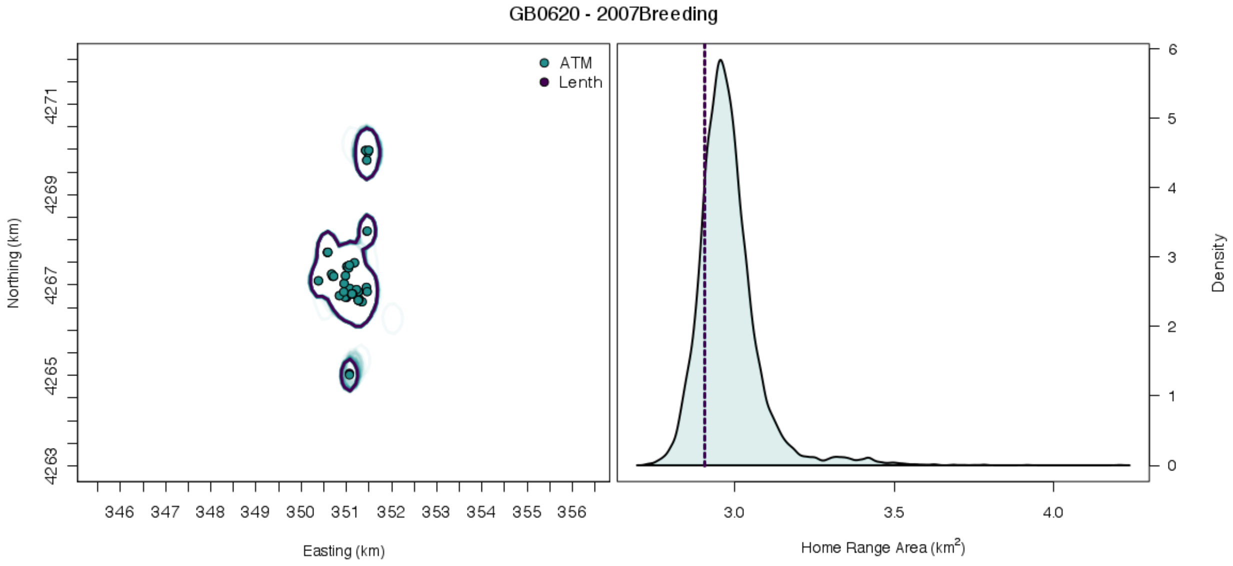
GB0620 - 2007Breeding
ATM
Lenth
Northing (km)
Easting (km)
Home Range Area (km^2)
Density

(b)

Figure 25

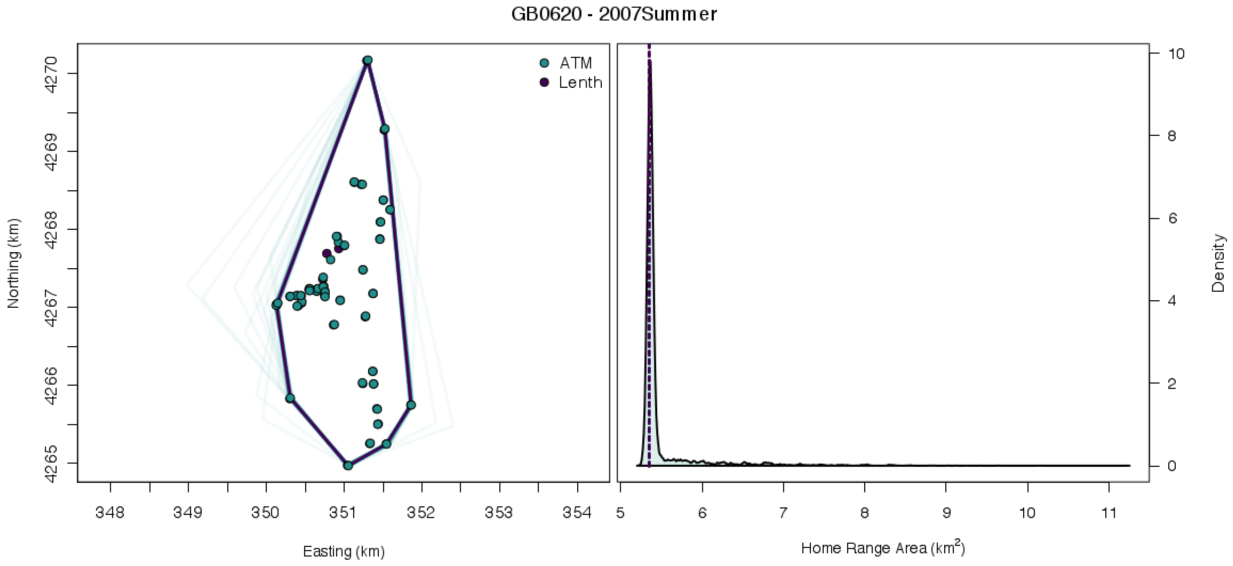
GB0620 - 2007Summer
ATM
Lenth
Northing (km)
Easting (km)
Density
Home Range Area (km²)

(a)

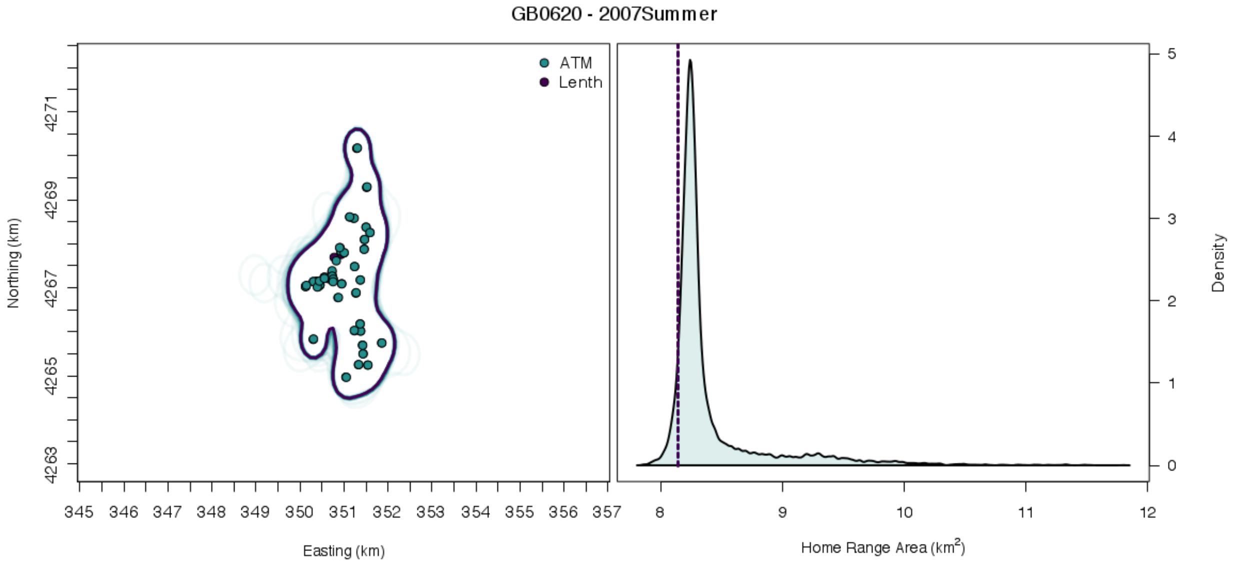
GB0620 - 2007Summer
ATM
Lenth
Northing (km)
Easting (km)
Density
Home Range Area (km²)

(b)

Figure 26

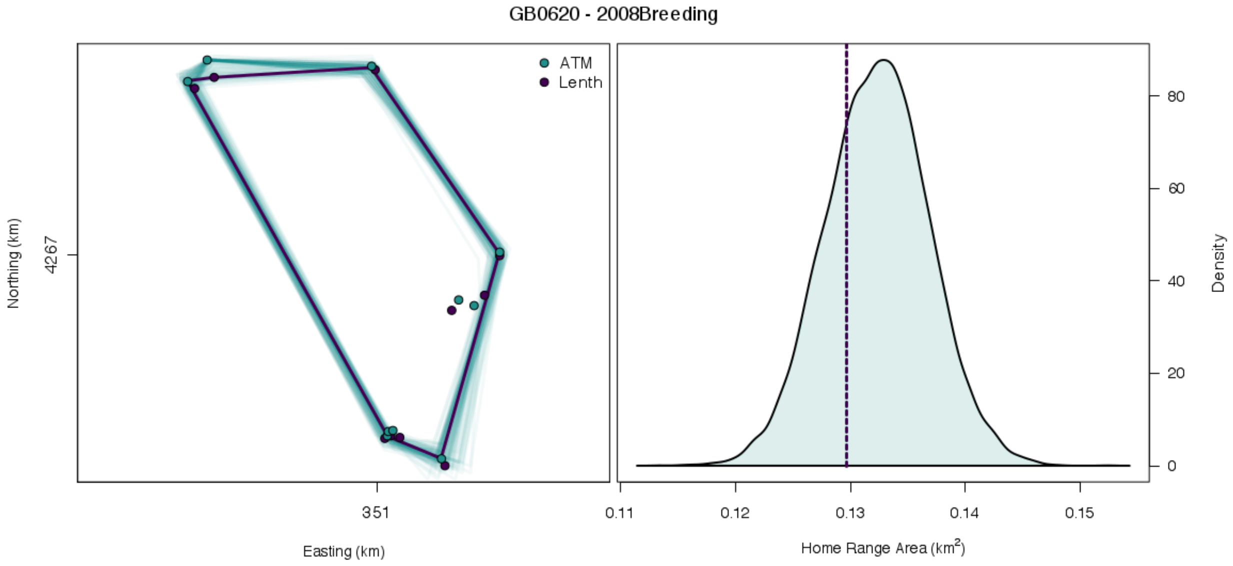
GB0620 - 2008Breeding
ATM
Lenth
Northing (km)
4267
351
Easting (km)
0.11
0.12
0.13
0.14
0.15
Home Range Area (km^2)
0
20
40
60
80
Density

(a)

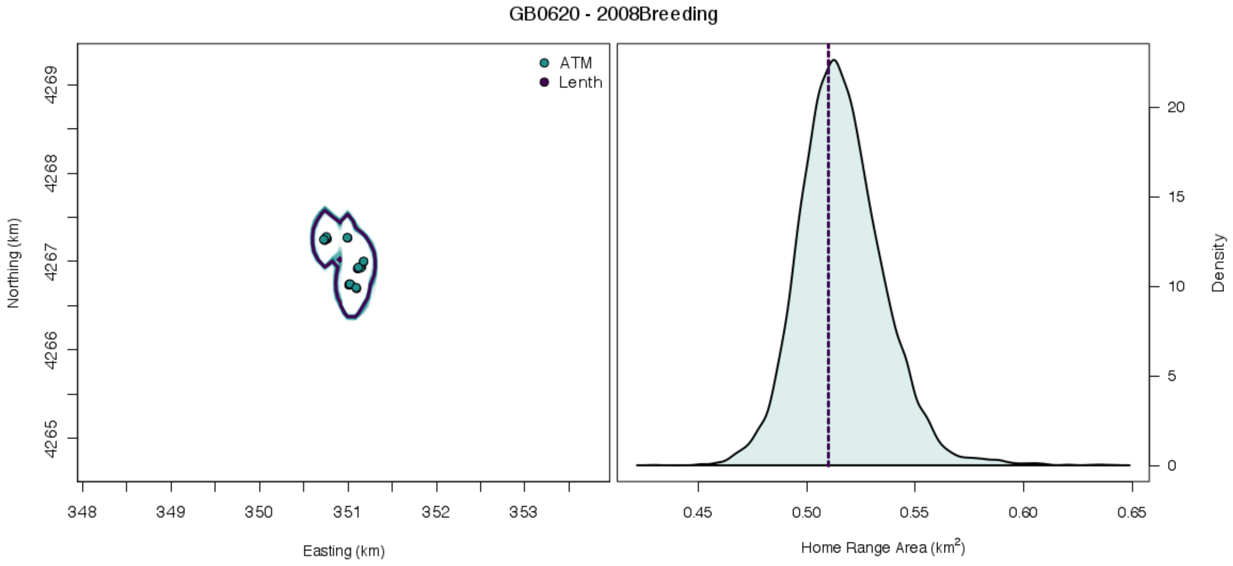
GB0620 - 2008Breeding
ATM
Lenth
Northing (km)
4265
4266
4267
4268
4269
348
349
350
351
352
353
Easting (km)
0.45
0.50
0.55
0.60
0.65
Home Range Area (km^2)
0
5
10
15
20
Density

(b)

Figure 27

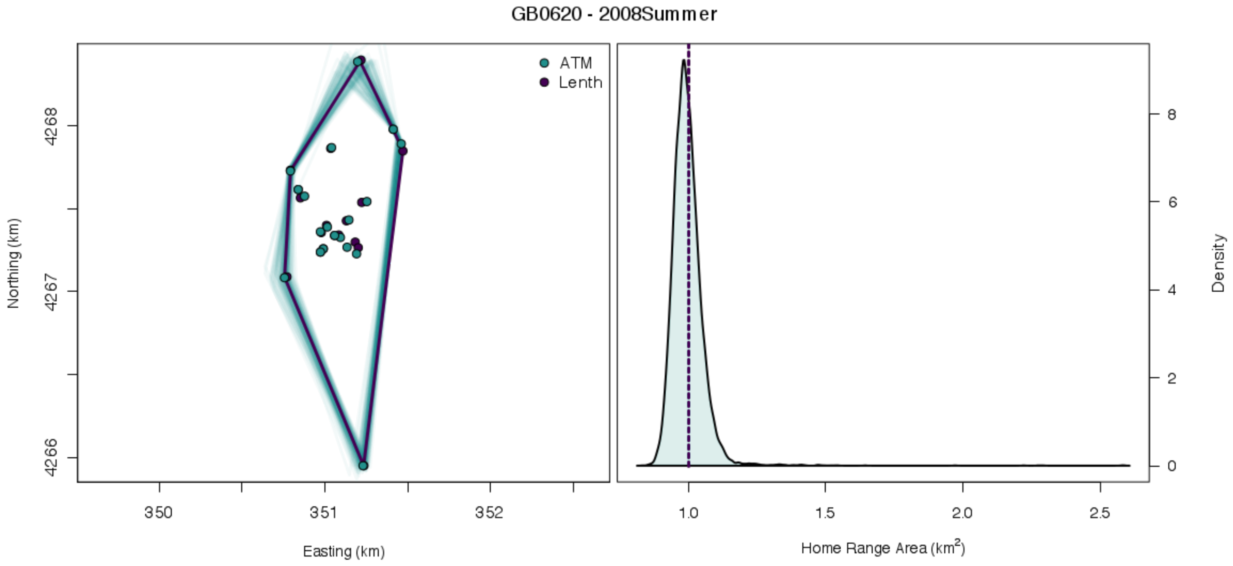
GB0620 - 2008Summer
ATM
Lenth
Northing (km)
4266
4267
4268
Easting (km)
350
351
352
Density
0
2
4
6
8
Home Range Area (km2)
1.0
1.5
2.0
2.5

(a)

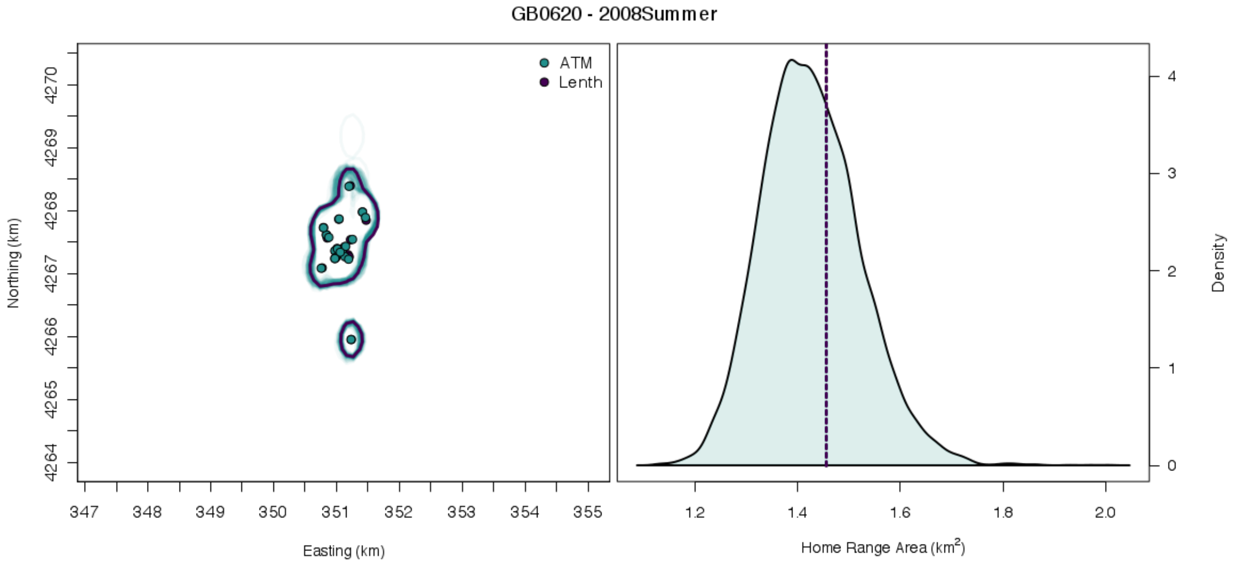
GB0620 - 2008Summer
ATM
Lenth
Northing (km)
4264
4265
4266
4267
4268
4269
4270
Easting (km)
347
348
349
350
351
352
353
354
355
Density
0
1
2
3
4
Home Range Area (km2)
1.2
1.4
1.6
1.8
2.0

(b)

Figure 28

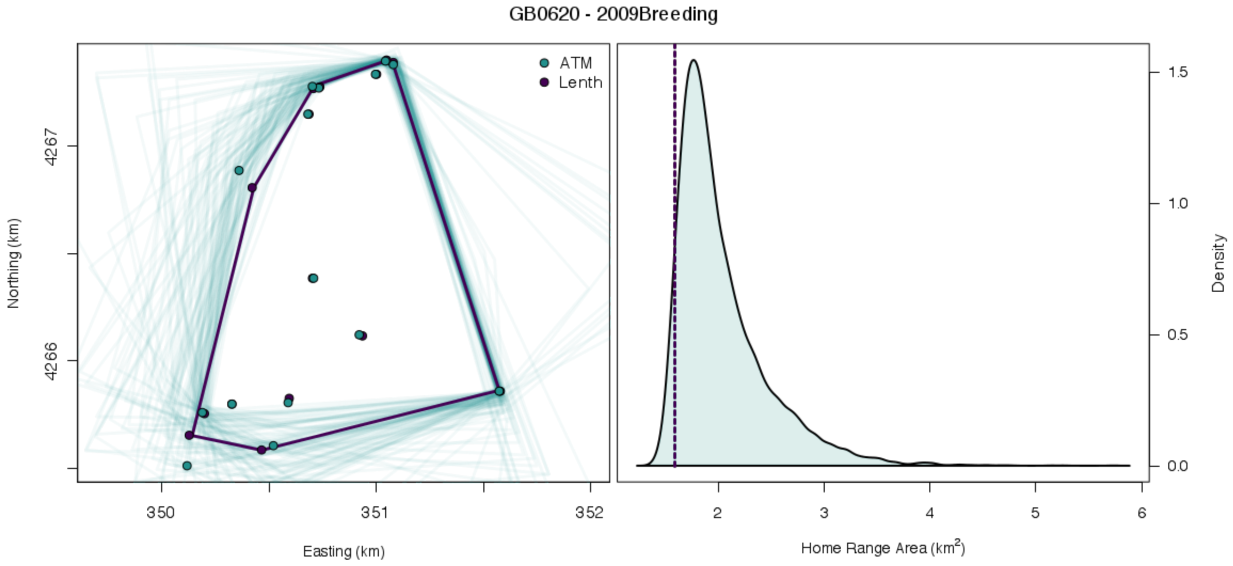
GB0620 - 2009Breeding
ATM
Lenth
Northing (km)
4267
4266
350
351
352
Easting (km)
1.5
1.0
0.5
0.0
Density
2
3
4
5
6
Home Range Area (km^2)

(a)

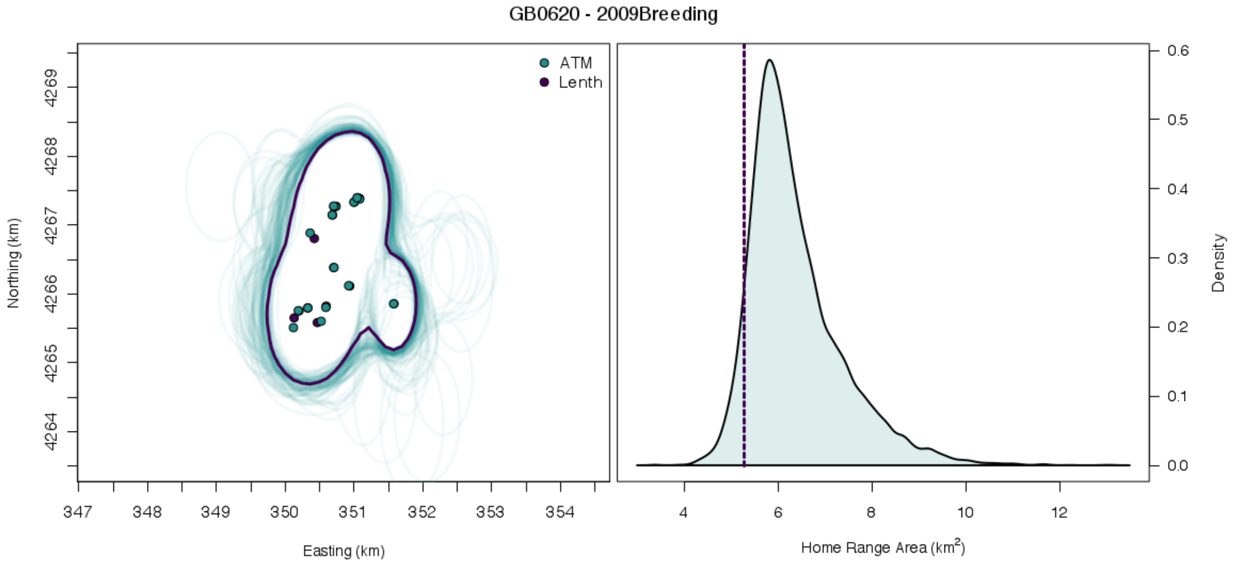
GB0620 - 2009Breeding
ATM
Lenth
Northing (km)
4269
4268
4267
4266
4265
4264
347
348
349
350
351
352
353
354
Easting (km)
0.6
0.5
0.4
0.3
0.2
0.1
0.0
Density
4
6
8
10
12
Home Range Area (km^2)

(b)

Figure 29

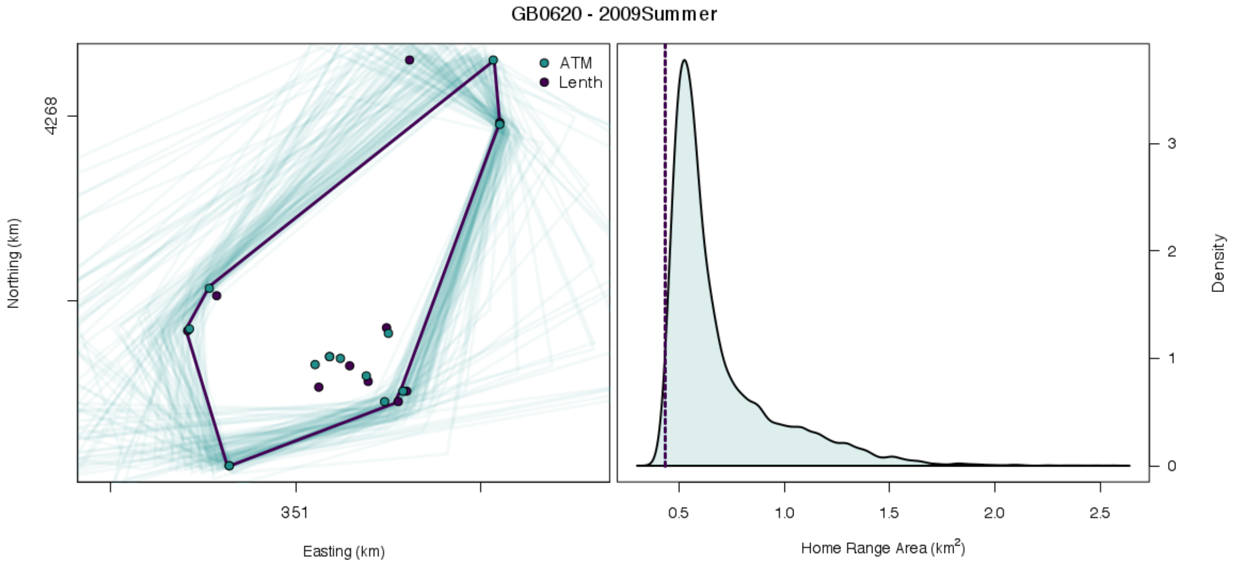
GB0620 - 2009Summer
ATM
Lenth
Northing (km)
4268
351
Easting (km)
0.5
1.0
1.5
2.0
2.5
Home Range Area (km^2)
Density
0
1
2
3

(a)

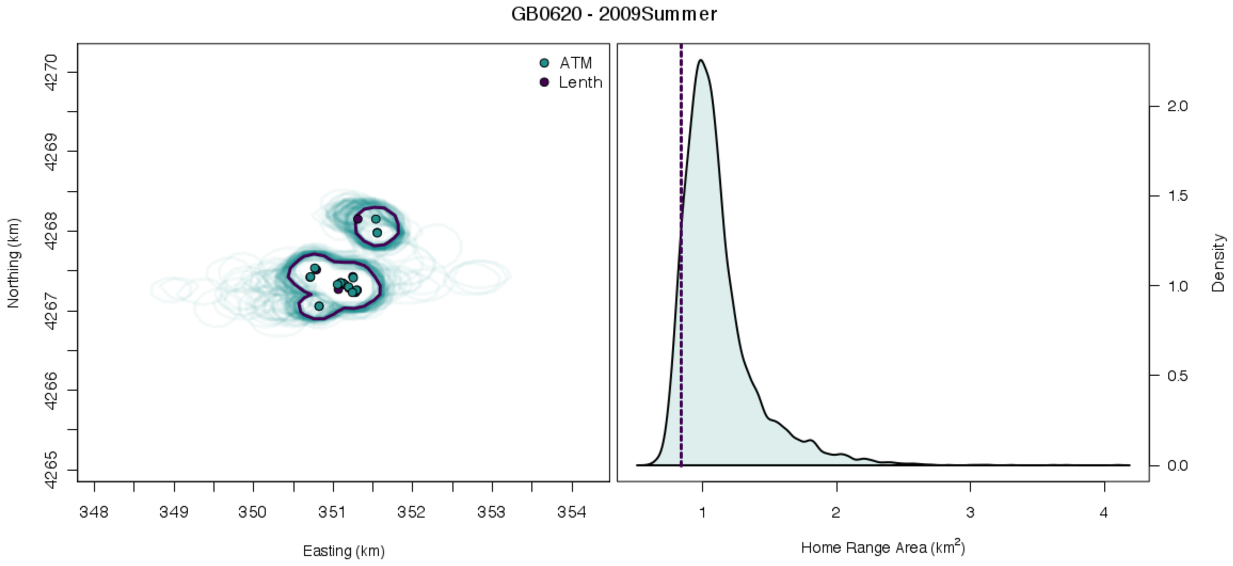
GB0620 - 2009Summer
ATM
Lenth
Northing (km)
4265
4266
4267
4268
4269
4270
348
349
350
351
352
353
354
Easting (km)
1
2
3
4
Home Range Area (km^2)
Density
0.0
0.5
1.0
1.5
2.0

(b)

Figure 30

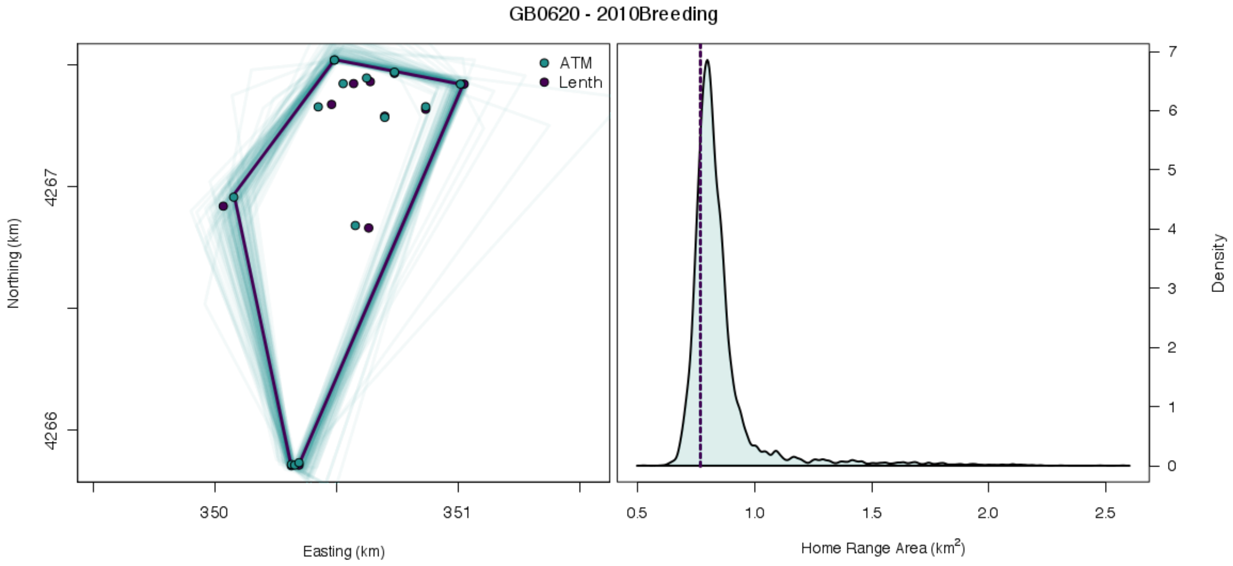
GB0620 - 2010Breeding
ATM
Lenth
Northing (km)
4266
4267
350
351
Easting (km)
0.5
1.0
1.5
2.0
2.5
Home Range Area (km^2)
Density
0
1
2
3
4
5
6
7

(a)

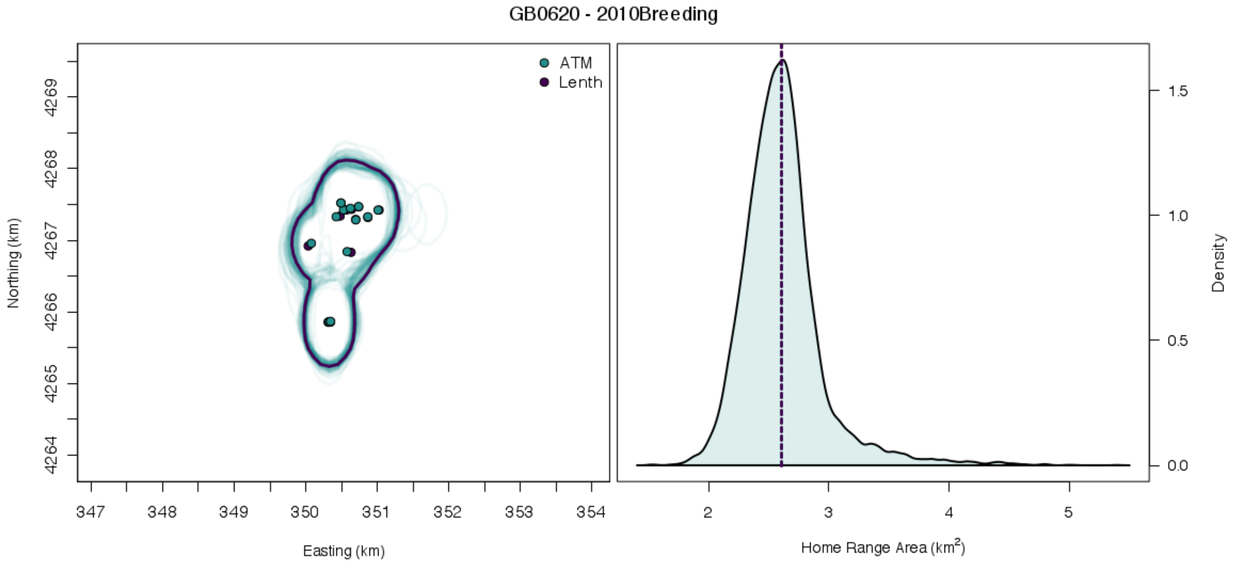
GB0620 - 2010Breeding
ATM
Lenth
Northing (km)
4264
4265
4266
4267
4268
4269
347
348
349
350
351
352
353
354
Easting (km)
2
3
4
5
Home Range Area (km^2)
Density
0.0
0.5
1.0
1.5

(b)

Figure 31

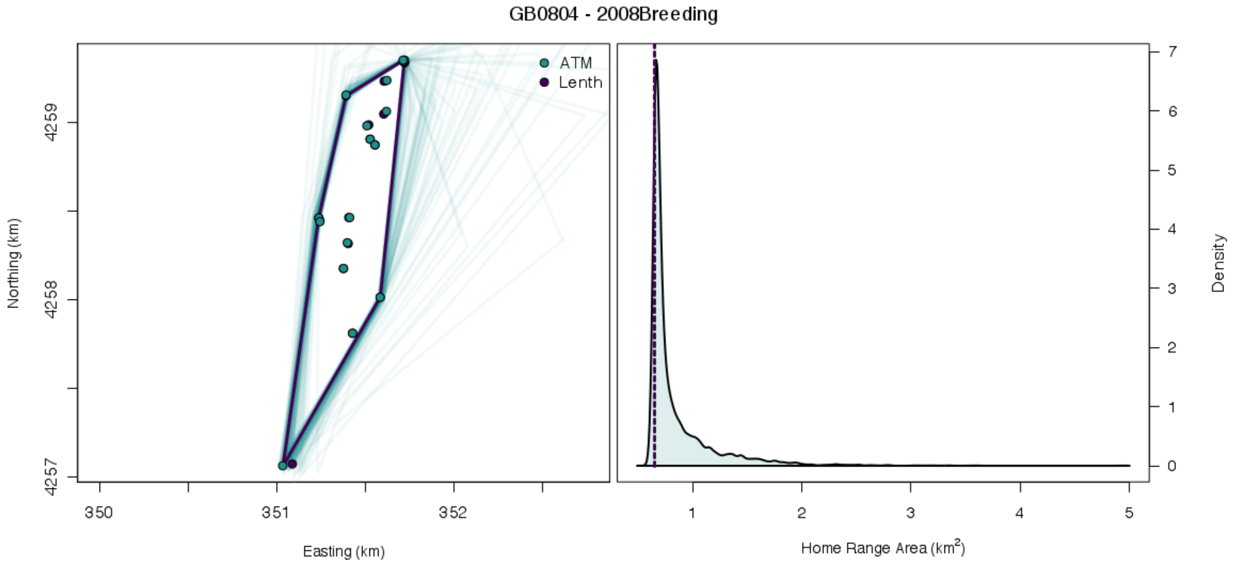

GB0804 - 2008Breeding
ATM
Lenth
Northing (km)
4257
4258
4259
350
351
352
Easting (km)
1
2
3
4
5
Home Range Area (km^2)
0
1
2
3
4
5
6
7
Density

(a)

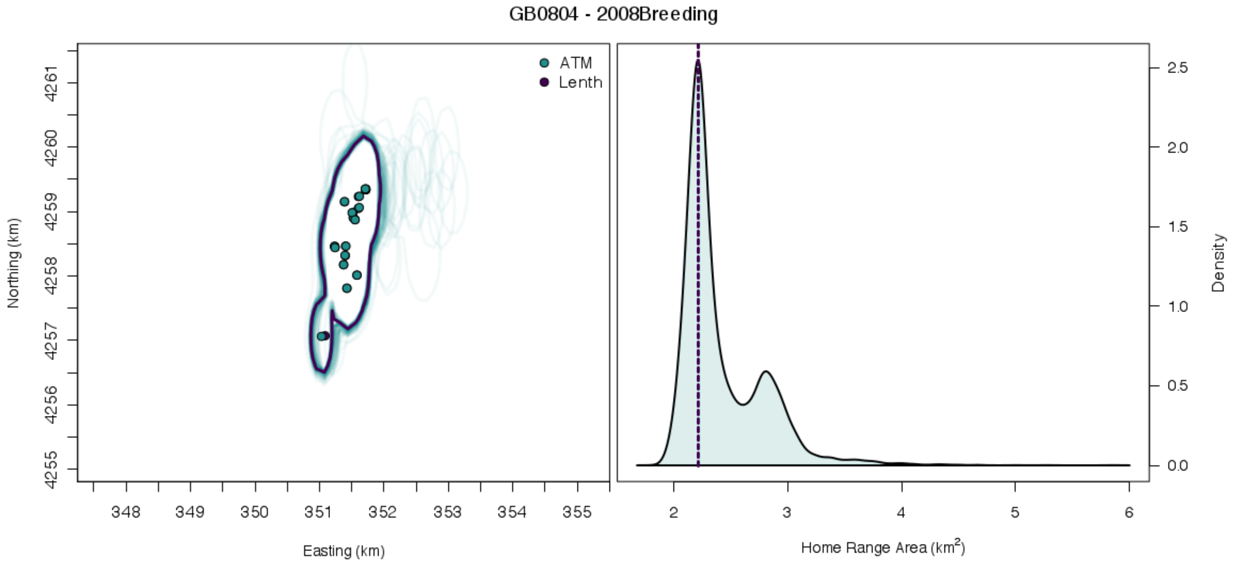

GB0804 - 2008Breeding
ATM
Lenth
Northing (km)
4255
4256
4257
4258
4259
4260
4261
348
349
350
351
352
353
354
355
Easting (km)
2
3
4
5
6
Home Range Area (km^2)
0.0
0.5
1.0
1.5
2.0
2.5
Density

(b)

Figure 32

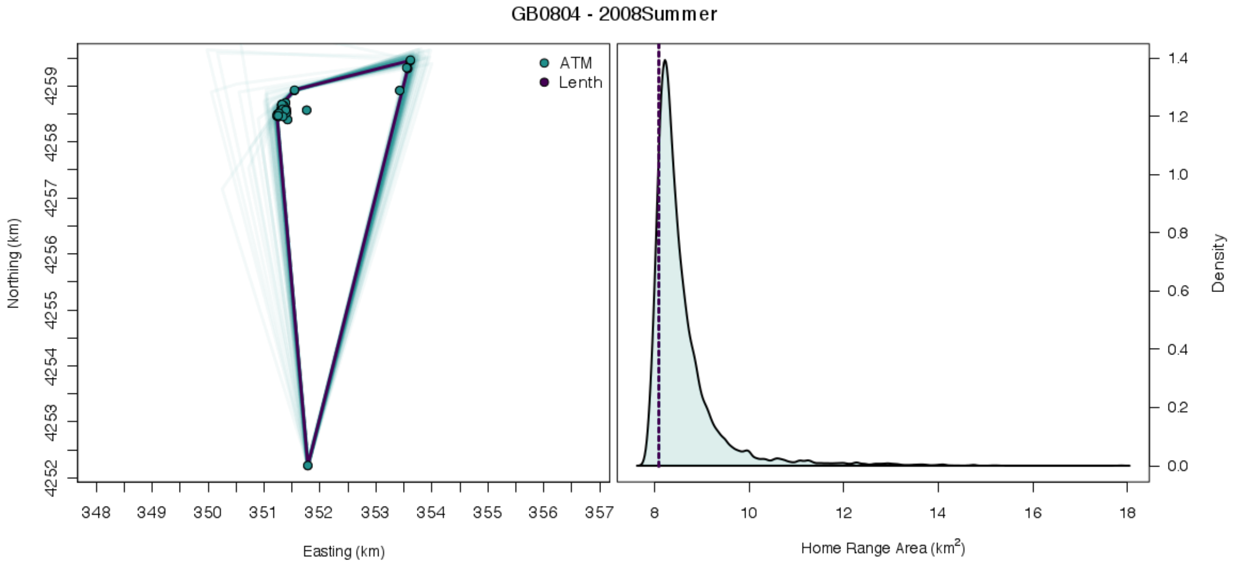
GB0804 - 2008Summer
ATM
Lenth
Northing (km)
Easting (km)
Home Range Area (km²)
Density

(a)

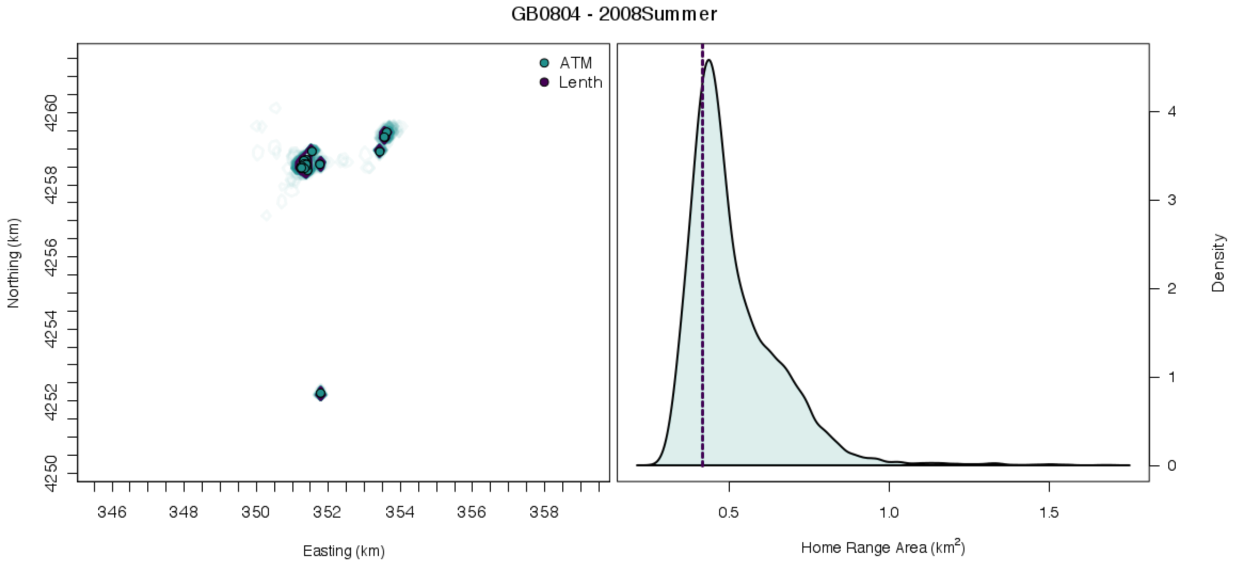
GB0804 - 2008Summer
ATM
Lenth
Northing (km)
Easting (km)
Home Range Area (km²)
Density

(b)

Figure 33

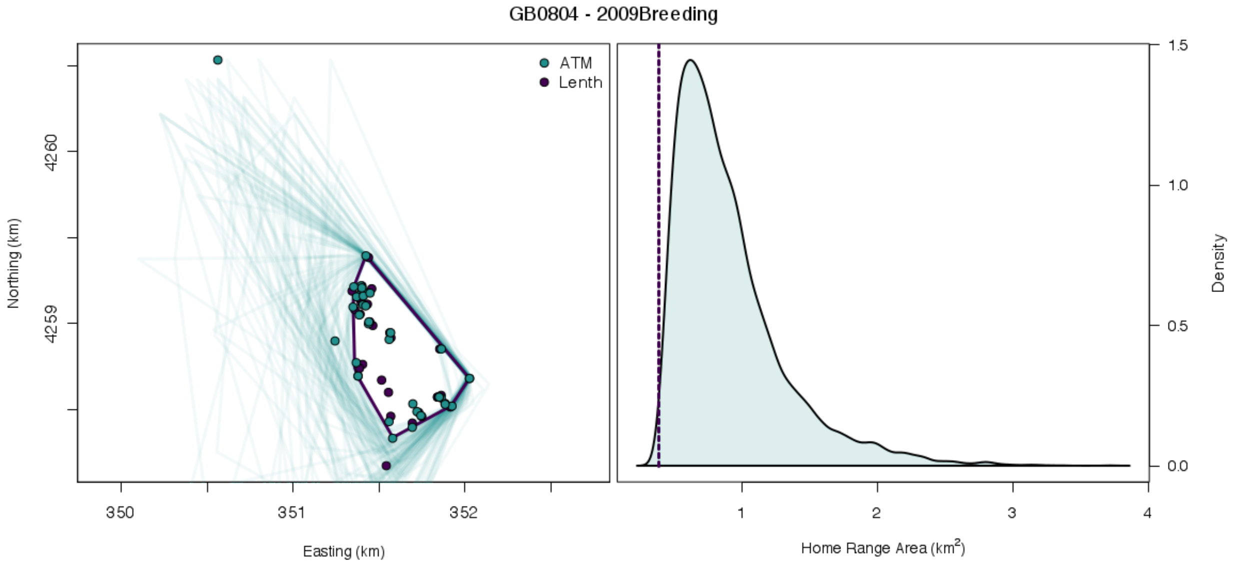
GB0804 - 2009Breeding
ATM
Lenth
Northing (km)
Easting (km)
Home Range Area (km²)
Density

(a)

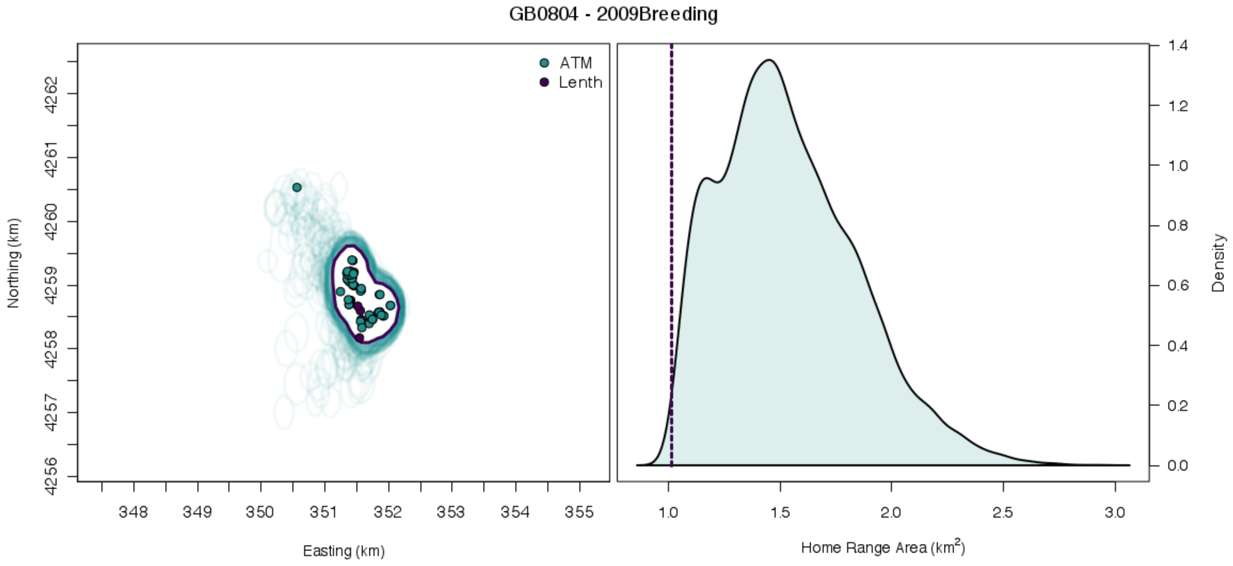
GB0804 - 2009Breeding
ATM
Lenth
Northing (km)
Easting (km)
Home Range Area (km²)
Density

(b)

Figure 34

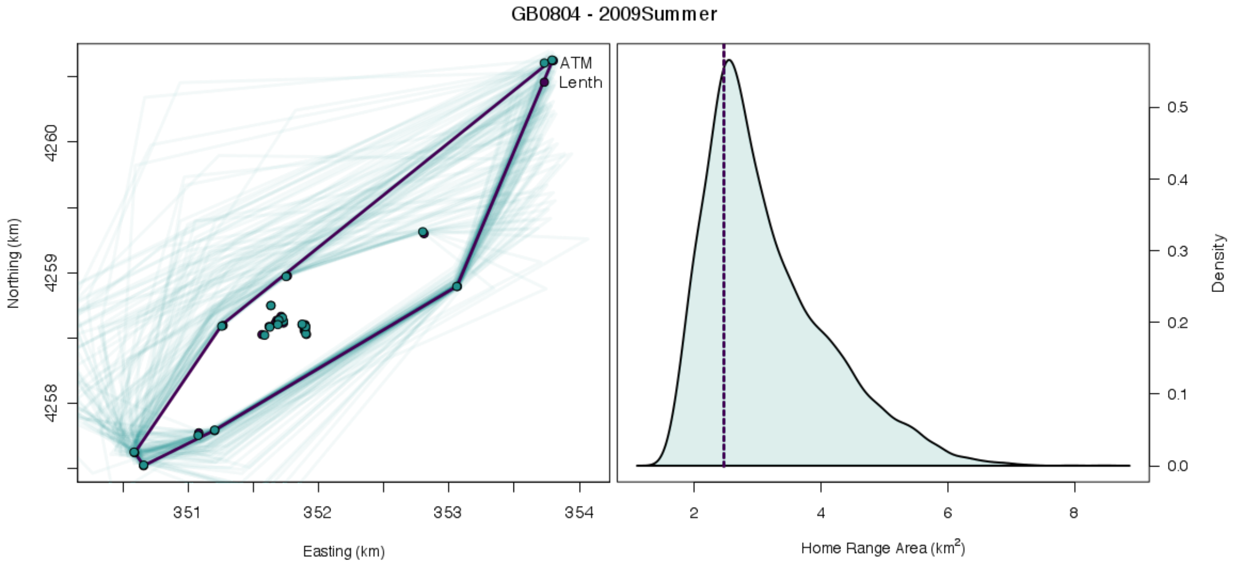
GB0804 - 2009Summer
ATM
Lenth
Northing (km)
4258
4259
4260
351
352
353
354
Easting (km)
Density
0.0
0.1
0.2
0.3
0.4
0.5
2
4
6
8
Home Range Area (km²)

(a)

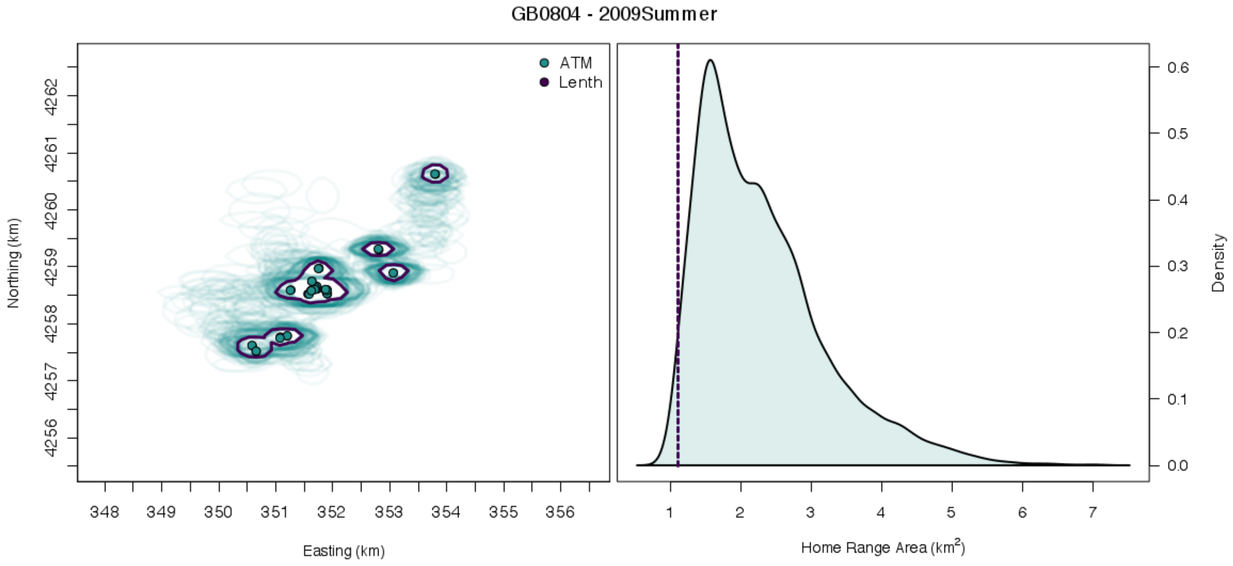
GB0804 - 2009Summer
ATM
Lenth
Northing (km)
4256
4257
4258
4259
4260
4261
4262
348
349
350
351
352
353
354
355
356
Easting (km)
Density
0.0
0.1
0.2
0.3
0.4
0.5
0.6
1
2
3
4
5
6
7
Home Range Area (km²)

(b)

Figure 35

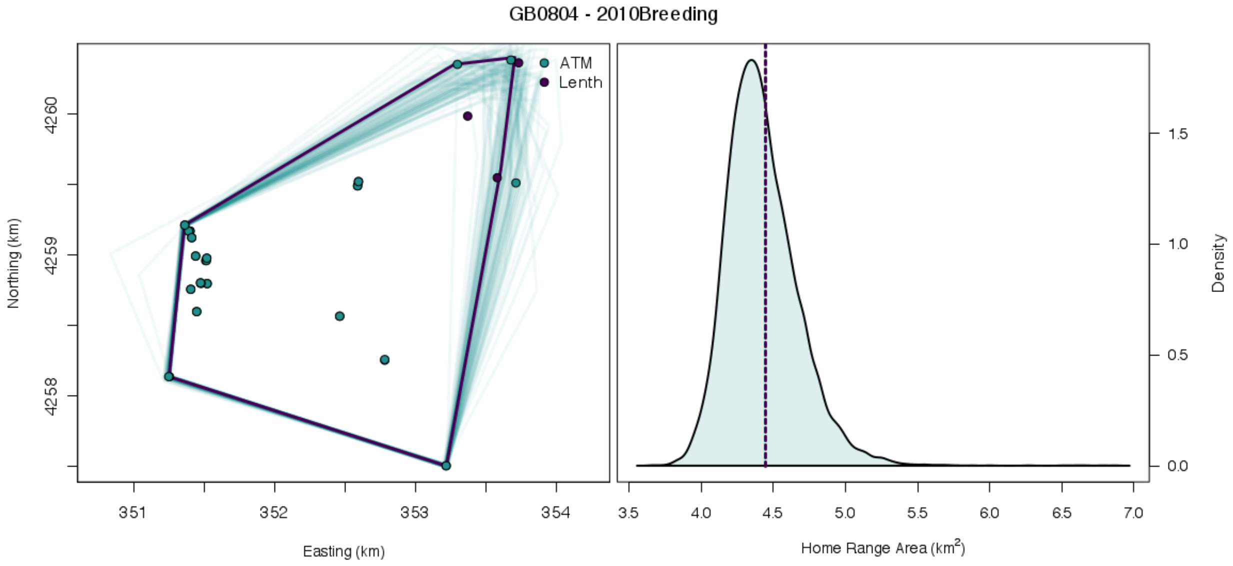
GB0804 - 2010Breeding
ATM
Lenth
Northing (km)
Easting (km)
Density
Home Range Area (km^2)

(a)

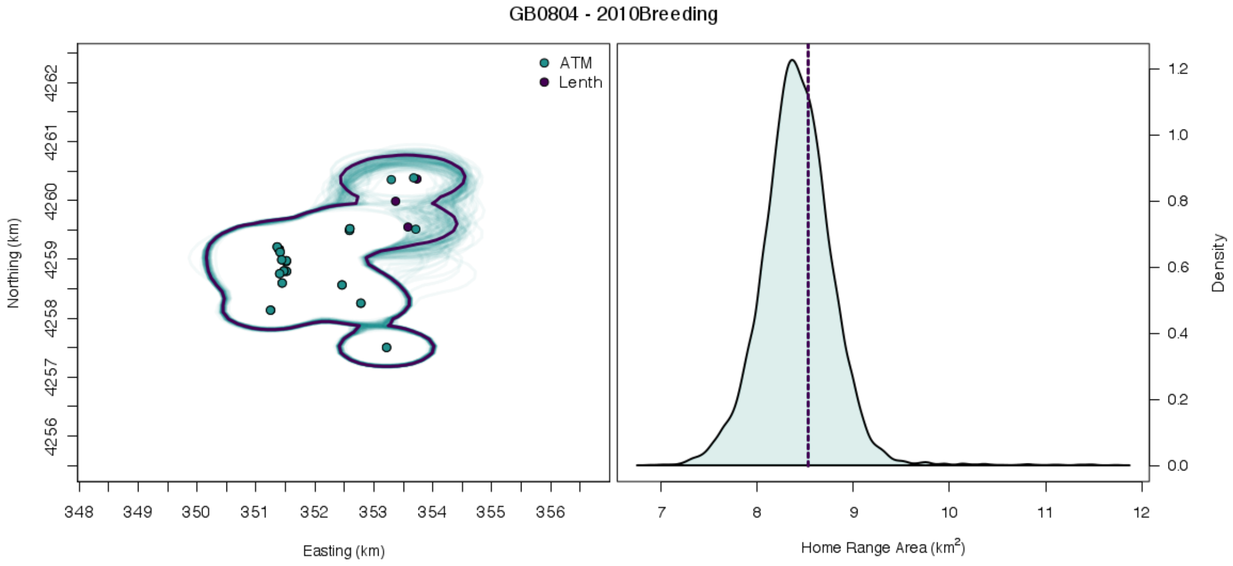
GB0804 - 2010Breeding
ATM
Lenth
Northing (km)
Easting (km)
Density
Home Range Area (km^2)

(b)

Figure 36

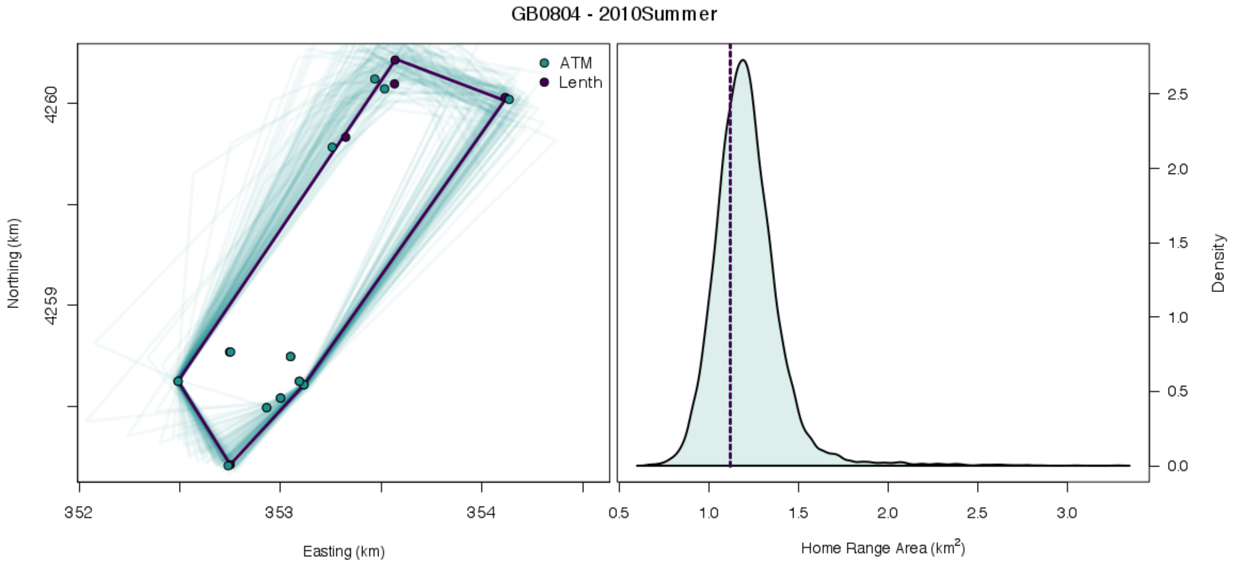
GB0804 - 2010Summer
ATM
Lenth
Northing (km)
4259
4260
352
353
354
Easting (km)
0.5
1.0
1.5
2.0
2.5
3.0
Home Range Area (km²)
0.0
0.5
1.0
1.5
2.0
2.5
Density

(a)

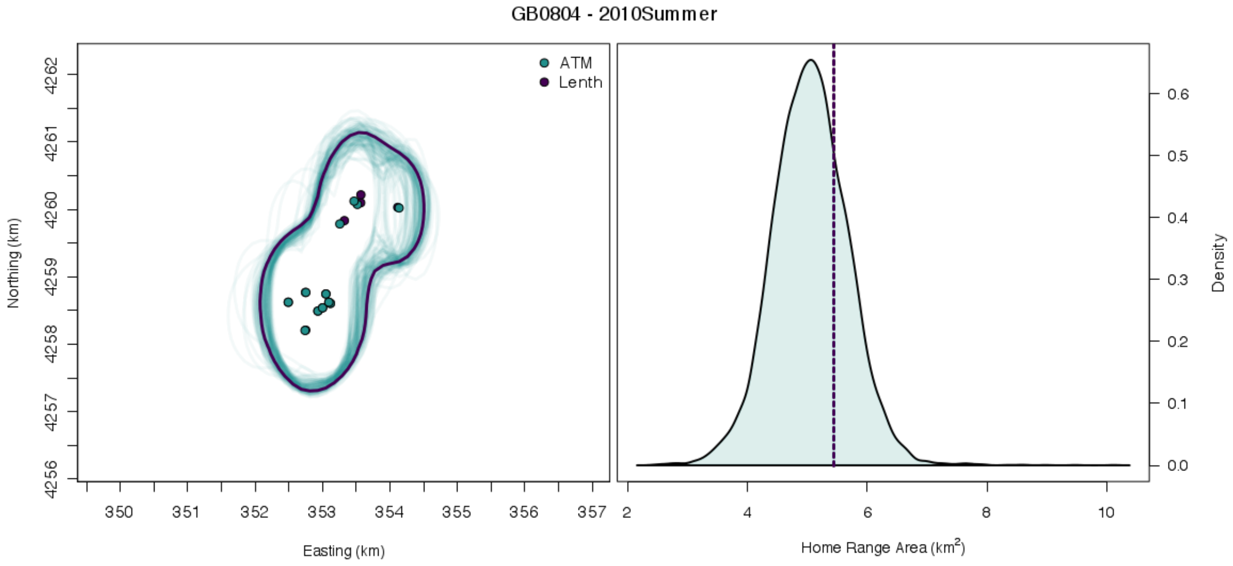
GB0804 - 2010Summer
ATM
Lenth
Northing (km)
4256
4257
4258
4259
4260
4261
4262
350
351
352
353
354
355
356
357
Easting (km)
2
4
6
8
10
Home Range Area (km²)
0.0
0.1
0.2
0.3
0.4
0.5
0.6
Density

(b)

Figure 37

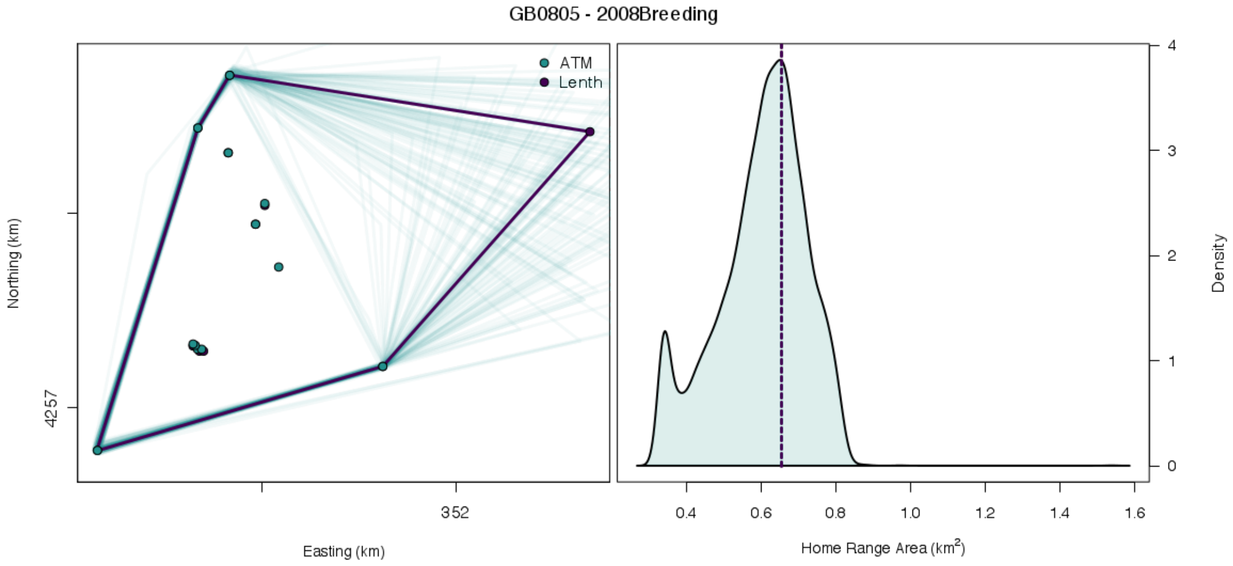

GB0805 - 2008Breeding
ATM
Lenth
Northing (km)
4257
352
Easting (km)
Density
0.4
0.6
0.8
1.0
1.2
1.4
1.6
0
1
2
3
4
Home Range Area (km2)

(a)

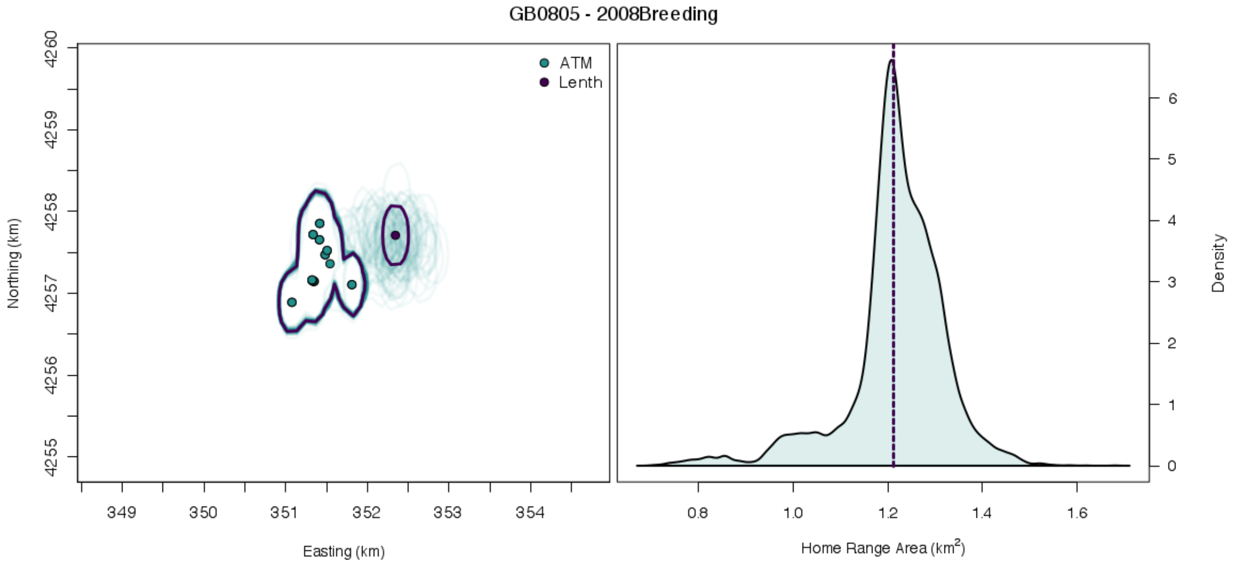

GB0805 - 2008Breeding
ATM
Lenth
Northing (km)
4255
4256
4257
4258
4259
4260
349
350
351
352
353
354
Easting (km)
Density
0.8
1.0
1.2
1.4
1.6
0
1
2
3
4
5
6
Home Range Area (km2)

(b)

Figure 38

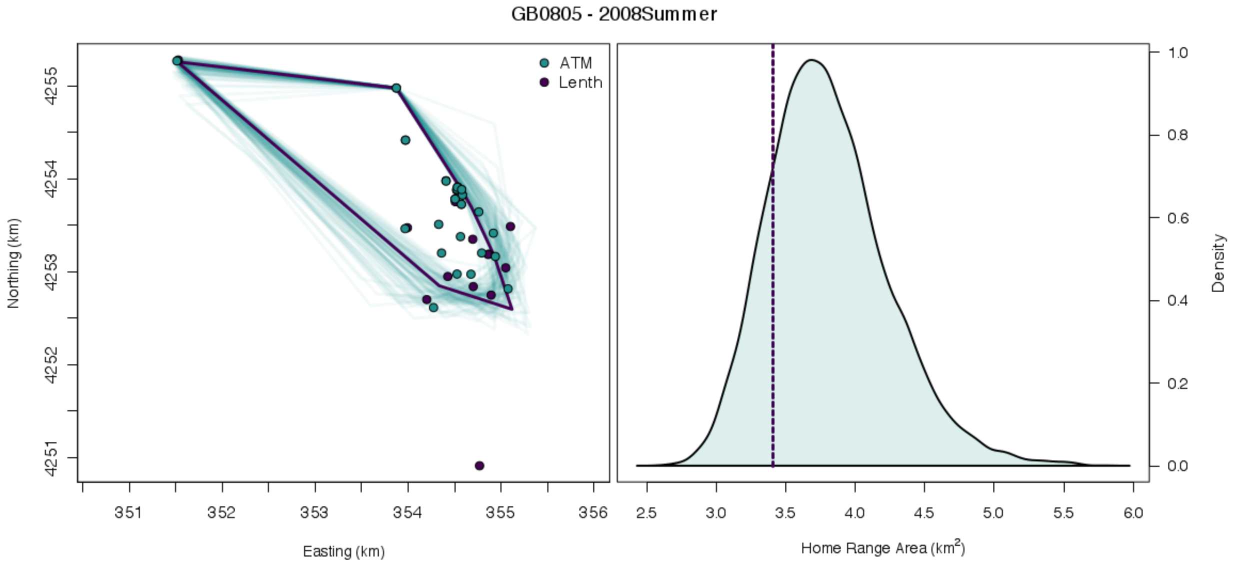
GB0805 - 2008Summer
ATM
Lenth
Northing (km)
4251
4252
4253
4254
4255
351
352
353
354
355
356
Easting (km)
2.5
3.0
3.5
4.0
4.5
5.0
5.5
6.0
Home Range Area (km²)
0.0
0.2
0.4
0.6
0.8
1.0
Density

(a)

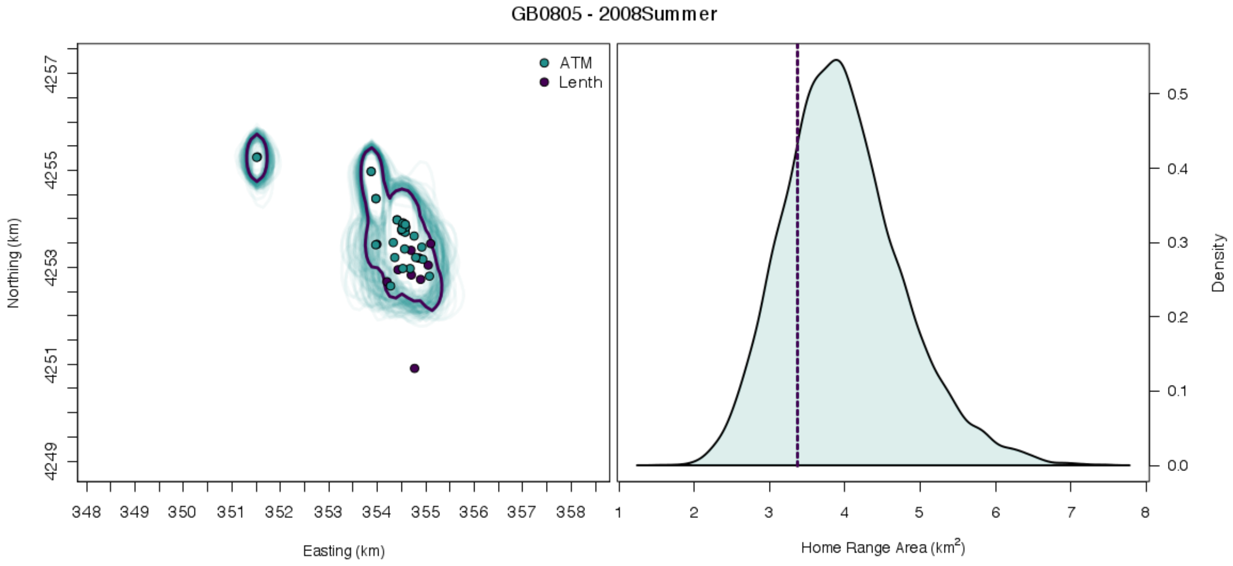
GB0805 - 2008Summer
ATM
Lenth
Northing (km)
4249
4251
4253
4255
4257
348
349
350
351
352
353
354
355
356
357
358
Easting (km)
1
2
3
4
5
6
7
8
Home Range Area (km²)
0.0
0.1
0.2
0.3
0.4
0.5
Density

(b)

Figure 39

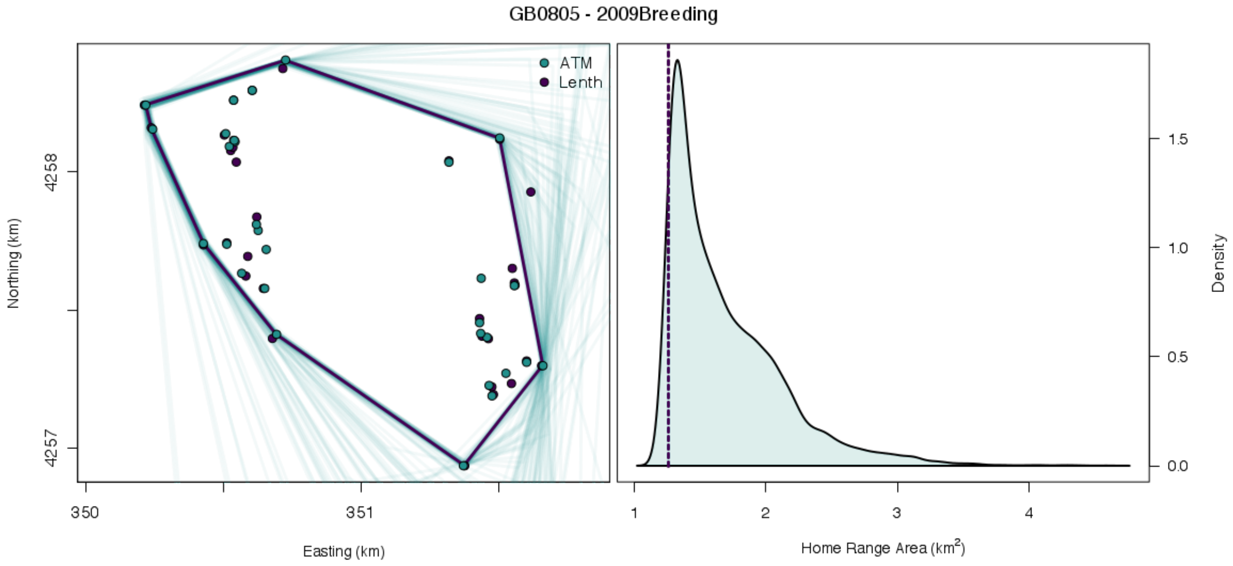
GB0805 - 2009Breeding
ATM
Lenth
Northing (km)
4258
4257
350
351
Easting (km)
1
2
3
4
Home Range Area (km^2)
Density
1.5
1.0
0.5
0.0

(a)

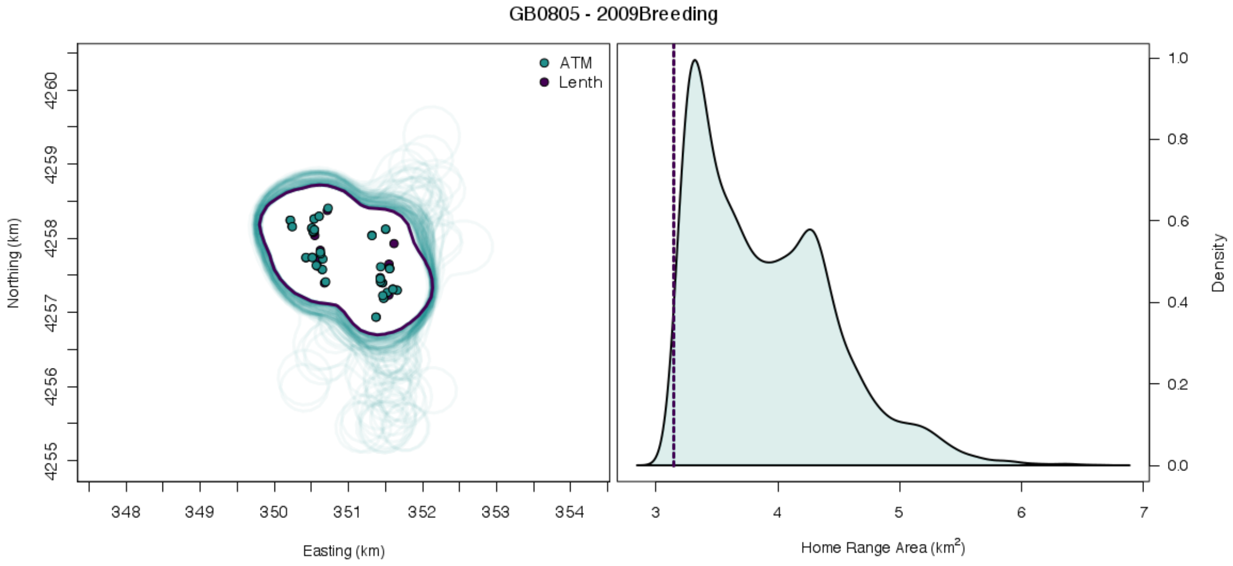
GB0805 - 2009Breeding
ATM
Lenth
Northing (km)
4260
4259
4258
4257
4256
4255
348
349
350
351
352
353
354
Easting (km)
3
4
5
6
7
Home Range Area (km^2)
Density
1.0
0.8
0.6
0.4
0.2
0.0

(b)

Figure 40

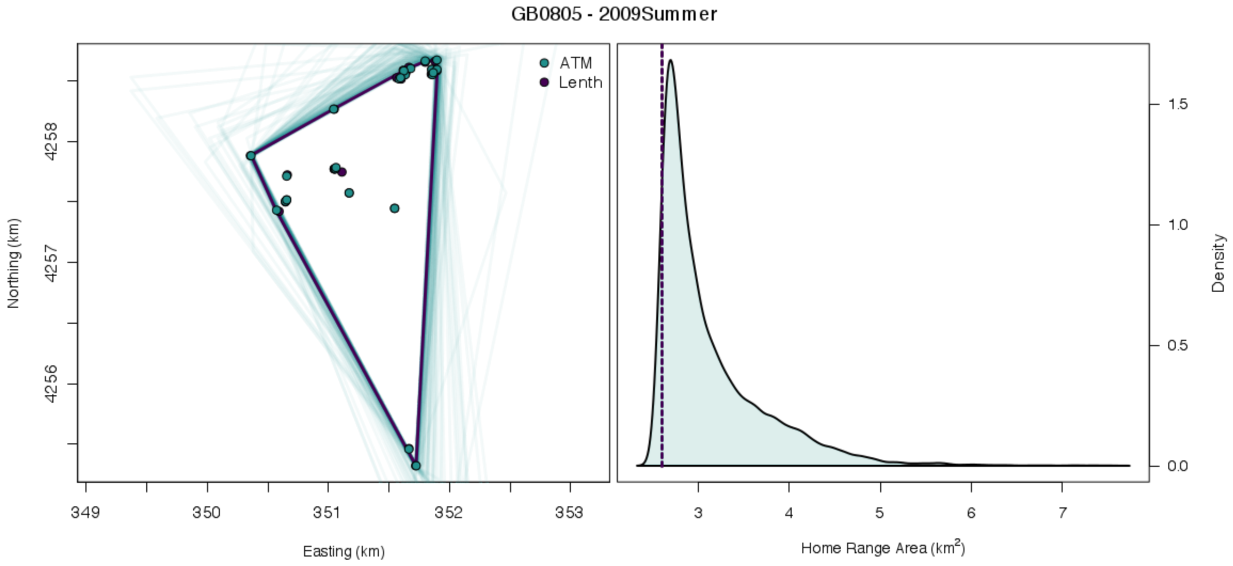
GB0805 - 2009Summer
ATM
Lenth
Northing (km)
Easting (km)
Density
Home Range Area (km²)

(a)

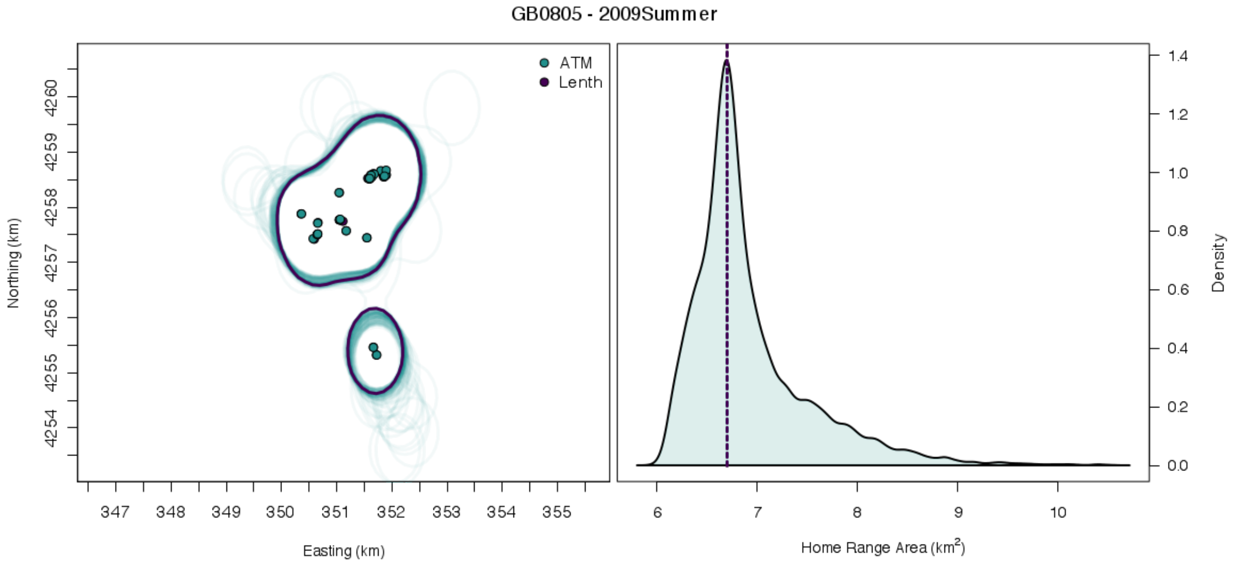
GB0805 - 2009Summer
ATM
Lenth
Northing (km)
Easting (km)
Density
Home Range Area (km²)

(b)

Figure 41

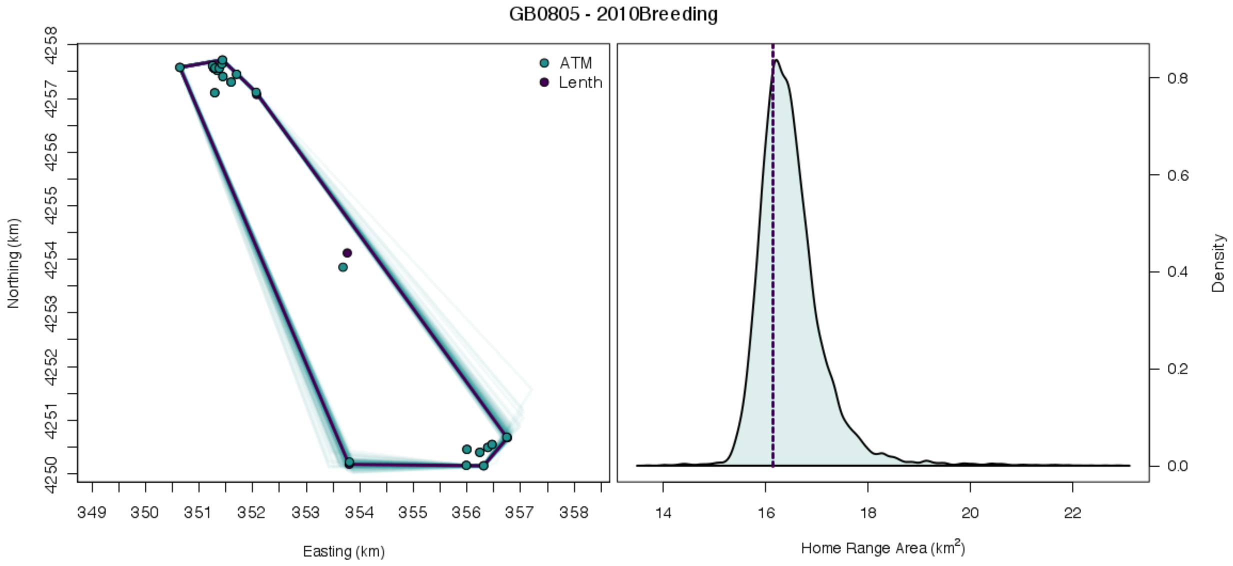
GB0805 - 2010Breeding
ATM
Lenth
Northing (km)
Easting (km)
Density
Home Range Area (km2)

(a)

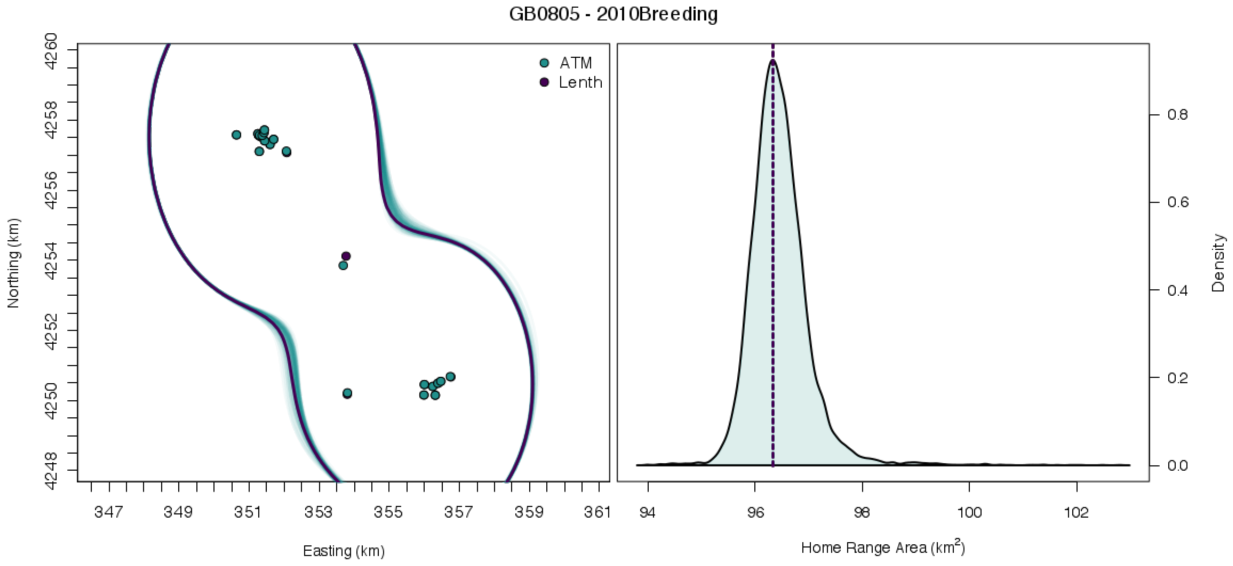
GB0805 - 2010Breeding
ATM
Lenth
Northing (km)
Easting (km)
Density
Home Range Area (km2)

(b)

Figure 42

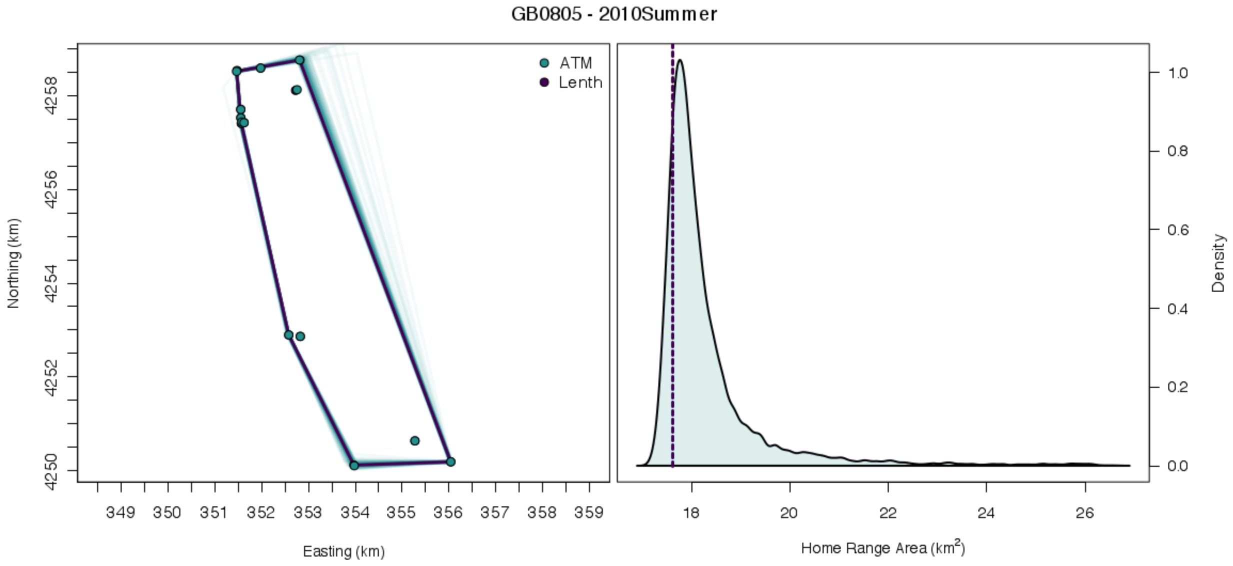
GB0805 - 2010Summer
ATM
Lenth
Northing (km)
Easting (km)
Density
Home Range Area (km^2)

(a)

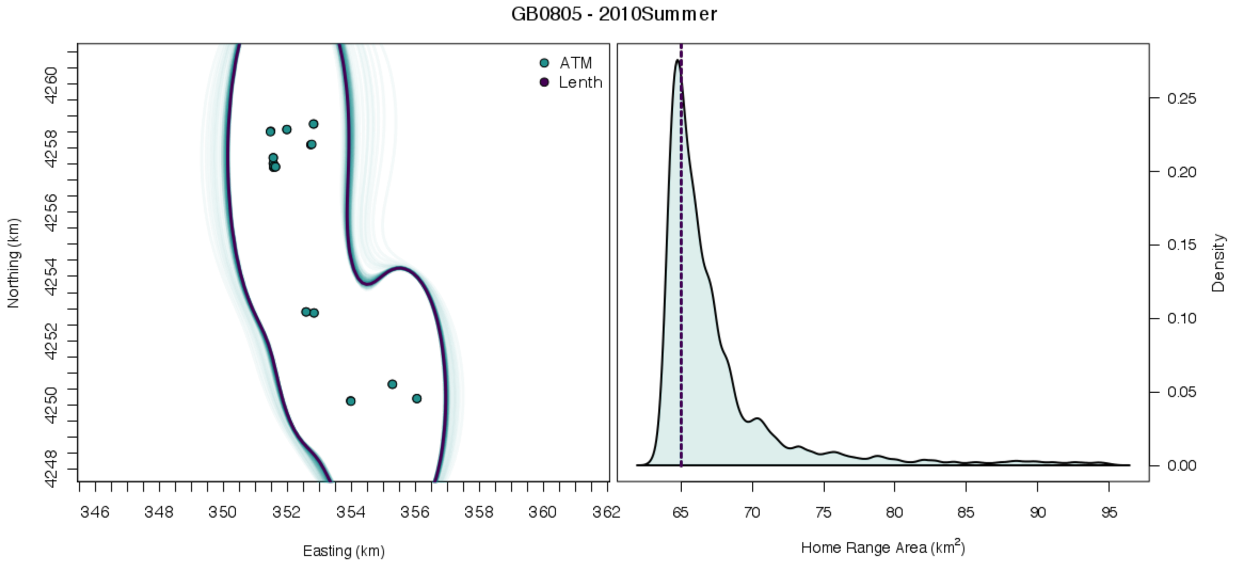
GB0805 - 2010Summer
ATM
Lenth
Northing (km)
Easting (km)
Density
Home Range Area (km^2)

(b)

Figure 43

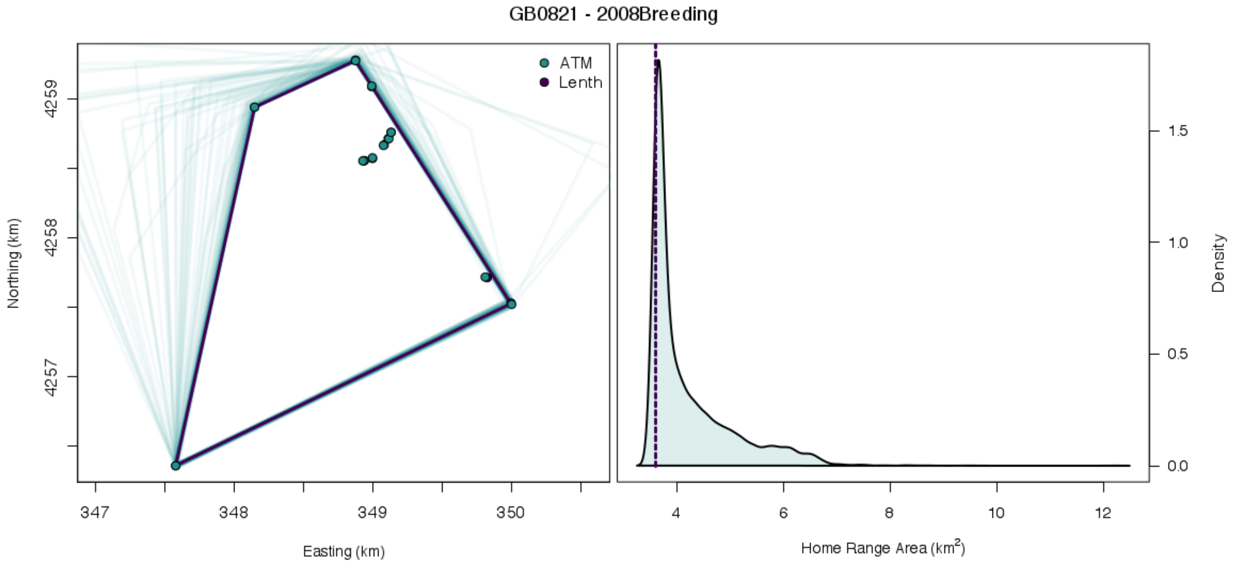
GB0821 - 2008Breeding
ATM
Lenth
Northing (km)
Easting (km)
Home Range Area (km^2)
Density

(a)

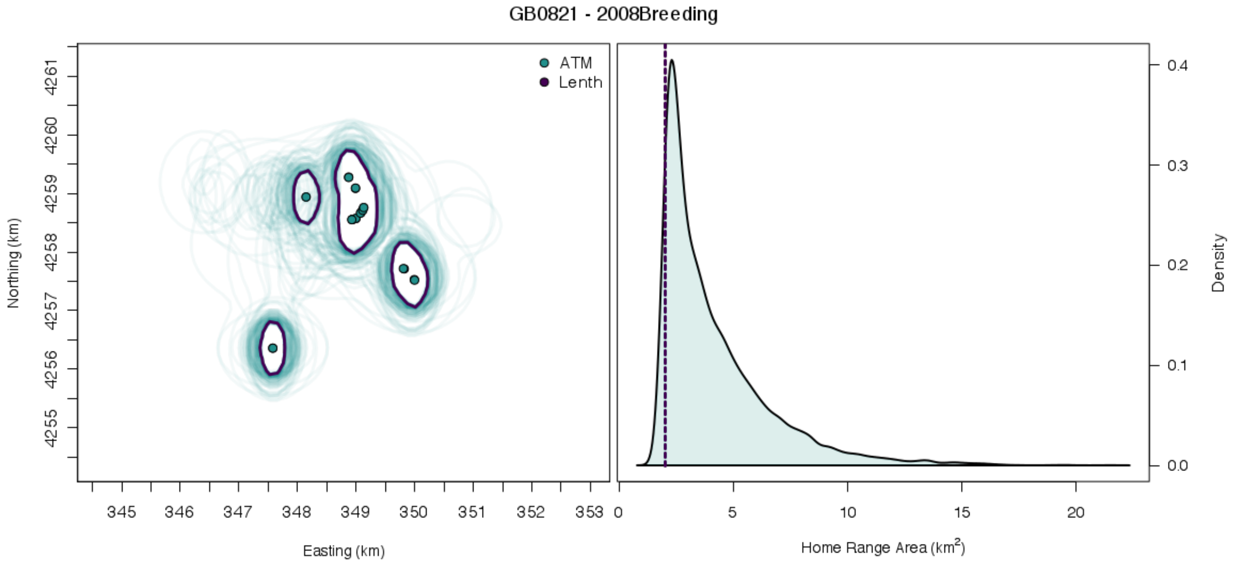
GB0821 - 2008Breeding
ATM
Lenth
Northing (km)
Easting (km)
Home Range Area (km^2)
Density

(b)

Figure 44

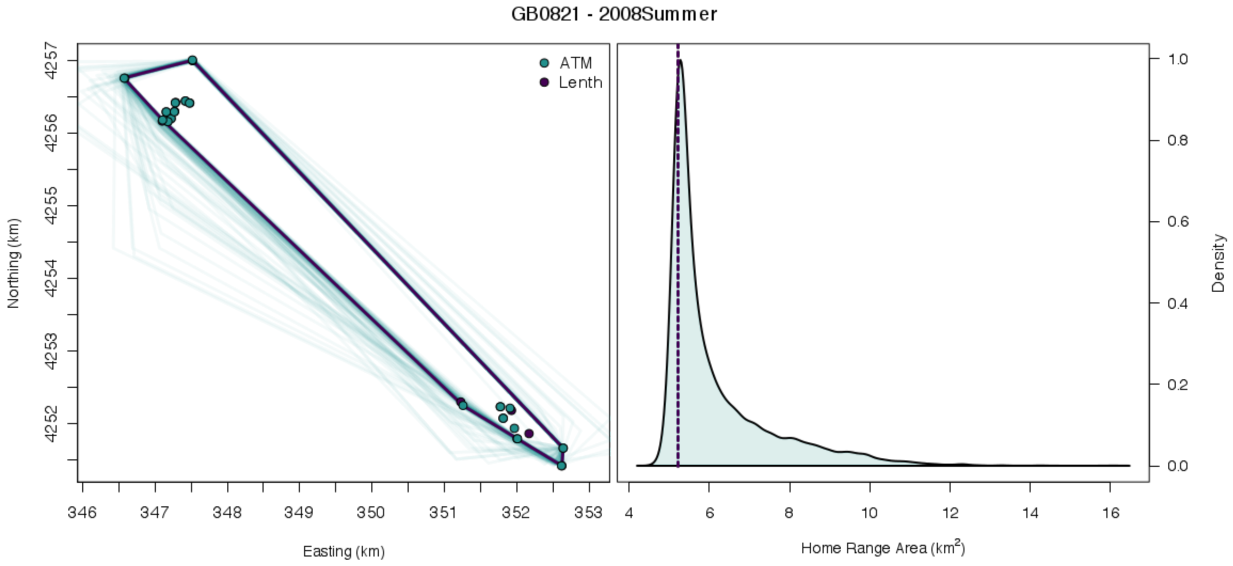
GB0821 - 2008Summer
ATM
Lenth
Northing (km)
Easting (km)
Home Range Area (km^2)
Density

(a)

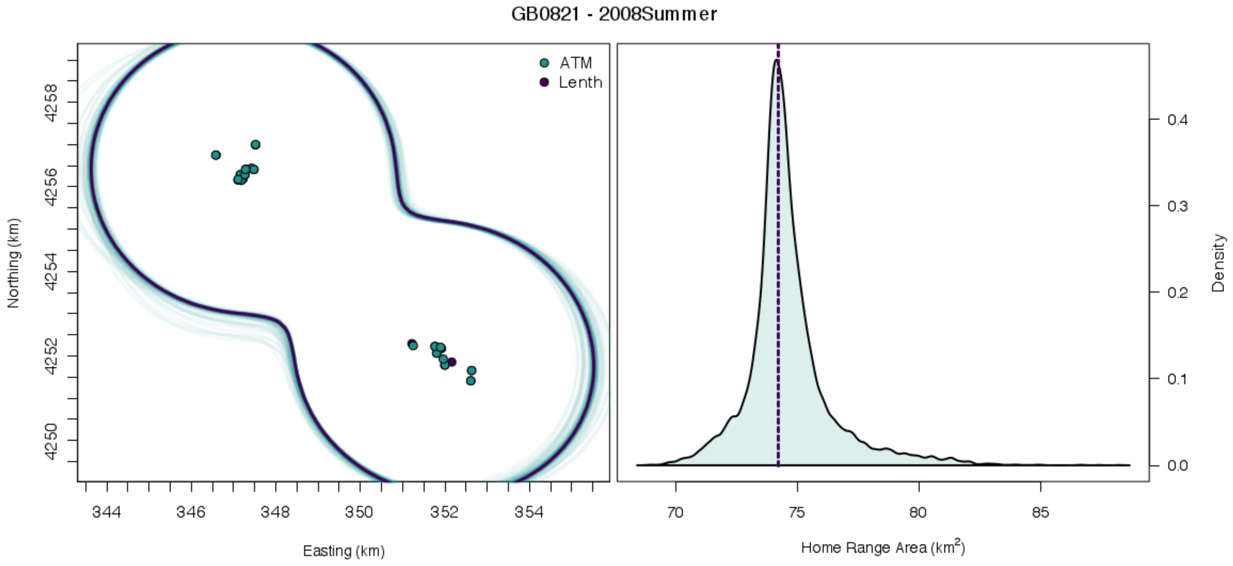
GB0821 - 2008Summer
ATM
Lenth
Northing (km)
Easting (km)
Home Range Area (km^2)
Density

(b)

Figure 45

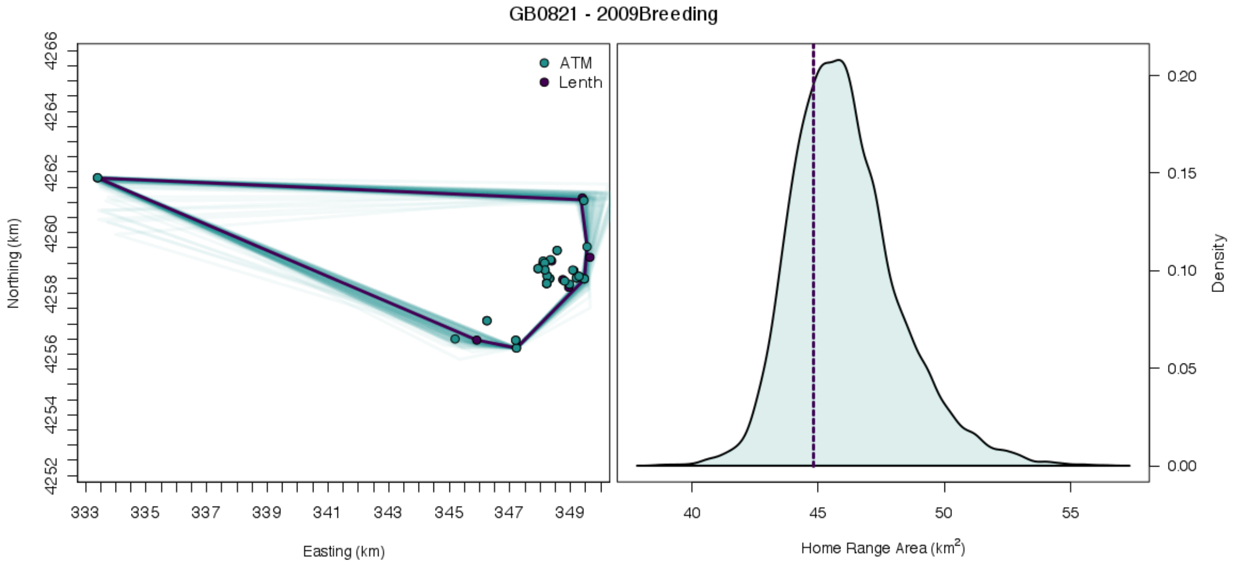
GB0821 - 2009Breeding
ATM
Lenth
Northing (km)
Easting (km)
Density
Home Range Area (km²)

(a)

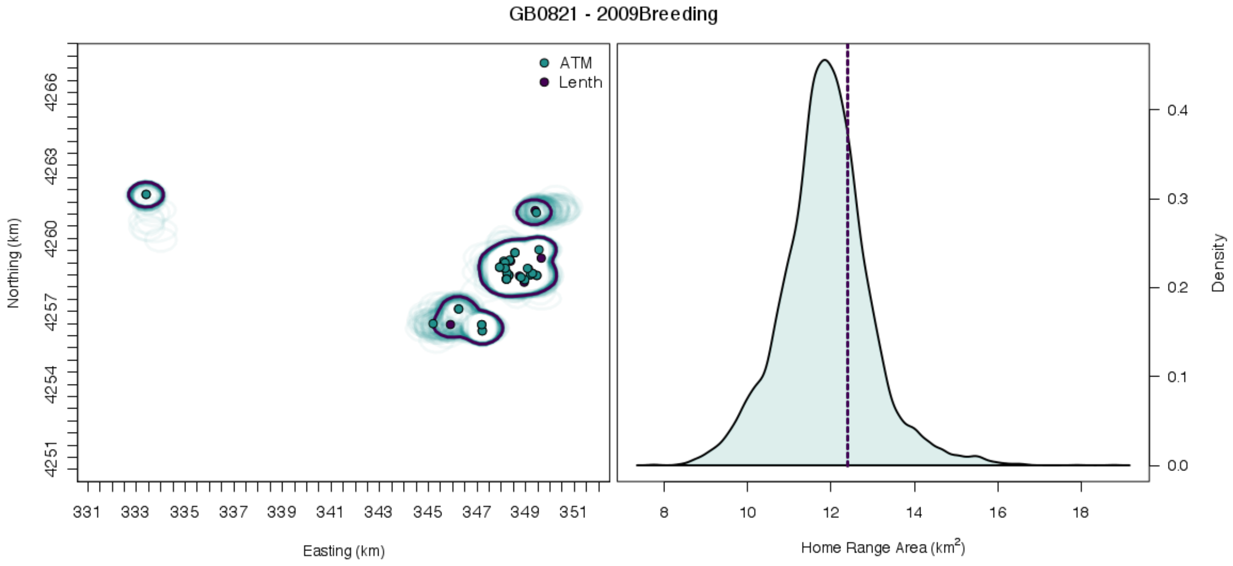
GB0821 - 2009Breeding
ATM
Lenth
Northing (km)
Easting (km)
Density
Home Range Area (km²)

(b)

Figure 46

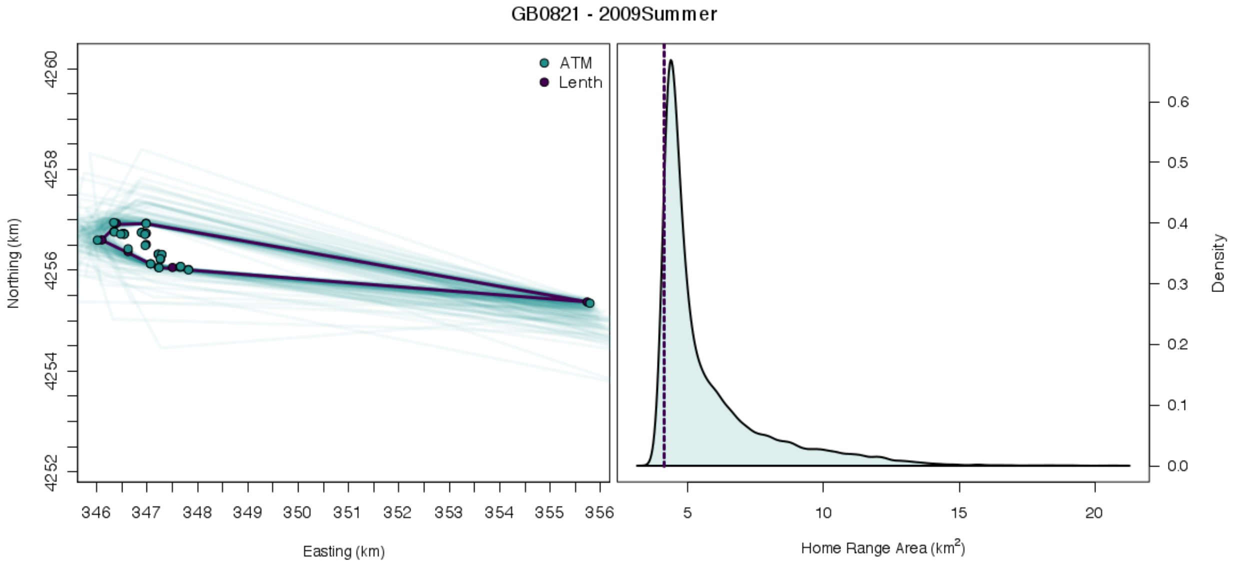
GB0821 - 2009Summer
ATM
Lenth
Northing (km)
Easting (km)
Density
Home Range Area (km²)

(a)

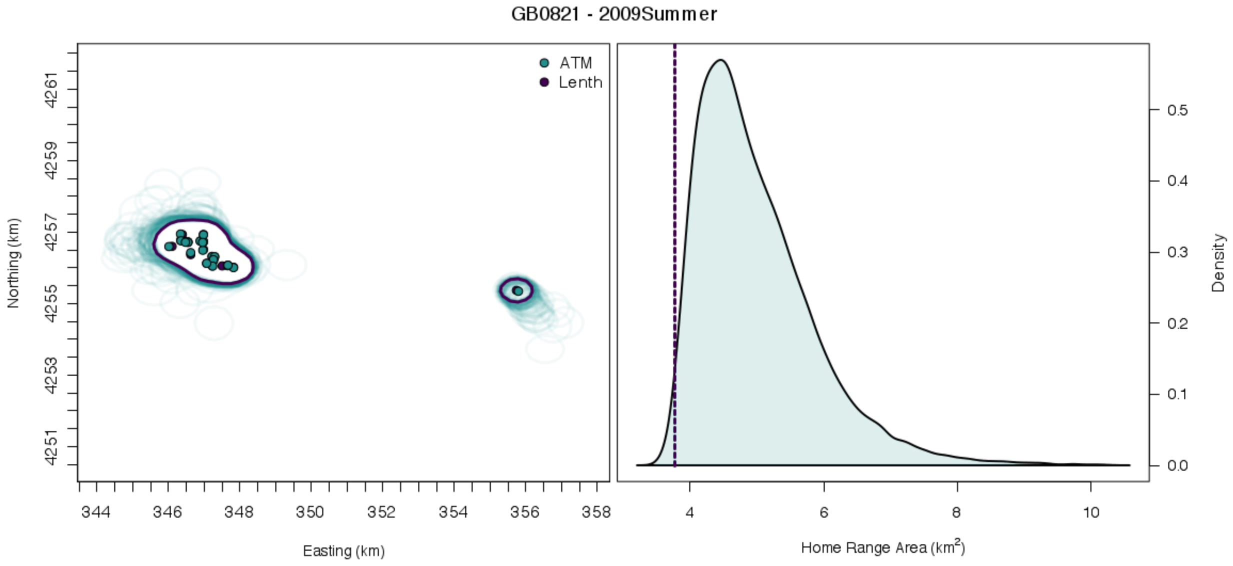
GB0821 - 2009Summer
ATM
Lenth
Northing (km)
Easting (km)
Density
Home Range Area (km²)

(b)

Figure 47

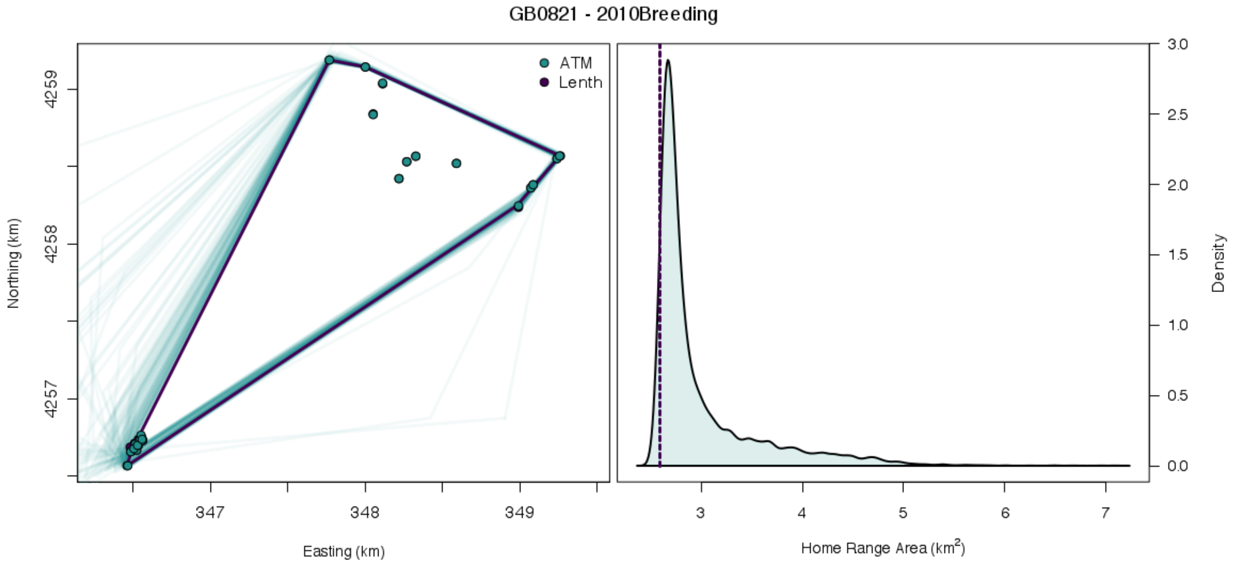
GB0821 - 2010Breeding
ATM
Lenth
Northing (km)
Easting (km)
347
348
349
4257
4258
4259
Home Range Area (km²)
Density
3
4
5
6
7
0.0
0.5
1.0
1.5
2.0
2.5
3.0

(a)

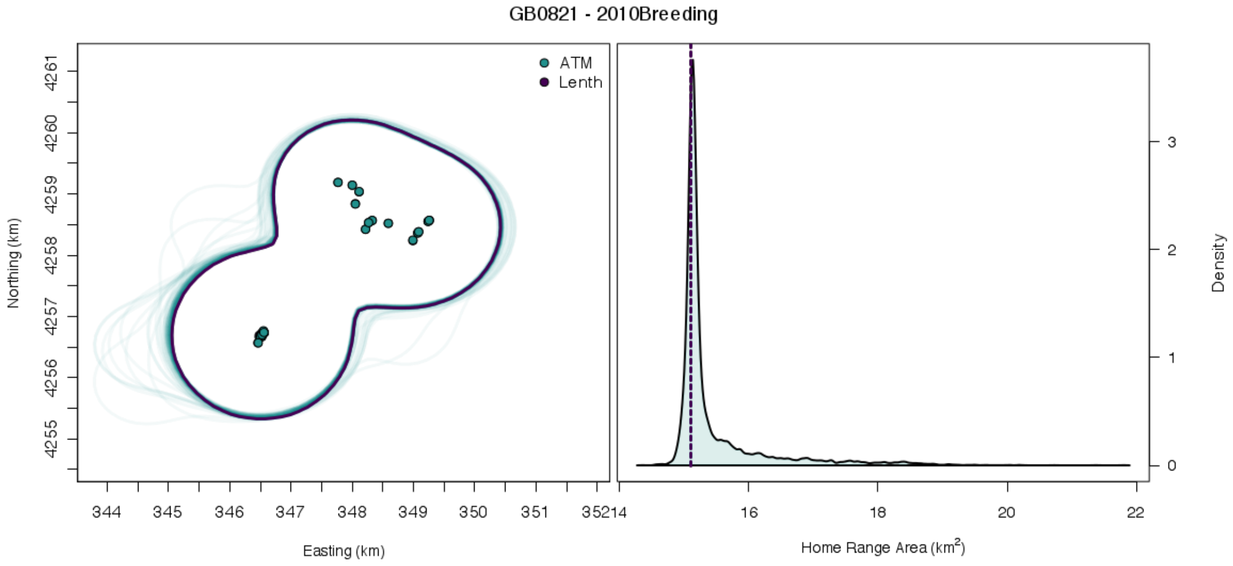
GB0821 - 2010Breeding
ATM
Lenth
Northing (km)
Easting (km)
344
345
346
347
348
349
350
351
352
4255
4256
4257
4258
4259
4260
4261
Home Range Area (km²)
Density
14
16
18
20
22
0
1
2
3

(b)

Figure 48

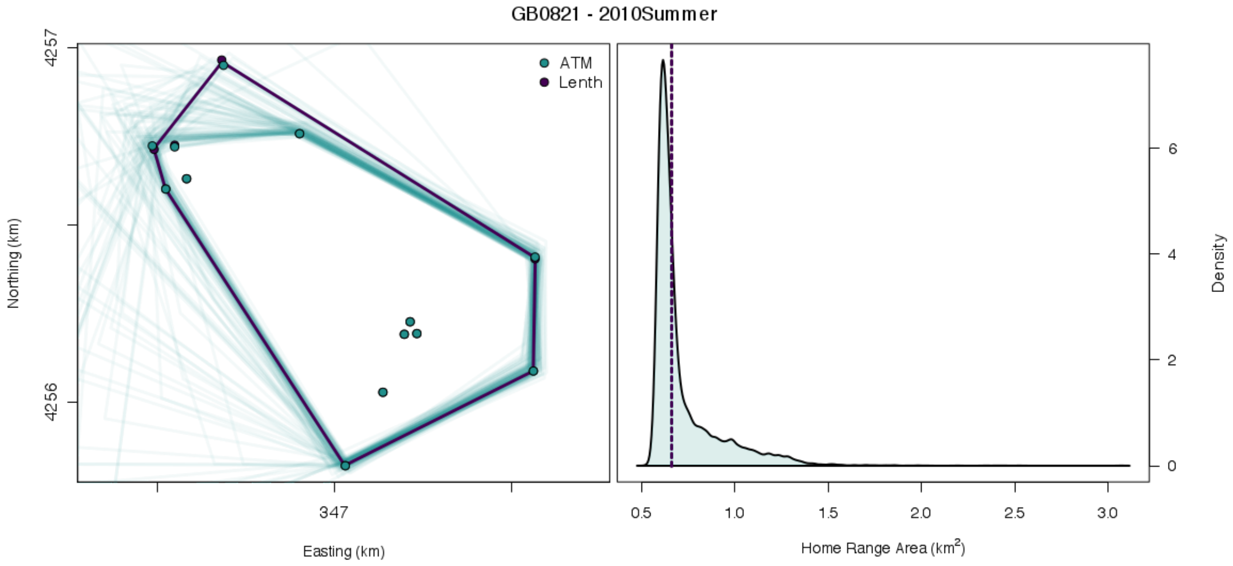
GB0821 - 2010Summer
ATM
Lenth
4257
4256
Northing (km)
347
Easting (km)
6
4
2
0
Density
0.5
1.0
1.5
2.0
2.5
3.0
Home Range Area (km²)

(a)

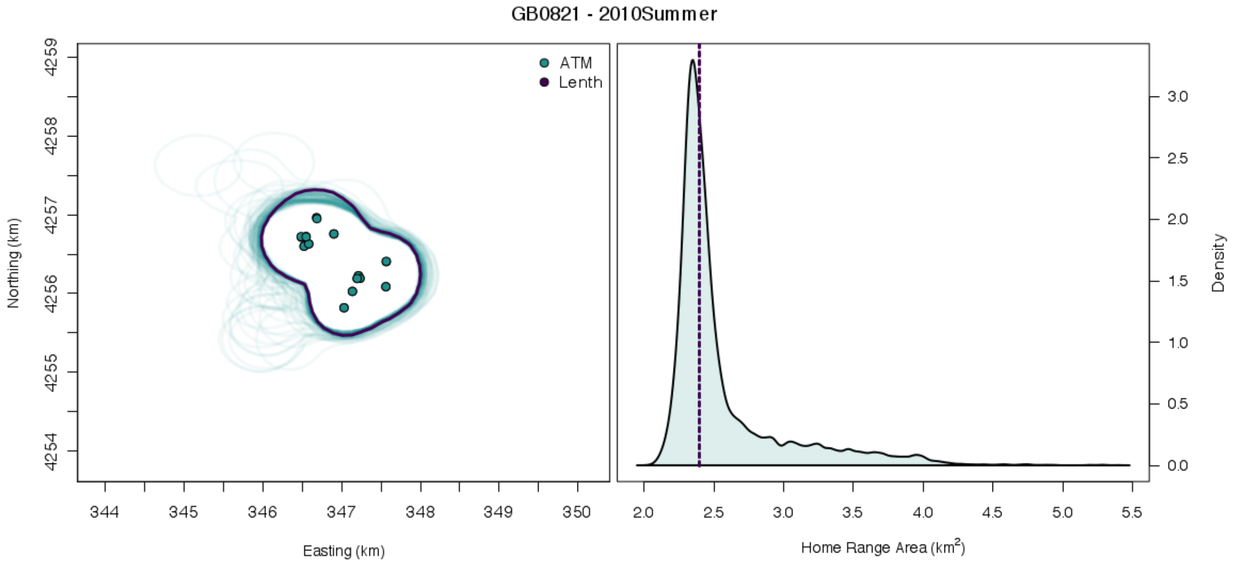
GB0821 - 2010Summer
ATM
Lenth
4259
4258
4257
4256
4255
4254
Northing (km)
344
345
346
347
348
349
350
Easting (km)
3.0
2.5
2.0
1.5
1.0
0.5
0.0
Density
2.0
2.5
3.0
3.5
4.0
4.5
5.0
5.5
Home Range Area (km²)

(b)

Figure 49
